# Retinal Expression of the *Drosophila eyes absent* Gene Is Controlled by Several Cooperatively Acting Cis-regulatory Elements

**DOI:** 10.1371/journal.pgen.1006462

**Published:** 2016-12-08

**Authors:** Bonnie M. Weasner, Brandon P. Weasner, Sarah D. Neuman, Arash Bashirullah, Justin P. Kumar

**Affiliations:** 1 Department of Biology, Indiana University, Bloomington, Indiana, United States of America; 2 Division of Pharmaceutical Sciences, University of Wisconsin, Madison, Wisconsin, United States of America; New York University, UNITED STATES

## Abstract

The *eyes absent* (*eya*) gene of the fruit fly, *Drosophila melanogaster*, is a member of an evolutionarily conserved gene regulatory network that controls eye formation in all seeing animals. The loss of *eya* leads to the complete elimination of the compound eye while forced expression of *eya* in non-retinal tissues is sufficient to induce ectopic eye formation. Within the developing retina *eya* is expressed in a dynamic pattern and is involved in tissue specification/determination, cell proliferation, apoptosis, and cell fate choice. In this report we explore the mechanisms by which *eya* expression is spatially and temporally governed in the developing eye. We demonstrate that multiple cis-regulatory elements function cooperatively to control *eya* transcription and that spacing between a pair of enhancer elements is important for maintaining correct gene expression. Lastly, we show that the loss of *eya* expression in *sine oculis* (*so*) mutants is the result of massive cell death and a progressive homeotic transformation of retinal progenitor cells into head epidermis.

## Author Summary

Activation of a gene requires interactions between enhancer and promoter elements. It has been known for some time that transcription of a gene expressed in a complex pattern or in multiple tissues is regulated by an array of enhancers. Recent studies have also demonstrated that multiple enhancers can regulate a single expression pattern within a single tissue. In this study we asked how the expression pattern of *eyes absent* (*eya*) is regulated at the level of the enhancer in the developing retina. We found that several adjacently positioned enhancer elements function cooperatively to control temporal and spatial expression of *eya* and that the spacing between two of these cis-regulatory elements is important to their function. This study shows the importance of enhancer cooperation and architecture in regulating complex and dynamically changing expression patterns.

## Introduction

Construction of a properly functioning organ or tissue is dependent upon the activity of hundreds of genes that can be conceptually organized into a gene regulatory network (GRN) [1–4]. These genes control the specification/determination, patterning, differentiation, and physiology of all cell types within the developing and adult organ. The development of the retina in the fruit fly, *Drosophila melanogaster*, is controlled in part by an evolutionarily conserved gene regulatory network called the retinal determination (RD) network [5]. The core members are two PAX6 genes, *twin of eyeless* (*toy*) and *eyeless* (*ey*), the SIX gene *sine oculis* (*so*), the EYA family member *eyes absent* (*eya*), and the SKI/SNO proto-oncogene *dachshund* (*dac*) [6–11]. In addition to these core members, the fly version of this network contains an additional nine genes of which some are functionally conserved within the vertebrate eye [5]. Mutations in the fly RD genes lead to drastic reductions of the compound eyes while forced expression in non-ocular tissues such as the wings, antennas, and legs leads to the formation of structurally complete ectopic eyes. These observations suggest that these factors occupy the highest positions within the larger eye/lens gene regulatory network. In addition to the eye, the core members are used reiteratively during development to also determine the fate of many non-ocular tissues such as the musculature, skeletal system, nose, ear, pancreas, and kidney [12–14]. Studies of the RD network can therefore provide invaluable insights into the specification and patterning of a wide range of tissues and organs beyond the eye.

The RD network has been best studied in *Drosophila* with a quarter century of investigation having identified a wealth of genetic, biochemical, and molecular interactions amongst the different members. Numerous review articles over the years have summarized these findings in static circuit maps [5,12,15–17]. While these interaction diagrams have been helpful in understanding the relationship amongst network members, they can be misleading since the network genes are expressed in dynamic patterns that change both spatially and temporally [18]. In addition, individual genes initiate expression at different times in development [6,8,9,11,17,19], are co-expressed with other network genes in some cells but not in others [18], and appear to interact differently depending upon the exact spatial, temporal, and developmental context [20,21]. As a result the static maps of regulatory interactions do not necessarily reflect the reality of what is happening throughout the eye in either space or time. In this report, we have focused on understanding how, at the level of cis-regulatory elements, the *eya* gene is regulated temporally and spatially in the developing retina. We then use this information to evaluate one tenant of the RD circuit map–namely we test the potential regulation of *eya* by the So transcription factor.

The Eya protein functions as a transcriptional co-activator and protein tyrosine phosphatase [22–24], although the latter activity appears dispensable for eye development in *Drosophila* [25]. Within the nucleus Eya interacts with members of the SIX/So family of homeodomain containing DNA binding proteins [22]. Together, SIX-EYA complexes function as bipartite transcription factors to activate targets necessary for the specification, differentiation, and growth of the retina [22,23,26]. Recent reports indicate that these complexes also function as transcriptional repressors although the exact mechanism underlying this activity has yet to be determined [19,20,27,28]. Both genes are expressed in nearly identical spatial patterns within the developing eye [10,11]. Expression of both genes is lost in both *eya* and *so* mutants [29]. These properties have led to the proposal that the So-Eya complex regulates the expression of both genes.

In the wild type eye *eya* expression is temporally and spatially dynamic [11]. This expression is completely eliminated from the retina of *eya*^*2*^ mutants, which are viable but completely lack the adult compound eyes [11]. These flies harbor a 322bp deletion, which lies 576bp upstream of the transcriptional start site [11]. When multimerized this 322bp fragment drives expression of a transcriptional reporter in a pattern that approximates the wild type gene [30,31]. It also contains sufficient activity to partially restore eye development to *eya*^*2*^ mutants when driving expression of a rescuing transgene [30,31]. Based on this evidence this enhancer, for many years, was thought to be the sole cis-regulatory element controlling *eya* expression within the developing eye. Sequence analysis identified the presence of a canonical So binding site within this enhancer thereby raising the possibility that *eya* expression in the eye is controlled by So [31,32]. More recently, several studies of the *eya* locus have identified two additional retinal enhancers, the presence of additional So binding sites, and multiple genomic positions where So appears to bind in eye-antennal discs [33–35]. Together these data have been used to support the premise that the initiation and maintenance of *eya* expression is under the control of So.

In this paper we report the identification of several cis-regulatory elements within the *eya* locus that contribute to its expression in the developing eye. Three of these enhancers lie adjacent to each other and we demonstrate that they function cooperatively to regulate the temporal and spatial expression pattern of *eya* during eye development. We also show that the spacing between two of these enhancers is important for the activity of each cis-regulatory element. And finally, we show that each of the retinal enhancers (those identified in this and other studies) remain active in *so* loss-of-function mutants. This is at odds with the model in which *eya* is regulated by So. We show that the loss of *eya* expression in *so* mutants is actually the result of cell death and a progressive fate transformation of the retina into head epidermis. Our findings do not support a role for So in the initiation of *eya* expression. However we do not rule out the possibility that So functions to maintain *eya* transcription in the retina.

## Results

### So-VP16 partially restores *eya* expression and rescues *eya*^*2*^ mutants

**Fig 1:**
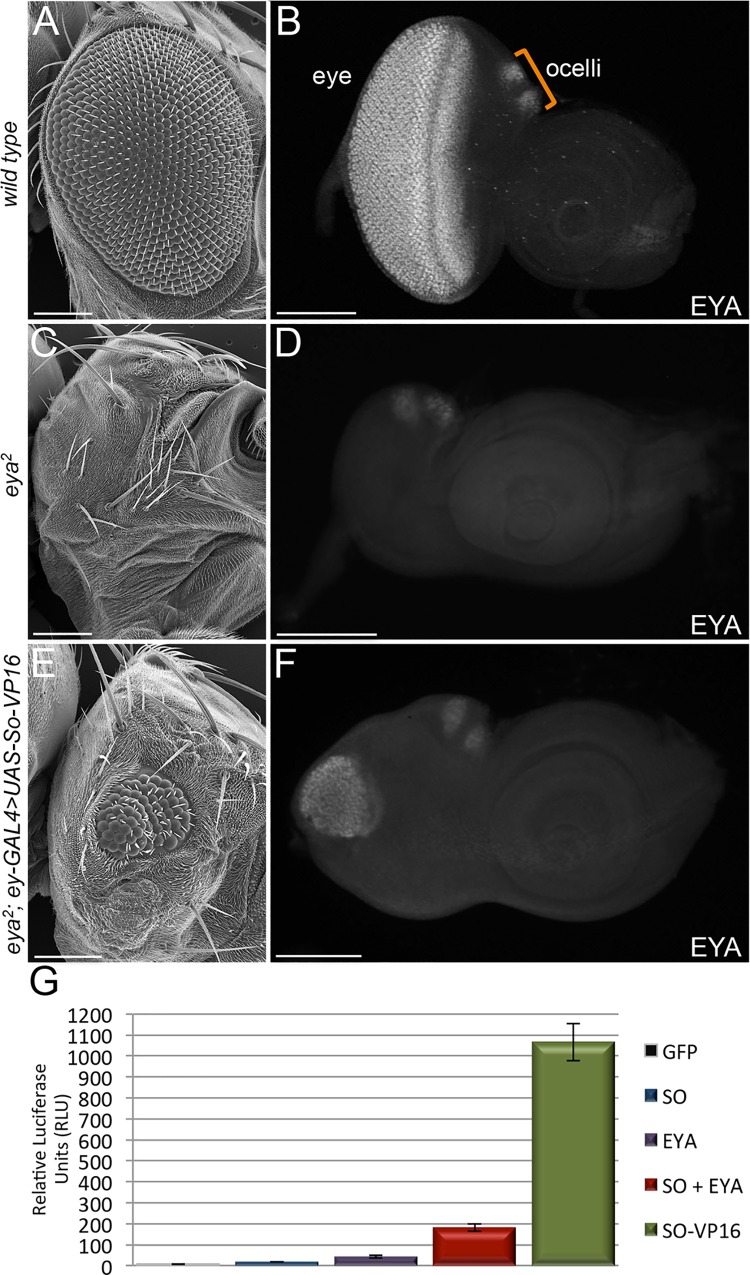
So-VP16 reactivates *eya* expression in the retina of *eya*^*2*^ mutants. (A,C,E) SEM images of adult female *Drosophila* compound eyes and heads. (A) *wild type*. (C) *eya*^*2*^. (E) *eya*^*2*^ mutants in which expression of UAS-So-VP16 partially restores eye development. (B,D,F) Light microscope images of third instar eye-antennal discs—*eya* expression is detected by antibody staining against Eya protein. (B) Wild type expression of *eya* in the compound eye and ocelli. (D) Loss of *eya* expression in the eye portion of disc in *eya*^*2*^ mutants. Expression with the ocelli is maintained in the mutant. (F) Partial restoration of *eya* expression in the eye portion of disc upon expression of UAS-So-VP16. Anterior is to the right in adult head and imaginal disc images. At least 30 adult flies and developing imaginal discs were examined for each genotype with 57 adult *eya*^*2*^*; ey-GAL4*, *UAS-So-VP16* flies being scored for rescue of eye structure (G) Luciferase assay quantifying activation strength of So-VP16. Y-axis is relative luciferase units (RLU). Three biological replicates were conducted for each experiment. Error bars in panel G represent standard deviation. Scale bar, 100μm

In third larval instar retinas *eya* is expressed in a small stripe of cells ahead of the advancing morphogenetic furrow, in differentiating photoreceptor, cone, and pigment cells, and in the developing ocelli (Fig 1A and 1B) [11]. In the *eya*^*2*^ mutant *eya* expression is completely lost from the eye field (Fig 1C and 1D). We first set out to determine if the So consensus sites and regions of So ChIP peaks that are found outside of the original 322bp enhancer are functional. To do this we attempted to rescue the *eya*^*2*^ mutant by forcibly expressing a So-VP16 chimeric construct in the developing eye with an *ey-GAL4* driver. This protein is capable of fully restoring eye development to *so*^*1*^ mutants [27] and activates a luciferase reporter at levels that are 20-fold higher than So alone and 5-fold higher than the So-Eya complex (Fig 1G). Based on these data we reasoned that So-VP16 serves as a strong transcriptional activator and therefore is a suitable substitute for the So-Eya complex (So-VP16 = So-Eya). Expression of So-VP16 partially restores both *eya* expression and eye development to 62% of the 57 animals that we examined (Fig 1E and 1F; S1A–S1C Fig). Consistent with being a very weak activator, expression of wild type So alone is insufficient to restore either *eya* expression or eye development to *eya*^*2*^ mutants (Fig 1G; S1D and S1E Fig) [27]. These results led us to initially conclude that additional So-responsive enhancer element(s) are present within the *eya* locus.

### Newly identified regulatory elements are dynamically regulated during larval eye development

**Fig 2:**
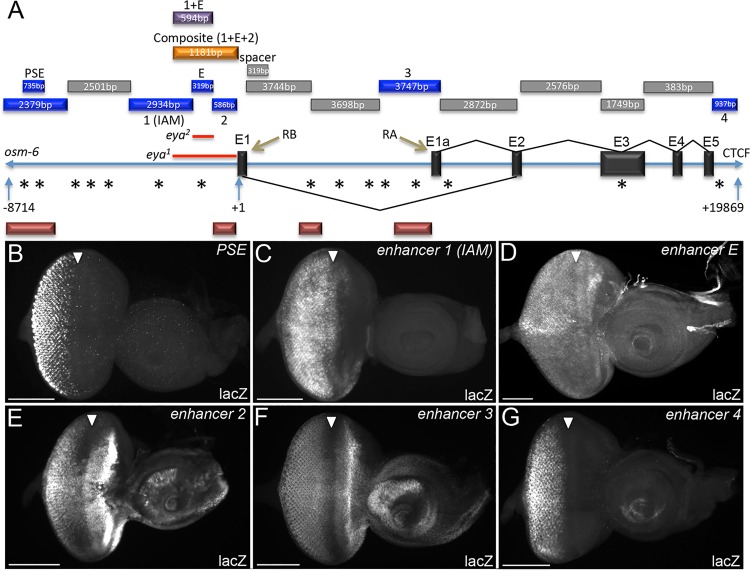
Multiple enhancers control expression of *eya* in the developing eye. (A) Illustration of the genomic map of the *eya* locus—representation is not to scale. Sequences and genomic location of each fragment are provided in supplementary materials and methods S1-3. Size of each fragment (bp) is indicated within each bar. Blue bars = individual retinal enhancers. PSE = photoreceptor specific enhancer previously identified and named by Graeme Mardon’s group in [20]. E = 319bp extant enhancer previously identified in [11]. Enhancer 1 (IAM) = immediately ahead of morphogenetic furrow enhancer was previously identified and named by Graeme Mardon’s group in [20]. We refer to this enhancer as 1 as it shows a different expression pattern than previously reported. 2–4 represent newly identified enhancer elements. Orange bar = composite enhancer, purple bar = enhancer 1+E, grey bars indicate regions that do not drive expression in the retina including the fragment used as the 319bp spacer, asterisks = So binding sites, red bars = regions of So ChIP peaks. *eya*^*1*^ and *eya*^*2*^ deletions are indicated by red lines immediately ahead of exon 1 (B-G) Light microscope images of third instar eye-antennal discs. All images represent lacZ reporter expression in a wild type genetic background. LacZ reporter activation is indicated by antibody staining against β-galactosidase. White arrowheads mark the position of the morphogenetic furrow. (B) The PSE enhancer drives expression of the reporter only in cells that lie posterior to the morphogenetic furrow. (C) Enhancer 1 (also called IAM) drives expression in cells ahead and behind the morphogenetic furrow. (D) The 319bp extant enhancer drives weak reporter expression in cells ahead and posterior to the morphogenetic furrow. (E) Enhancer 2 drives expression of the reporter in cells anterior and posterior to furrow. (F) Enhancer 3 drives expression in cells ahead and behind the morphogenetic furrow. (G) Enhancer 4 drives expression only in cells posterior to the morphogenetic furrow. No single enhancer element fully recapitulates endogenous Eya expression. Anterior is to the right in imaginal disc images. At least 30 imaginal discs were examined for each genotype. Scale bar, 100μm

In order to identify regulatory elements that are responsive to the So-Eya complex we used the *osm-6* gene and a CTSF insulator site to define the 5`and 3`boundaries respectively of the *eya* locus and then cloned fragments of DNA between these two genomic markers ahead of a minimal hsp70 promoter and a lacZ reporter (Fig 2A). These constructs were inserted into the same genomic coordinates (*attP-3BVK00033*—cytological position 65B2) using the PhiC31 integrase system to maintain similar expression levels across reporters. Wandering third instar eye-antennal imaginal discs were then examined for lacZ reporter expression. We identified six genomic fragments that are capable of driving expression of the reporter in portions of the endogenous *eya* pattern (Fig 2B–2G). Three of these fragments (PSE, 1, and E) have been previously identified as enhancers controlling *eya* expression in the retina [30,31,35]. The PSE, which stands for **p**hotoreceptor **s**pecific **e**nhancer, drives expression solely in cells behind the morphogenetic furrow (Fig 2A and 2B) [35] while fragment 1 (also called IAM for **i**mmediately **a**nterior to the **m**orphogenetic furrow) drives expression ahead of the advancing morphogenetic furrow and in differentiating cells (Fig 2A and 2C) [35]. Fragment E (for extant) is the enhancer that is deleted in *eya*^*2*^ mutants (Fig 2A and 2D) [30,31]. Our sequence analysis indicates that the fragment is 319bp in length (and not 322bp as originally reported). Fragments 2, 3 and 4 are three new retinal enhancers that control *eya* expression in the developing eye (Fig 2A and 2E–2G).

**Fig 3:**
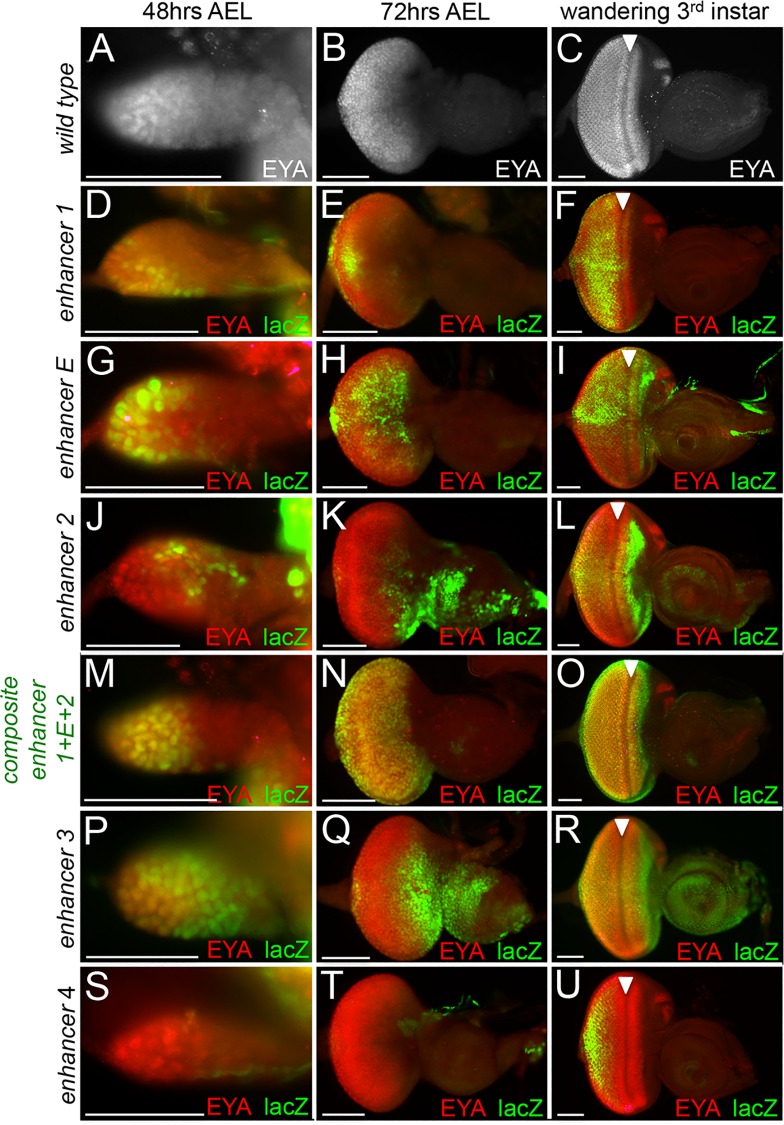
The composite enhancer controls all *eya* expression within the developing eye. (A-U) Light microscope images of developing eye-antennal discs. Images of imaginal discs at 48hrs and 72hrs AEL were taken at 20X while images of wandering third instar larvae were taken at 10X. AEL = after egg laying. Red = Eya protein, green = β-galactosidase, yellow = positions of co-localization between Eya and β-galactosidase. Arrowhead marks the position of the morphogenetic furrow. All enhancer-lacZ reporters are placed in a wild type genetic background. (A-C) Localization of Eya protein in developing wild type retinas at different developmental time points. (D-F) Enhancer 1 dependent expression is activated in a few Eya expressing cells early in development and co-localizes with Eya posterior to the morphogenetic furrow late in development. (G-I) Extant enhancer dependent expression co-localizes with Eya and is robust early in development but becomes weaker and sparse as development proceeds. (J-L) Enhancer 2-dependent expression is largely present in non-*eya* expressing cells early in development. Co-localization with Eya can be seen in cells anterior and posterior to the furrow later in development but a significant portion of reporter expression still present in non-*eya* expressing cells. (M-O) Composite enhancer-dependent expression shows co-localization with Eya protein throughout all stages of larval eye development. This is the only construct to fully recapitulate temporal and spatial *eya* expression. (P-R) Enhancer 3-dependent expression is largely present in non-*eya* expressing cells throughout development. Some co-localization with Eya protein is seen at later stages in cells anterior and posterior to the furrow. (S-U) Enhancer 4-dependent expression co-localizes with a few Eya expressing cells posterior to the furrow late in development. Anterior is to the right in imaginal disc images. At least 30 imaginal discs were examined for each genotype and developmental time point. Scale bar, 50μm

We next determined the temporal and spatial expression patterns of each individual fragment and compared these patterns to endogenous *eya* expression. Eya protein is present in the wild type eye disc as early as 48hrs AEL (early 2^nd^ instar, Fig 3A) and continues to be expressed broadly at 72hrs AEL (early 3^rd^ instar, Fig 3B). By the late third larval instar stage *eya* expression is restricted to a narrow band of cells ahead of the morphogenetic furrow and to all differentiating photoreceptor and cone cells (Fig 3C). No single individual fragment fully recapitulates the endogenous *eya* expression pattern. For example, reporter expression driven by fragment 1 is temporally and spatially delayed compared to wild type *eya* expression meaning that although it is activated in a few *eya* expressing cells early in development, it is not until late third instar that expression starts to coincide with the spatial pattern of endogenous *eya* (Fig 3D–3F, Table 1). In contrast, while reporter expression driven by fragment E coincides with early endogenous *eya*, its late expression is weak in intensity and appears mottled (Fig 3G–3I, Table 1). Lastly, the bulk of fragment 2 driven expression within younger discs is in *eya* negative cells while in later discs reporter expression does coincide with the endogenous *eya* gene (Fig 3J–3L, Table 1).

**Table 1:** Enhancer Expression and Rescue. For each rescue experiment two-three female eyes were photographed with a scanning electron micrograph. We manually counted the number of ommatidia for each eye and calculated both averages and standard deviations (listed within table). The raw ommatidia counts for the rescue experiments are as follows: *eya*^*1*^*; enhancer 1—eya RB cDNA* (141, 235, 190), *eya*^*2*^*; enhancer 1—eya RB cDNA* (322, 245), *eya*^*1*^*; enhancer E—eya RB cDNA* (39, 38, 35), *eya*^*2*^*; enhancer E—eya RB cDNA* (358, 372, 362), *eya*^*1*^*; enhancer 1+E+2—eya RB cDNA* (792, 730, 762), *eya*^*2*^*; enhancer 1+E+2—eya RB cDNA* (757, 825, 829), *eya*^*1*^*; enhancer 1+E—eya RB cDNA* (376, 324, 298), *eya*^*2*^*; enhancer 1+E—eya RB cDNA* (635, 584, 636), *eya*^*1*^*; enhancer 1+spacer+2—eya RB cDNA* (433, 433, 432), *eya*^*2*^*; enhancer 1+spacer+2—eya RB cDNA* (443, 448, 556).

Enhancer	LacZ Reporter Expression			cDNA Rescue Average # of ommatidia	
	48hrs AEL	72hrs AEL	Late 3rd Instar	*eya*^*1*^	*eya*^*2*^
Enhancer 1	-	†	‡	189 ± 47	284 ± 54
Enhancer E	‡	‡	†	37 ± 2	364 ± 7
Enhancer 2	†	†	†	-	-
Enhancer 1+E+2	‡	‡	‡	761 ± 31	804 ± 40
Enhancer 1+E	‡	‡	‡	333 ± 40	618± 30
Enhancer 1+2	-	-	†	-	-
Spacer alone	N/A	N/A	-	N/A	-
Enhancer 1+spacer+2	†	†	‡†	432 ± 1	482 ± 64
Enhancer 1+5bp+2	†	†	‡†	-	-
Enhancer 3	†	†	†	-	-
Enhancer 4	-	-	†	-	-

- No expression or rescue

‡ Recapitulates eya expression

† Expresses in non-eya+ cells or only in a few Eya+ cells

Since each of these three fragments (1,E,2) does mimic a specific temporal and/or spatial aspect of *eya* expression we hypothesized that these enhancers, which lie adjacent to each other, might function cooperatively to control all temporal and spatial aspects of *eya* expression. To test this model we generated a single 1181bp fragment consisting of fragments 1, E, and 2 and as predicted this composite enhancer fully recapitulates the temporal and spatial expression pattern of *eya* within the developing eye (Fig 3M–3O, Table 1). To rule out position dependent effects we inserted this construct into a second genomic landing site (*attP-9A VK00019—*cytological position 68D2) and observe that the expression pattern of this insertion is identical to the original insertion and recapitulates endogenous *eya* expression (S2 Fig). It appears that the temporal expression of the composite enhancer is the sum or addition of the individual elements. And interestingly, recreating the genomic organization of these three cis-regulatory elements eliminates the ectopic expression from the eye-antennal disc (Fig 3J–3O, Table 1). Since the composite enhancer recapitulates the entire *eya* expression pattern it is possible that fragments 3, 4, and PSE are functionally redundant. Consistent with this model, the expression patterns controlled by these fragments are fully covered by the composite enhancer (Fig 3P–3U, Table 1).

### The composite *eya* enhancer can fully rescue eye development in *eya*^*1*^ and *eya*^*2*^ mutants

**Fig 4:**
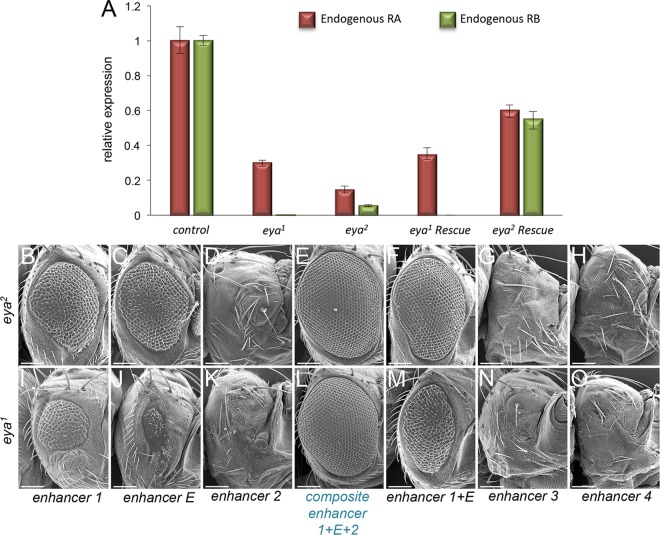
The composite enhancer fully restores eye development to *eya*^*1*^ and *eya*^*2*^ mutants. (A) qRT-PCR quantification of *eya* RA and RB transcript levels in eye-antennal discs. Y-axis measures the relative expression levels of each transcript. Raw data from single runs from three biological replicates were used to generate the graph. Error bars represent standard error. (B-O) SEM images of adult *Drosophila* compound eyes and heads from enhancer cDNA fusion rescue experiments. Each enhancer is driving expression of the *eya* RB isoform within the developing eye of *eya*^*1*^ and *eya*^*2*^ mutants. (B,I) *Enhancer 1—eya RB cDNA* fusion partially rescues 100% of animals examined. (C,J) *Enhancer E—eya RB cDNA* fusion partially rescues 100% of animals examined. Rescue efficiency is significantly reduced in *eya*^*1*^ background. (D,K) *Enhancer 2—eya RB cDNA* fusion does not rescue either *eya*^*1*^ or *eya*^*2*^ mutants. (E,L) *Composite enhancer—eya cDNA* fusion fully rescues 100% of animals examined to wild type eye size. (F,M) *Enhancer 1+E—eya cDNA* fusion partially rescues 100% of animals examined. (G,N) *Enhancer 3—eya cDNA* fusion does not rescue *eya*^*1*^ or *eya*^*2*^ mutants. (H,O) *Enhancer 4—eya cDNA* fusion does not rescue *eya*^*1*^ or *eya*^*2*^ mutants. Anterior is to the right in all adult head images. At least 100 adult flies were examined qualitatively for each genotype. Quantification of rescue (assayed by number of ommatidia) of a subset of adults is provided in Table 1. Scale bar, 100μm.

We then set out to test if the composite enhancer is sufficient to fully rescue the no-eye phenotypes of *eya*^*2*^ and *eya*^*1*^ mutants. The original characterization of the *eya*^*1*^ mutant indicated two chromosomal aberrations are associated with this mutation. First, a chromosomal re-arrangement completely inverts the orientation of the *eya* locus within the left arm of the second chromosome. This is not thought to interfere with normal *eya* expression. Second, an approximately 1.5kb deletion was detected at the 5`end of the gene. The 319bp deletion in *eya*^*2*^ lies within the larger ~1.5kb deletion in *eya*^*1*^. Thus the no-eye phenotype of *eya*^*1*^ and *eya*^*2*^ is thought to result from the disruption of the same regulatory sites [11,31]. To precisely determine the breakpoints of the *eya*^*1*^ deletion in relation to the composite enhancer we isolated and re-sequenced the region around the transcriptional start site and determined that the deletion is actually 1826bp in length with the deletion extending 581bp upstream of the *eya*^*2*^ deletion and 344bp downstream of the transcriptional start site. This deletion completely deletes the composite enhancer, the transcriptional start site, and a large portion of the *eya* RB transcript 5`UTR (Fig 2A). Using qRT-PCR we confirmed that the RB transcript is completely eliminated in *eya*^*1*^ mutants and drastically reduced in *eya*^*2*^ mutants (Fig 4A). The RA transcript is also greatly reduced, but not eliminated, in both mutant alleles suggesting that the composite enhancer regulates both *eya* promoters (Figs 2A and 4A).

To test whether fragments 1, E, and 2 are sufficient to rescue the two *eya* mutants, each enhancer element, as well as the full composite enhancer, was cloned upstream of a minimal hsp70 promoter and the *eya* RB cDNA. Using the PhiC31 integrase system these constructs were inserted into the same genomic location that we used for the original lacZ reporter expression analysis (*attP-3BVK00033—*cytological location 65B2). For all rescue experiments at least 100 adult flies were initially assayed qualitatively for the restoration of eye development. For the rescue quantification in Table 1 the number of ommatidia in adult right eyes from 2–3 individual female flies were counted and compared to wild type. The number of ommatidia per rescue is presented as an average of the 2–3 individuals. A wild type eye from a female fly is defined as having between 750 and 800 ommatidia [36].

Both fragments 1 and E are capable of partially restoring eye development in 100% of *eya*^*2*^ and *eya*^*1*^ mutants. Fragment 1 restores eye size to approximately 38% of wild type in *eya*^*2*^ and 25% in *eya*^*1*^ (Fig 4B and 4I, Table 1). Enhancer E restores eye size to approximately 49% of wild type in *eya*^*2*^ but less than 1% in *eya*^*1*^ (Fig 4C and 4J, Table 1). Expression from fragment 2, on its own, fails to rescue either mutant (Fig 4D and 4K, Table 1). Consistent with our expression analysis, the full composite enhancer fully restores eye development to 100% of both *eya* mutants (Fig 4E and 4L, Table 1). And finally, neither fragment 3 nor 4 are capable of rescuing the no-eye phenotype of either mutant (Fig 4G, 4H, 4N and 4O, Table 1). The majority of fragment 3 driven expression is outside of the endogenous *eya* expression pattern and would therefore not be predicted to restore eye development to *eya* mutants (Fig 3P–3R, Table 1). The inability of fragment 4 to rescue eye development stems from the fact that it is normally expressed only in differentiating cells posterior to the morphogenetic furrow (Fig 3S–3U, Table 1). Neither the furrow nor differentiated photoreceptor cells are present in either *eya*^*1*^ or *eya*^*2*^ mutants [11].

The lack of any discernable rescue by fragment 2 and its inappropriate expression pattern initially indicated that it may not function as an enhancer. Instead, its proximity to the transcriptional start site of *eya* RB, suggested that it might serve as a basal core promoter. To test this idea we placed fragment 2 and the composite enhancer into a plasmid that contains a lacZ reporter but lacks a minimal promoter. Under these conditions fragment 2 is still capable of driving lacZ expression in the developing eye but only in developing photoreceptors (S3A and S3B Fig). In contrast, lacZ reporter expression driven by the composite enhancer is identical to the construct that contained the minimal hsp70 promoter fragment (S3C and S3D Fig). These data support the proposal that fragment 2 functions, in part, as a basal promoter. As such we then tested the model that all pertinent regulatory information may reside only in fragments 1 and E. We first examined lacZ reporter expression with a fragment that contained segments 1 and E only and as expected this construct fully recapitulates endogenous *eya* expression (S3E and S3F Fig). We next attempted to rescue both *eya*^*1*^ and *eya*^*2*^ mutants with this shorter fragment. While we observed rescue in 100% of animals it only restores eye size to 82% of wild type in *eya*^*2*^ and 44% in *eya*^*1*^ mutants (Fig 4E, 4F, 4L and 4M, Table 1). This is unlike the composite enhancer, which completely restores eye size to the both *eya* mutants. This suggests that, in addition to functioning as a basal core promoter, fragment 2 does indeed contain regulatory information that is necessary for robust *eya* expression.

**Fig 5:**
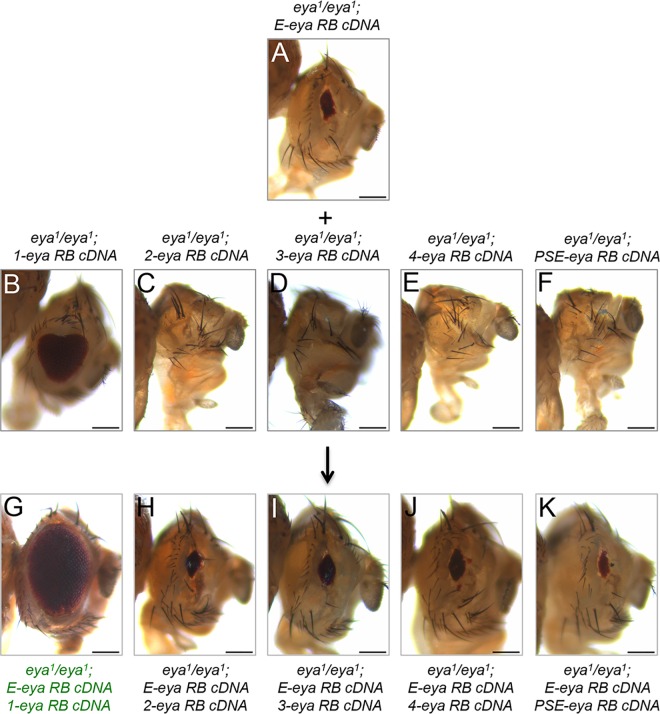
Cooperative interactions between enhancers 1 and E drive eye development. (A-K) Light microscope images of adult *Drosophila* compound eyes and heads from single and combination enhancer—*eya* RB cDNA fusion rescue experiments. Each enhancer is driving expression of the *eya* RB isoform within the developing eye of *eya*^*1*^ mutants. (A) *eya*^*1*^*/eya*^*1*^*; extant enhancer E—eya RB cDNA*. Expression of the *eya* RB cDNA driven by the extant enhancer alone weakly rescues 100% of animals examined. (B) *eya*^*1*^*/eya*^*1*^*; enhancer 1—eya RB cDNA*. Expression of the *eya* RB cDNA driven by enhancer 1 alone partially rescues 100% of animals examined. (C) *eya*^*1*^*/eya*^*1*^*; enhancer 2—eya RB cDNA*. Expression of the *eya* RB cDNA driven by enhancer 2 alone does not rescue the no-eye phenotype. (D) *eya*^*1*^*/eya*^*1*^*; enhancer 3—eya RB cDNA*. Expression of the *eya* RB cDNA driven by enhancer 3 alone does not rescue the no-eye phenotype. (E) *eya*^*1*^*/eya*^*1*^*; enhancer 4—eya RB cDNA*. Expression of the *eya* cDNA driven by enhancer 4 alone does not rescue the no-eye phenotype. (F) *eya*^*1*^*/eya*^*1*^*; enhancer PSE—eya RB cDNA*. Expression of the *eya* RB cDNA driven by the PSE enhancer alone does not rescue the no-eye phenotype. (G) *eya*^*1*^*/eya*^*1*^*; enhancer 1—eya RB cDNA/extant enhancer E—eya RB cDNA*. Combining *enhancer 1—eya RB cDNA* and *enhancer E—eya RB cDNA* constructs increases the quality of rescue as demonstrated by the larger eye size in 100% of animals examined. (H) *eya*^*1*^*/eya*^*1*^*; enhancer 2—eya RB cDNA/extant enhancer E—eya RB cDNA*. Combining *enhancer 2—eya RB cDNA* and *enhancer E—eya RB cDNA* constructs does not increase the quality of rescue over the extant enhancer alone. (I) *eya*^*1*^*/eya*^*1*^*; enhancer 3—eya RB cDNA/extant enhancer E—eya RB cDNA*. Combining *enhancer 3—eya RB cDNA* and *enhancer E—eya RB cDNA* constructs does not increase the quality of rescue over the extant enhancer alone. (J) *eya*^*1*^*/eya*^*1*^*; enhancer 4—eya RB cDNA/extant enhancer E—eya RB cDNA*. Combining *enhancer 4—eya RB cDNA* and *enhancer E—eya RB cDNA* constructs does not increase the quality of rescue over the extant enhancer alone. (K) *eya*^*1*^*/eya*^*1*^*; enhancer PSE—eya RB cDNA/extant enhancer E—eya RB cDNA*. Combining *enhancer PSE—eya RB cDNA* and *enhancer E—eya RB cDNA* constructs does not increase the quality of rescue over the extant enhancer alone. Anterior is to the right in adult head images. At least 100 adult flies were examined for each genotype. Scale bar, 100μm.

We were intrigued by the differences in rescue efficiency of our constructs in *eya*^*1*^ and *eya*^*2*^ mutants. Since the endogenous transcriptional start site for the RB transcript is intact in the *eya*^*2*^ mutant but is deleted in the *eya*^*1*^ mutant we hypothesized that the higher degree of rescue in the *eya*^*2*^ mutant is due to a reactivation of the endogenous *eya* gene. Using qRT-PCR we measured *eya* RB transcript levels within *eya* mutants that have been rescued by expression from enhancer E. This enhancer was chosen since it showed the most dramatic difference in rescue efficiency. As predicted, we observe that expression of the *eya* RB cDNA initiates a positive feedback loop on the endogenous locus and reactivates *eya* expression in *eya*^*2*^ but not *eya*^*1*^ (Fig 4A, 4C and 4J). In the *eya*^*2*^ mutant, fragments 1 (IAM), 2, 3, 4, and PSE are present and one or more of these could be targets of the auto-regulatory loop. To test this possibility we brought combinations of rescue constructs together within a single *eya*^*1*^ animal and asked if the degree of rescue could mimic that of the extant enhancer rescue of *eya*^*2*^. We combined the extant enhancer (E, Fig 5A) with each of the other enhancer elements (Fig 5B–5F) and observed a synergistic increase in the quality of rescue only with enhancer 1 (Fig 5G). The quality of eye restoration did not improve by combining the other elements with the extant enhancer (Fig 5H–5K). These data, when combined with the reporter expression and rescue results, suggest that enhancer 1 mediates the Eya-dependent auto-regulatory loop.

### The no-eye phenotype of *eya*^*2*^ mutants results from disrupting neighboring enhancers

**Fig 6:**
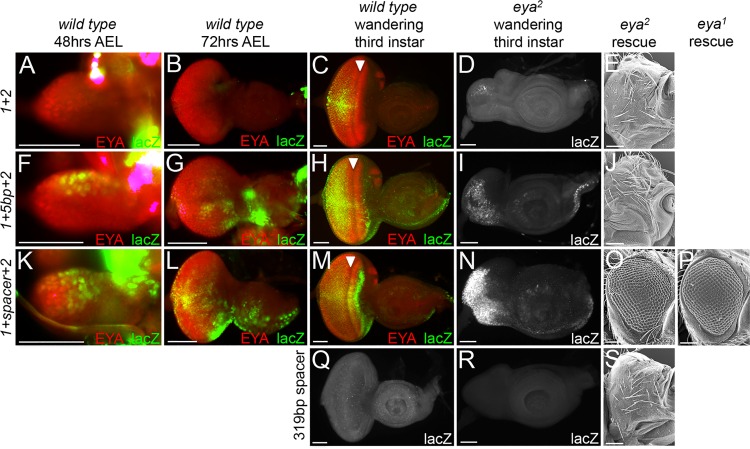
Spacing between enhancers is required for *eya* expression and function. (A-D, F-I, K-N,Q,R) Light microscope images of developing eye-antennal discs. Images of imaginal discs at 48hrs and 72hrs AEL were taken at 20X while images of wandering third instar larvae were taken at 10X. Red = Eya, green = β-galactosidase, yellow = regions where Eya and β-galactosidase co-localize. White arrowheads mark the position of the morphogenetic furrow. Each enhancer is driving expression of lacZ within wild type eye-antennal discs. AEL = after egg laying. (E,J,O,S) SEM images of adult *Drosophila* compound eyes and heads from enhancer—*eya* RB cDNA rescue experiments. Each enhancer is driving expression of the *eya* RB isoform within the developing eye of either *eya*^*1*^ or *eya*^*2*^ mutants. (P) SEM image of adult *Drosophila* compound eyes and heads from *enhancer 1+spacer+2—eya RB cDNA* rescue experiments of *eya*^*1*^ mutants. (A-C) Expression driven by enhancer 1+2 is only activated late in eye development in some *eya* expressing cells posterior to the furrow. (D) Expression driven by enhancer 1+2 is activated in very few cells in *eya*^*2*^ mutant discs. (E) The *enhancer 1+2—eya RB cDNA* does not rescue *eya*^*2*^ mutants. (F-H) Expression driven by enhancer 1+5bp+2 is activated mostly in non-*eya* expressing cells early in development while later activation is seen in *eya* expressing cells both anterior and posterior to the furrow. (I) Expression driven by enhancer 1+5bp+2 is activated weakly throughout the eye disc of *eya*^*2*^ mutants. (J) The *enhancer 1+5bp+2—eya RB cDNA* does not rescue *eya*^*2*^ mutants. (K-M) Expression driven by enhancer 1+spacer+2 restores some early expression in *eya* expressing cells but does not fully recapitulate *eya* expression at all stages of development. (N) Expression driven by enhancer 1+spacer+2 is strongly activated throughout the eye disc of *eya*^*2*^ mutants. (O) *Enhancer 1+spacer+2—eya RB cDNA* partially rescues 100% of *eya*^*2*^ mutants suggesting a restoration of function. (P) *Enhancer 1+spacer+2—eya RB cDNA* partially rescues 100% of *eya*^*1*^ mutants suggesting a restoration of function. (Q-R) The neutral 319bp of DNA that was used to construct 1+spacer+2 does not drive reporter activation on its own in either wild type or *eya*^*2*^ discs. (S) The 319bp spacer does not rescue *eya*^*2*^ mutants. Anterior is to the right in adult head and imaginal disc images. At least 100 adult flies and 30 imaginal discs were qualitatively examined for each genotype and at each developmental time point. Panel A-D, F-I, K-N, Q-R Scale bar, 50μm. Panel E,J, O,P,S Scale bar, 100μm

The ability of enhancer 1 to partially restore eye development to *eya*^*1*^ and *eya*^*2*^ mutants was of particular interest to us since eye development is completely blocked in the *eya*^*2*^ mutant despite the continued presence of enhancer 1. We hypothesized that the loss of eye development is due to the combined loss of enhancer E and a disruption of enhancer 1 activity. To test this model we recapitulated the genomic organization of the *eya*^*2*^ mutant by fusing enhancers 1 and 2 together. When the enhancers are placed in this configuration expression of the lacZ reporter is lost throughout young eye discs and ahead of the furrow in third instar discs. Expression of the reporter only remains in some differentiating cells posterior to the furrow (Fig 6A–6C, Table 1). Consistent with the loss of expression in undifferentiated cells, this construct drives reporter expression in a very small number of cells in the *eya*^*2*^ mutant (Fig 6D). The loss of expression in undifferentiated cells prevents this construct from rescuing the *eya*^*2*^ mutant (Fig 6E, Table 1).

The inability of this construct to properly drive lacZ and *eya* cDNA expression could be due to either the unintended creation of a synthetic binding site for a transcriptional repressor at the junction where enhancers 1 and 2 meet or there might be a need for some amount of genomic space between the two enhancers. To test the first possibility we placed a BamHI restriction site between enhancers #1 and #2. Addition of this 5bp spacer restores expression to some cells in wild type discs and to a few cells in *eya*^*2*^ mutant discs (Fig 6F–6I, Table 1). The expression pattern in wild type discs resembles that of enhancer 2 suggesting that insertion of the 5bps failed to allow for the early activation of enhancer 1. Consistent with this construct behaving similar to enhancer 2 we did not see any rescue of the *eya*^*2*^ mutant (Fig 6J, Table 1). Since this construct failed to restore *eya* expression and eye development, we can rule out the possibility that a synthetically created repressor site is the underlying reason for the loss of *eya* expression in *eya*^*2*^ mutants.

To test the latter hypothesis that a certain amount of genomic space is required between enhancers 1 and 2 we inserted a 319bp fragment of DNA (the size of enhancer E) between the two fragments in an effort to reinstate normal spacing. On its own this neutral sequence, which comes from intron 1 of the *eya* locus, does not direct expression of lacZ or rescue the *eya*^*2*^ mutant (Figs 2A and 6Q–6S, Table 1). At 48hrs and 72hrs AEL the majority, but not all, of the reporter expression of 1+spacer+2 was still seen in non-*eya* expressing cells (Fig 6K and 6L, Table 1). However, by the late third larval instar reporter expression is now seen in the majority of Eya positive cells (Fig 6M, Table 1). Overall early reporter expression of 1+spacer+2 is similar to that of enhancer 2 alone while late reporter expression is comparable to enhancer 1 alone (compare to Fig 3D–3F and 3J–3L, Table 1). This construct can drive expression in and partially rescue both *eya*^*1*^ and *eya*^*2*^ mutants demonstrating that the reconstitution of spacing was sufficient to restore limited function to enhancers 1 and 2 (Fig 6O and 6P, Table 1). The restoration of eye size in *eya*^*2*^ and *eya*^*1*^ is approximately 64% and 58% of wild type respectively, compared to 100% for the composite (1+E+2) enhancer, suggesting that in addition to providing critical space between enhancers #1 and #2, enhancer E must also contain regulatory information necessary for robust *eya* expression (Table 1).

### Activation of the composite enhancer is not So dependent

**Fig 7:**
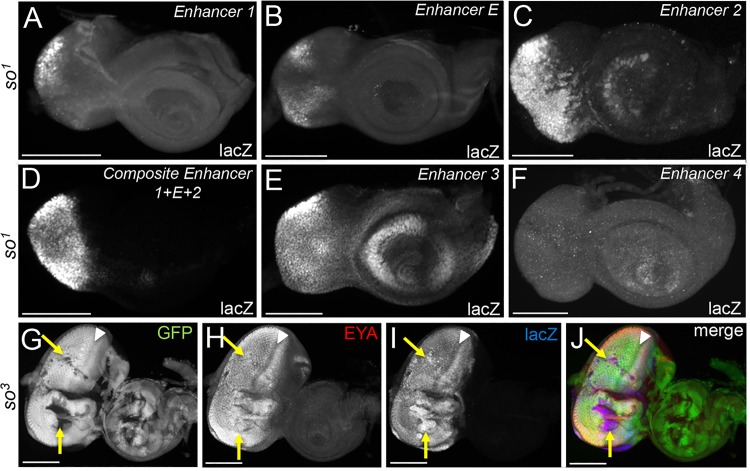
*eya* retinal enhancers remain transcriptionally active in *so* mutants. (A-F) Light microscope images of wandering third instar *so*^*1*^ mutant eye-antennal discs. Each enhancer is driving expression of lacZ within *so*^*1*^ eye-antennal discs. LacZ reporter activation is detected with an antibody that recognizes the β-galactosidase enzyme. (A) *so*^*1*^*; enhancer 1—lacZ*. Reporter expression driven by enhancer 1 is activated only in cells at the far posterior edge of the eye disc. (B) *so*^*1*^*; enhancer E–lacZ*. Reporter expression driven by the extant enhancer E is weakly activated throughout the eye disc. (C) *so*^*1*^*; enhancer 2 –lacZ*. Reporter expression driven by enhancer 2 is strongly activated throughout the remaining eye disc. (D) *so*^*1*^*; enhancer 1+E+2 –lacZ*. Reporter expression driven by the composite enhancer is strongly activated throughout the eye disc. (E) *so*^*1*^*; enhancer 3 –lacZ*. Reporter expression driven by enhancer 3 is activated broadly throughout the entire eye-antennal disc. (F) *so*^*1*^*; enhancer 4 –lacZ*. Reporter expression driven by enhancer 4 is not activated in the eye disc. (G-J) Light microscope images of wandering third instar eye antennal discs in which *so*^*3*^ null clones have been generated. The absence of GFP marks the position of clones lacking *so*. Green = GFP, red = Eya protein, blue = lacZ. Yellow arrows mark the position of *so*^*3*^ mutant clones in which Eya protein is present and the composite enhancer lacZ reporter is activated. White arrowheads mark the position of the morphogenetic furrow. Anterior is to the right in all imaginal disc images. At least 30 discs were examined for each genotype. Scale bar, 100μm.

Since we are able to partially restore *eya* expression and eye development to *eya*^*2*^ mutants through expression of the So-VP16 chimeric protein (Fig 1F and 1G) we reasoned that one or more of the newly discovered enhancers might be regulated by So. Three enhancers contain canonical So binding sites and So ChIP peaks are present within two other enhancers (Fig 2A) [33,34]. We first tested whether the So-VP16 chimeric protein is capable of activating the composite (1+E+2) enhancer. When forcibly expressed in the antennal disc under the control of the *dpp-GAL4* driver, So-VP16 is surprisingly unable to activate the composite enhancer (S4A–S4D Fig, rose arrow). In contrast, forced Ey does activate the reporter suggesting that Ey, but not So, regulates the *eya* locus during eye development (S4E–S4H Fig, yellow arrow). To further test whether activation of any of the enhancers is So dependent we placed each of the lacZ reporter constructs into the *so*^*1*^ mutant background and assayed for lacZ expression. *so*^*1*^ mutants are viable, lack compound eyes, have a small eye disc, and have drastically reduced levels of *so* expression (S5 Fig) [10,37]. Any So dependent element should remain silent in this mutant background. However, all of the elements with the exception of enhancer 4 remain activated in *so*^*1*^ mutant eye discs (Fig 7A–7F). Enhancer 4 drives expression exclusively in differentiating cells thus the lack of activation from this enhancer is most likely due to the fact that *so*^*1*^ mutants lack photoreceptor, cone, and pigment cells. It is striking that the composite enhancer remains strongly activated in *so*^*1*^ mutants (Fig 7D). To ensure that this is not due to residual So protein we examined lacZ expression driven by the composite enhancer in *so*^*3*^ null mutant clones. We again find that the composite enhancer is strongly activated in clones both ahead and behind the morphogenetic furrow (Fig 7G–7J). Thus, despite the presence of a So binding site and the apparent binding of So, the composite enhancer (which contains all regulatory information for proper *eya* expression) is not activated by So.

### So is not required for the initiation of *eya* expression but may be required for its maintenance

When we examined the potential activation of the composite enhancer in *so*^*3*^ null clones, we were quite surprised to see clones that contain Eya protein (Fig 7H). This clearly suggests that activation of reporters in *so* mutants is not due the persistence of lacZ protein although we cannot entirely rule that out. However, this result certainly was inconsistent with what we observed in late third instar whole mutant *so*^*1*^ discs where Eya protein was completely missing in the eye portion of the disc. These data were also inconsistent with qRT-PCR data, which showed a dramatic reduction of *eya* transcript levels in *so*^*1*^ mutants (S4 Fig). A possible explanation for these apparently contradictory observations could be that Eya protein expression is lost over the course of larval eye development. This could be the result of a requirement for So in the maintenance of *eya* expression, retinal progenitor cell death, a fate transformation, or a combination of all three [28,38]. Retinal progenitors have previously been defined as those proliferating in the most anterior regions of the eye disc and express Ey but lack So and Eya. Retinal precursors are defined as cells anterior to the morphogenetic furrow which express all three genes [39]. Support for the model that *eya* expression is lost over developmental time comes from three previously published observations: (1) *eya* expression is lost within the retinal field in roughly 50% of mid-late second larval instar *so*^*1*^ eye-antennal discs [29]; (2) *so*^*1*^ mutants undergo a significant wave of cell death that eliminates retinal progenitors in the growing eye field [10]; and (3) retinal progenitors within *so* and *eya* mutants undergo a fate transformation into head epidermis [28,38].

**Fig 8:**
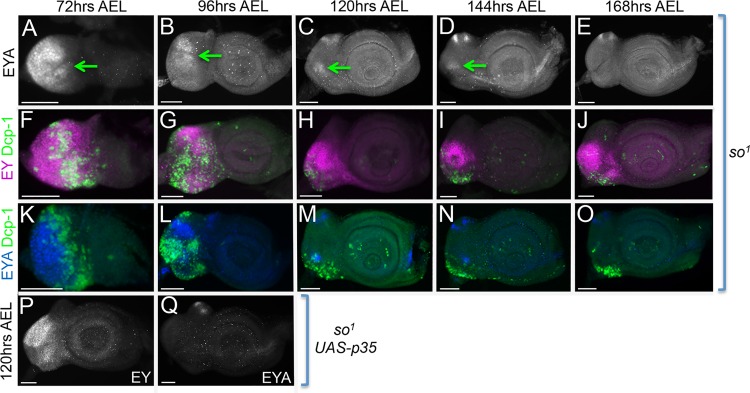
Eya expression is lost progressively in *so*^*1*^ mutants as a result of increased cell death in retinal progenitors. (A-O) Light microscope images of developing *so*^*1*^ mutant eye discs. (A-E) Green arrows indicate regions containing Eya protein. The amount of Eya protein is progressively lost in *so* mutant retinas. (F-O) Light microscope images of developing *so*^*1*^ mutant eye discs showing positions of dying cells. Cell death (green) is marked by Dcp-1, Ey (magenta) marks the position of progenitor cells, and Eya (blue) marks the position of precursor cells. Cell death in *so*^*1*^ mutants is elevated in both progenitor and precursor cells. (P-Q) Light microscope images of developing *so*^*1*^, *eya composite enhancer GAL4*, *UAS-p35*. Cell death has been blocked in the eye disc of *so*^*1*^ mutants by expression of the caspase inhibitor p35. Blocking death does not prevent the loss of *eya* expression in late third instar *so*^*1*^ mutant discs. AEL = after egg laying. Anterior is to the right in all imaginal disc images. At least 30 discs were examined for each genotype and at each developmental time point. Scale bar, 50μm.

To test our hypothesis that loss of Eya in *so*^*1*^ mutants is progressive we first re-examined *eya* expression in *so*^*1*^ mutants over the course of larval eye development. Beginning at 72hrs AEL we found that 100% of *so*^*1*^ mutant discs had strong Eya expression throughout the eye disc thereby demonstrating that So is not required for the initiation of *eya* expression (Fig 8A; S6 Fig). By 96hrs AEL *eya* expression weakens, is expressed in fewer discs, and is found in smaller and smaller populations of cells over time (Fig 8B–8D; S6 Fig). By 168hrs AEL the overwhelming majority of *so*^*1*^ discs have completely lost *eya* expression within the retinal field (Fig 8E; S6 Fig). This analysis confirms that Eya protein is indeed lost over the course of larval eye development.

To determine whether the loss of Eya protein could be due to increased cell death in retinal progenitors, as suggested by previous studies, we conducted a temporal examination of cell death in *so*^*1*^ mutants and find that retinal progenitors undergo significant cell death over the course of larval eye development (Fig 8F–8J). Using an antibody against Dcp-1, a marker of cell death, we observed increased cell death at 72hrs AEL in a large swathe of cells in the anterior most portions of the eye disc. At this point cell death seems restricted mostly to retinal progenitors outside the endogenous Eya expression domain as indicated by the expression of Ey but not Eya (Fig 8F and 8K). By 96hrs AEL the wave of cell death becomes broader and extends to the posterior margins of the disc to include both retinal progenitors and retinal precursors as indicated by the presence of both Ey and Eya expression (Fig 8G and 8L). It is important to note that 96hrs AEL is the first time point in which we see decreases in the expression of Eya protein. Finally, as development proceeds, the amount of cell death decreases and becomes restricted to the ventral most portions of the disc (Fig 8H–8J and 8M–8O). Although some Eya positive cells do appear to remain outside the population of dying cells it is clear that the majority of Eya expressing cells have been removed by these later time points consistent with the idea that retinal progenitors, and by default retinal precursors, have been cleared by cell death. These data also corroborate the qRT-PCR data from late *so*^*1*^ mutant discs.

If the loss of Eya expression in *so*^*1*^ mutants was solely the result of cell death of retinal progenitors then it follows that blocking cell death should restore *eya* expression to a subset of cells in late *so*^*1*^ mutant discs as those cells would be saved earlier in larval eye development and then proceed to differentiate into retinal precursors and express *eya*. To test this hypothesis we blocked cell death by expressing P35, a well-known inhibitor of caspase dependent cell death, with an *eya composite enhancer GAL4 driver*. We saw no increase in *eya* expression at 120hrs AEL suggesting that the loss of *eya* in *so*^*1*^ mutants is not simply the result of a clearing of retinal progenitors (Fig 8Q). However, we do see a significant number of cells still expressing *ey* indicating the continued presence of retinal progenitors that are not proceeding to differentiate into retinal precursors (Fig 8P).

**Fig 9:**
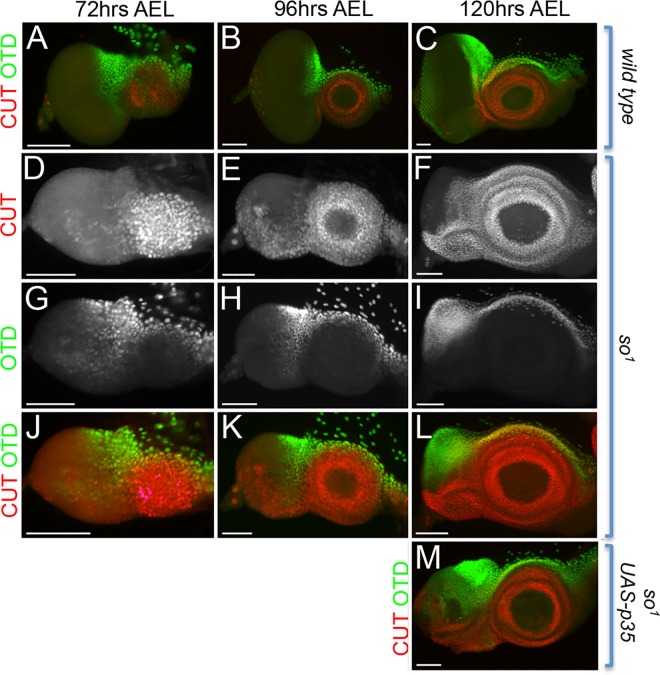
Retinal progenitors within *so*^*1*^ mutants progressively transform into head epidermis. (A-M) Light microscope images of developing wild type, *so*^*1*^, and *so*^*1*^, *eya composite enhancer GAL4*, *UAS-p35* eye-antennal discs. Green = Otd and red = Cut. Expression of both *otd* and *cut* is de-repressed within the eye field of *so*^*1*^ mutant discs over the course of larval eye development. Although cell death has been blocked in the eye disc of *so*^*1*^ mutants by the caspase inhibitor p35, expression of *otd* and *cut* is still de-repressed in retinal progenitors. AEL = after egg laying. Anterior is to the right in all disc images. At least 30 discs were examined for each genotype at each developmental time point. Scale bar, 50μm.

We have previously shown that retinal progenitors within *so* and *eya* mutants undergo a cell fate transformation into head epidermis [28,38]. It is possible that after the wave of cell death the continued loss of *eya* expression in *so*^*1*^ mutants may be the indirect result of this homeotic transformation. The non-ocular bristle and antennal selector gene *cut* (*ct*) and the head capsule selector gene *orthodenticle* (*otd*) have previously been shown to be de-repressed in eye to head epidermis transformations in *eya*^*2*^ mutants [28]. We therefore examined expression of both genes in *so*^*1*^ mutants. Concomitant with the decrease in *eya* expression, we saw a de-repression and expansion of both *ct* and *otd* throughout the entire eye disc (Fig 9A–9L). The de-repression of both genes initiates at 72hrs AEL in just a few cells of the eye disc but is more pronounced by 96hrs AEL (Fig 9D, 9E, 9G, 9H, 9J and 9K). Most striking is that starting at 120hrs AEL, when we first begin to see discs without any *eya* expression in the retinal field, *ct* and *otd* expression have expanded to cover the entire eye field (Fig 9F, 9I and 9L). Based on the continued presence of *ey* expression within the same portion of the eye disc it appears that the surviving retinal progenitor cells have undergone a transformation to head epidermis (Fig 8H). Furthermore, when we block cell death in the mutants, *ct* and *otd* are still expressed in the majority of cells within the disc again supporting the idea that these cells have undergone a cell fate transformation (Fig 9M). We believe it is this ongoing cell fate transformation that is blocking the continued expression of *eya* resulting in a loss of Eya protein in late stage *so*^*1*^ mutant discs. However, additional studies are needed to fully determine if, and possibly to what extent, So might be required for the maintenance of *eya* expression later in larval eye development.

**Fig 10:**
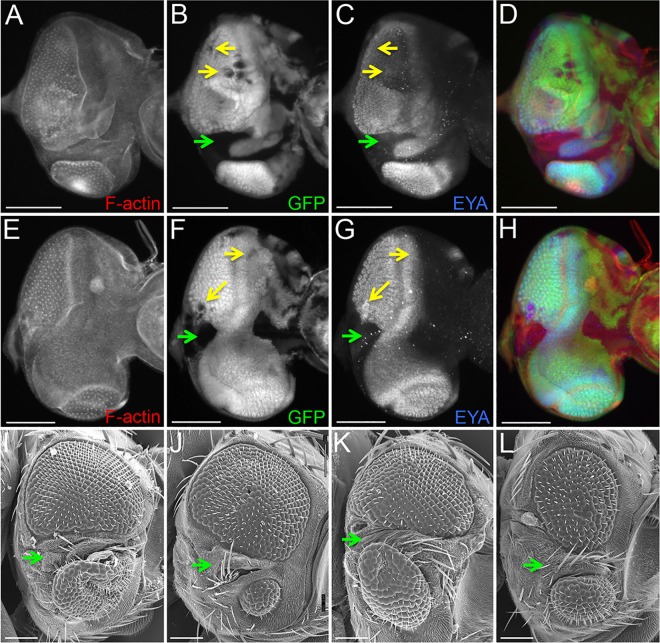
Eya protein is detected in non-transformed *so*^*3*^ mutant clones. (A-L) Eye discs and adult heads of the genotype: *eyflp; FRT42D so*^*3*^ */FRT42D Ubi-GFP* that contain *so*^*3*^ mutant clones. (A-H) Light microscope images of developing eye-antennal discs. Yellow arrows identify *so*^*3*^ clones that have not transformed into head epidermis and still express *eya*. Green arrows demarcate large *so*^*3*^ clones that are transforming into head epidermis and lack Eya protein. Red = F-actin, green = GFP, blue = Eya. (I-L) SEM images of adult *Drosophila* compound eyes and heads. Green arrows indicate regions of the compound eye that has transformed into head epidermis. Green arrows in panels I-L indicated regions of head epidermis bifurcating the retinal field. Anterior is to the right in all adult head and imaginal disc images. At least 30 adult eyes and imaginal discs containing *so*^*3*^ clones were examined. Scale bar, 100μm.

Finally, to ensure that the Eya protein we observed in *so*^*1*^ mutants is not the result of residual levels of So protein activity we examined *eya* expression in *so*^*3*^ null mutant clones. Consistent with the analysis of *so*^*1*^ discs, we found multiple *so*^*3*^ null clones in which Eya protein was still present (Fig 10A–10H, yellow arrows). We did, however, observe that the majority of large clones spanning the middle of the eye field contained no Eya protein (Fig 10A–10H, green arrows). The adult retinas of these animals often contain large patches of head epidermis protruding through the middle of the eye field (Fig 10I–10L, green arrows). We predict that these patches of head cuticle correspond to the clones in the disc that lack *eya* expression and thus are the result of a cell fate transformation.

Together our lacZ reporter and cDNA rescue analyses suggest that a single 1181bp genomic fragment composed of three cis-regulatory elements is capable of controlling all *eya* expression in the developing retina. Furthermore, our combinatorial rescue analysis in *eya*^*1*^ mutants suggests that although enhancer elements 1 and E sit adjacent to each other within the *eya* locus, these two elements are functioning as independent cis-regulatory elements. Additionally, we find that spacing between elements within the composite enhancer is critical for proper function. When fragments 1 and 2 are located adjacent to each other as is the case in *eya*^*2*^ mutant animals these enhancers can no longer function to provide *eya* expression early in larval development leading to an adult no-eye phenotype. Finally, we find that loss of *eya* expression in *so* loss-of-function mutants is progressive and likely the result of increased cell death and a cell fate transformation. Although our data cannot rule out the possibility that So is required for the maintenance of *eya* expression during larval eye development it is clearly not required for its initiation. Given our identification of multiple independently functioning cis-regulatory elements within the *eya* locus and the potential differential requirement for So in its activation at later stages of eye development, *eya* regulation over the course of eye development is likely to be dynamic and require the input of different combinations of RD members and signaling pathways at different times and in different cell types for overall proper temporal and spatial expression.

## Discussion

**Fig 11:**
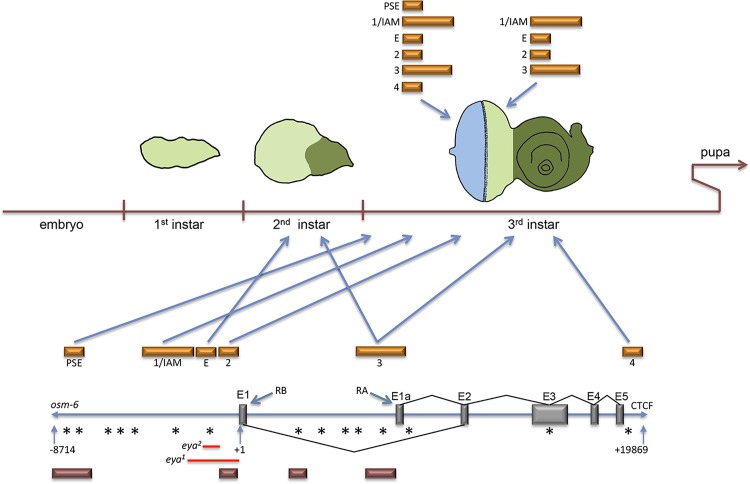
Summary of cis-regulatory control of the eya locus in the developing eye. This summary diagram depicts spatial and temporal control that cis-regulatory elements exert on the expression of *eya* in the developing retina. Orange bars = *eya* cis-regulatory elements, red bars = So ChIP binding peaks, red lines = regions deleted in *eya*^*1*^ and *eya*^*2*^ mutants, grey bars = exonic sequences, asterisks = position of So consensus binding sites, and RA/RB refer to two predicted isoforms of Eya. The osm-6 gene and a CTSF insulator site were used to define the *eya* locus. Eya expression is initiated during the second instar stage with enhancers E and 3 driving expression in undifferentiated cells (light green) that are normally positive for Eya protein. At the onset of the third larval instar, the morphogenetic furrow initiates and begins the process of converting the undifferentiated cells into an ordered array (light blue) of differentiating photoreceptors, cone, and pigment cells. At this stage all six enhancers are expressed in differentiating cells. However, only four of the enhancers drive expression in the undifferentiated cells. During development the antennal disc (dark green) does not show Eya protein. However, the individual enhancers can drive ectopic expression in this zone (see Fig 3). The spurious expression in the antenna is eliminated when enhancers 1,E, and 2 are fused together (composite enhancer) to mimic their organization in the genome (see Fig 3). Anterior is the right.

Members of the retinal determination network play crucial roles in specification, pattern formation, cell fate choice and proliferation during *Drosophila* compound eye development. As such their regulation and gene expression is often highly temporally and spatially dynamic allowing for the proper differentiation of the multiple cell types necessary for the proper function of the compound eye [40]. *eyes absent* is a core member of this network and provides a key example of this type of complex gene expression. In this report we have identified several enhancers that cooperate to regulate temporal and spatial expression of *eya* in the developing retina (Figs 2 and 11). It is not uncommon for a single expression pattern to be controlled by multiple enhancers [39–44]. We find that a single enhancer module, comprised of three distinct and separable *cis-regulatory* elements, is responsible for the correct temporal and spatial expression of *eya* (Figs 3 and 4). Furthermore, the three elements (1+E+2) that comprise the composite enhancer regulate *eya* at specific times during retinal development (Figs 3 and 11). For example, enhancer E controls early *eya* expression while enhancer 1 is responsible for the bulk of late *eya* transcription (Fig 3). Having separate *cis-regulatory* elements control *eya* expression at different times during development is consistent with the idea that RD genes are dynamically regulated temporally and spatially to insure distinct expression patterns necessary for the differentiation of specific retinal cell types over the course of eye development [40].

In the context of temporal expression we propose that these enhancers function additively (Figs 3,5 and 11). Additive control of gene expression levels has been described in many organisms including *Drosophila*. In the *Drosophila* embryo a set of proximal and distal enhancers controls the expression patterns of the *hunchback* (*hb*) and *knirps* (*kni*) gap genes [41–44]. The expression level of each of these two genes appears to be the sum of the levels that are driven by the individual enhancers [45]. Although additional experimentation to measure the specific contribution of each of the elements in terms of transcriptional output of *eya* is required, the results of our cDNA fusion rescue experiments support a model for these elements functioning additively as the overall size of the eye increases when enhancers #1 and E are combined and complete rescue occurs when all three elements (#1+E+2) are placed together (Figs 4 and 5).

It has been shown that deleting enhancer E leads to a loss of *eya* expression early in development when it is normally needed to promote tissue specification and cell proliferation. We find that this early loss of *eya* expression is a major, but not the sole, contributor to the complete loss of the compound eye in *eya*^*2*^ mutants. Our results indicate that spacing is critical for the proper function of two cis-regulatory elements within the composite enhancer (Figs 4 and 6). Specifically, we find that the primary cause for the loss of *eya* expression in *eya*^*2*^ mutants is the direct juxtaposition of two flanking cis-regulatory elements (1 and 2) rather than the deletion of the intervening regulatory element (E). Placement of a neutral sequence between these two elements recapitulates normal spacing in the genome, restores their ability to drive a transcriptional reporter, and rescues the no-eye phenotype of *eya*^*2*^ mutants (Fig 6). The *eve* locus provides a parallel example to what we observe in the eye with *eya*. The *eve* stripe 2 and stripe 3 enhancers are separated by 1.7kb of neutral genomic sequence. When these enhancers are placed directly adjacent to each other the expression pattern driven by both enhancers is altered. Normal expression is restored when a short 160bp sequence is inserted between the two enhancers suggesting that without correct spacing improper short-range interactions between cis-regulatory elements can lead to abnormal expression patterns [46]. In the *eya*^*2*^ mutant, enhancers 1 and 2 are directly juxtaposed to each other. Since expression of *eya* is lost in all undifferentiated cells we propose that inappropriate short-range repression between the two enhancers is likely inactivating both elements. We have not investigated the minimal spacing requirements for the *eya* retinal enhancers but based on the results from [46], the distance is likely to be relatively short.

It has been widely assumed that So plays a role in regulating *eya* in cells undergoing eye specification. This was based in part on the loss of *eya* expression in *so* loss-of-function mutants [29] as well as the presence of So binding sites within the *eya* locus (including enhancer E) and the ability of So to bind to the *eya* locus [11,33,34]. In fact it is the presence of predicted So binding sites which first led us to explore which DNA elements are controlling *eya* expression in the developing retina. When placed within *so* mutant backgrounds we found, however, that the *eya* enhancers were still active (Fig 7). This led us to re-examine Eya protein expression in *so* loss-of-function mutants and we find that Eya expression is lost progressively over the course of larval eye development (Fig 8). Although these results clearly demonstrate that So is not required for the initiation of *eya* expression they do not rule out a role for So in the maintenance of its expression.

The progressive loss of *eya* could be the consequence of a requirement for So in the regulation of *eya* later in larval eye development. Our initial analyses of the *eya* enhancers would partially support this model of regulation. Enhancer 2, which is bound by So, functions within photoreceptors late in eye development and therefore would be a promising candidate for regulation by So (Figs 3 and 11). Conversely, enhancer E, which contains a predicted So binding site, is the cis-regulatory element responsible for the bulk of early not late *eya* expression (Figs 3 and 11). It seems unlikely that So is regulating *eya* through this enhancer given that So is not required for the initiation of *eya* expression. Two additional enhancers (PSE and enhancer 4) which we find to function redundantly to the composite enhancer both contain predicted So binding sites and a larger DNA fragment containing the PSE was found to be bound by So. Like enhancer 2, both of these enhancers function in photoreceptors and cone cells later in eye development and as such might be good candidates for the maintenance of *eya* by So (Figs 3 and 11). In our assays these elements seem to function redundantly to the composite enhancer therefore it is likely if these enhancers are regulated by So it is in a manner more similar to that of a shadow enhancer [44] to ensure robust *eya* expression. Interestingly, bioinformatic conservation analysis on these enhancer elements would suggest that a requirement for So in the regulation of e*ya* might not be conserved across *Drosophila* species. Analysis of the composite enhancer shows that the bulk of conservation lies only within enhancers 1 and E (S7A Fig). There is no sequence conservation within enhancer 2 and the So binding sites in enhancers E, PSE, and 4 are also not conserved (S7A-S7C Fig). However, a stretch of sequence conservation in the PSE is present immediately adjacent to the predicted So binding site (S7B Fig).

Previous studies on *so*^*1*^ and *eya*^*2*^ mutants support an alternative model in which the loss of *eya* in *so* mutants could be the result of a combination of increased cell death of retinal progenitors [17,18] and a progressive cell fate transformation from retinal progenitor to head epidermis [35,38]. And indeed we observe both phenomena occurring simultaneously in *so* loss-of-function mutants (Figs 8–10). Our re-examination of the *so* mutants showed *eya* expression slowly terminates as the tissue is gradually altering its fate (Fig 8). The state of the cell and/or tissue is an underappreciated idea that needs to be considered when attempting to establish regulatory relationships between transcription factors and putative downstream targets. A wealth of expression data and evidence of molecular interactions may not be sufficient, in all cases, to conclude that a gene is under the control of the DNA binding protein in question.

## Materials and Methods

### Fly Strains

The following fly strains were used in this study: (1) *eya*^*1*^, (2) *eya*^*2*^, (3) *so*^*1*^, (4) *FRT42D so*^*3*^*/CyO*, (5) *FRT42D Ubi-GFP/CyO*, (6) *y w eyflp*, (7) *w*^*1118*^, (8) *w;; ey-GAL4*, (9) *UAS-so*^*VP16*^, (10) *y*^*1*^ M(vas-int.Dm))[2]ZH-2A w^*^; PBac(y^*+*^*-attP-3B)VK00033—BL24871*, (11) *y*^*1*^ *M(vas-int*.Dm)ZH-2A w^*^; PBac(y^*+*^*-attP-9A)VK00019*—*BL24866*, (12) *so*^*1*^, *UAS-P35*, (13) *w*^*1118*^*; eya composite enhancer-GAL4*. Loss-of-function clones were generated with the following genotype: *y w eyflp; FRT42D so*^*3*^*/FRT42D Ubi-GFP*. All crosses were conducted at 25°C. BL = Bloomington Drosophila Stock Center

### Antibodies, Dissections, and Microscopy

The following antibodies were used: (1) mouse anti-Eya (1:5, DSHB), (2) mouse anti-β-galactosidase (1:250, Promega), (3) chicken anti-β-galactosidase (1:800, Promega), (4) rat anti-Elav (1:100, DSHB). (5) rabbit anti-Dcp-1 (1:100, Cell Signaling Technologies). DSHB = Developmental Studies Hybridoma Bank. Fluorophore-conjugated secondary antibodies and phalloidin-fluorophore conjugates were obtained from Jackson Immuno Research Laboratories and Life Technologies. Imaginal discs were prepared as described previously in [47]. For dissections performed at specific time intervals, adult flies were placed in egg laying chambers and allowed to lay for 30–60 minutes on agar plates. Individual embryos were then transferred to individual microcentrifuge tubes with approximately 200ul of standard fly media. The tubes were then placed at 25°C and aged for the appropriate amount of time. Eye-antennal discs were photographed on a Zeiss Axioplan II compound microscope. For scanning electron microscopy, adult flies were serially incubated in 25% ethanol, 50% ethanol, 75% ethanol, 100% ethanol, 50% ethanol: 50% hexamethyldisilazane (HMDS), and then 100% HMDS, coated with gold-palladium, and viewed with a JEOL 5800LV SEM. For light microscopy of adult heads, flies were photographed on a Zeiss Discovery Microscope.

### Luciferase Reporter Activation Assays

3mL of *Drosophila* Kc167 cells (approximately 1X10^7^ cells/mL) were transfected with a total of 400ng of plasmid DNA using the Qiagen Effectene Transfection Reagent (Cat. No. 301427). For each transfection, mt-GAL4 (136ng) was transfected along with the indicated UAS responder plasmids (64ng each) and ARE-luciferase (132ng). UAS-renilla (0.26ng) was also included in the transfection mix as a control for transfection efficiency. The plasmids were diluted in 98μL of Buffer EC, then 3.2μL of the Enhancer Solution was added to the dilution. The solution was incubated at room temperature for 5min. 10μL of Effectene Transfection Reagent was added to the dilution and the solution was incubated at room temperature for an additional 10min. The transfection solution was mixed with 600μL of Hyclone SFX Insect Culture Media (Cat. No. SH30278.02) and added drop-wise to the plated cells.

Following transfection the cells were incubated at 25°C for 20hr. Protein production was then induced by the addition of 1mM CuSO_4_. Following induction cells were incubated at 25°C for an additional 24hr before harvesting for determination of luciferase activity. The luciferase activity was assayed using the Promega Dual Luciferase Reporter Assay System (Cat. No. E1910) and a Promega GloMAX 20/20 Luminometer (Model No. E5311). Cells were collected by centrifugation at 500g for 2min. The supernatant was removed and the pellet was re-suspended in 500μL of Passive Lysis Buffer (PLB) at the working concentration. The cells were lysed in the PLB for 20min at room temperature.

20μL of cell lysis solution was added to 100μL of Luciferase Assay Reagent II and mixed by pipetting for 10sec. The light output of the solution was measured in the luminometer once a second for 10s and the average output over the time period was recorded. This was the activity of the luciferase enzyme—the experimental result. 100μL of Stop and Glo Reagent was then added to the tube and mixed briefly by vortexing. The light output of the solution was once again measured, and the results were recorded as the output from Renilla enzyme—the transfection efficiency control. These two measurements were performed for each of three separate plates of independently transfected cells for each plasmid combination. The Relative Luciferase Units (RLUs) for each combination of plasmids were calculated by dividing the experimental light output (Luciferase) by the transfection efficiency control (Renilla) for each of the three independent transfections. Error bars in Fig 1 represent standard deviation

The target sequence for So and So-VP16 consists of five copies of the ARE element (GGT GTC AGG TTG CTC GAG) that is reported in [23,48] placed upstream of the luciferase gene within the pGL3 vector (Promega, catalog #E1751).

### LacZ transcriptional reporters

For lacZ reporter analysis individual genomic fragments illustrated in Fig 2 were amplified from *w*^*1118*^ genomic DNA and cloned into either p-lacZ.attB or pg-lacZ.attB plasmids (Konrad Basler, University of Zurich, Switzerland). Genomic fragment sequences are provided in S1 Table. Cloning strategies and primer sequences are listed in S2 Table. RED refers to standard restriction enzyme digestion and ligation into a multiple cloning site. Gateway refers to the Life Technologies Gateway Recombination Cloning system.

### *eya* RB cDNA rescue plasmid

For the cDNA enhancer fusion rescue assay, a pg-*eya* RB+3’UTR cDNA.attB plasmid was created by modifying an existing pg-RFP.attB plasmid (derived from pg-lacZ.attB). The *eya* RB+3’UTR cDNA was first amplified by PCR from an existing pUAS-*eya* RB+3’UTR plasmid as an EcoRI-NdeI fragment and cloned into a pg-RFP.attB plasmid. Portions of the Gateway cloning cassette and hsp70 minimal promoter were then amplified from pg-RFP.attB as an EcoRI fragment and cloned ahead of the *eya*RB+3’UTR cDNA. Primer sequences are listed in S2 Table.

### Enhancer *eya* cDNA fusion rescue constructs

Putative enhancers (Fig 2A) were amplified from the appropriate p.lacZ.attB plasmid and cloned into the new pg-*eya* RB+3’UTR cDNA.attB plasmid using Gateway recombination cloning (Life Technologies). Gateway 5’ att primer sequence: 5`-GGG GAC AAG TTT GTA CAA AAA AGC AGG CTC AAC-3`and Gateway 3’ att primer sequence: 5`-GGG GAC CAC TTT GTA CAA GAA AGC TGG GTC CTA-3`

### Integrated DNA Technologies (IDT) synthesized constructs

Enhancer 2 minimal fragment and enhancer 1+5bp+2 fragment were synthesized by IDT and flanked by Gateway att sequences for recombination into the pg-lacZ.attB and pg-*eya* RB+3’UTR cDNA plasmids.

### Enhancer 1+2 lacZ transcriptional reporter and cDNA fusion constructs

Enhancer 1 was amplified from the p-eya-enhancer 1.lacZ.attB plasmid with the following primers: 5`primer: 5`-ATA ATA AAG CTT ACT ACA CCT CGT ACC AAA TTC TCG G-3`and 3`primer: 5`-CCT GCT CAA CTC AAA TGG CCA GTT TCG TCT CC-3`Enhancer 2 was amplified from the p-eya enhancer 3.lacZ.attB plasmid using the following primers: 5’ primer: 5`-GGA GAC GAA ACT GGC CAT TTG AGT TGA GCA GG-3`and 3’ primer: 5`-ATA ATA GGT ACC TCA ACT GAT TCG ACT TGG TCG-3`PCR products were combined together using Gibson Assembly (New England Biolabs). Gateway recombination sequences were then added to the 5`and 3`ends of the product using the following primers: 5`primer: 5`-GGG GAC AAG TTT GTA CAA AAA AGC AGG CTC AAC ACT ACA CCT CGT ACC AAA TTC TCG G-3`and 3`primer: 5`-GGG GAC CAC TTT GTA CAA GAA AGC TGG GTC CTA TCA ACT GAT TCG ACT TGG TCG AAA AGC-3`The resulting fragment (enhancer 1+2) was cloned into the pDONR201 plasmid and shuttled into pg-lacZ.attB and pg-*eya*RB+3’UTR cDNA.attB plasmids using Gateway recombination cloning (Life Technologies).

### Enhancer 1+spacer+2 lacZ transcriptional reporter

Enhancer 1 minimal fragment was amplified from the pg-eya enhancer 1 minimal.lacZ.attB plasmid using the following primers: 5`primer: 5`-AAA TAT TTG GAT ATG TGG GGG AAA GGG-3`and 3’ primer: 5`-ATA ATA GAA TTC GGC CAG TTT CGT CTC CTC TTT TGC-3`(adds an EcoRI site). The spacer fragment was amplified from the pg-eya intron 1-1.lacZ.attB plasmid using the following primers: 5`primer: 5`-ATA ATA GAA TTC TGA AAG ATC TCA ATT AGC TAA CCG-3`(adds an EcoRI site) and 3’ primer: 5`-ATA ATA TCT AGA CAA CTG CTA CCA TTT TGG CCA TTT C-3`(adds a XbaI site). Enhancer 2 was amplified from the p-eya enhancer #2.lacZ.attB plasmid using the following primers: 5’ primer: 5`-ATA ATA TCT AGA ATT TGA GTT GAGCAGGTCAGTTAATATTAC-3`(adds a XbaI site) and 3’ primer: 5`-TCA ACT GAT TCG ACT TGG TCG-3`The three fragments were ligated together to generate 1+spacer+2. The following primers were then used to amplify this product, which was then cloned into the p-lacZ.attB plasmid as a HindIII-KpnI fragment. 5’ primer: 5`-ATA ATA AAG CTT AAA TAT TTG GAT ATG TGG GGG AAA GGG-3`(adds a HindIII site) and 3’ primer: 5`-ATA ATA GGT ACC TCA ACT GAT TCG ACT TGG TCG-3`(adds a KpnI site).

### Enhancer 1+spacer+2 *eya* RB cDNA fusion

The pg-*eya* RB cDNA+3’UTR.attB plasmid (see above) was digested with HindIII and KpnI resulting in a plasmid missing the Gateway cassette, hsp70 promoter and a portion of the *eya* RB cDNA. Into this plasmid was cloned the 1+spacer+2 region (see above) as a HindIII-KpnI fragment resulting in a p-eya 1+spacer+2 *eya* RB cDNA+3’UTR(partial).attB plasmid that is still missing the hsp70 minimal promoter and a portion of the *eya* RB cDNA. These pieces were amplified as a single fragment from pg-*eya* RB cDNA+3’UTR.attB using the following primers: 5’ primer: 5`-TCG AAT CAG TTG AGG TAC CTC TAG AGC-3`(adds a KpnI site) and 3’ primer: 5`-CCA GAG CCG GCG GTA CCC ACA CTG-3`(adds a KpnI site). This fragment was cloned into p-eya 1+spacer+2 eyaRB cDNA+3’UTR (partial).attB as a KpnI fragment to yield the final p-eya 1+spacer+2 eyaRB cDNA+3’ UTR.attB plasmid.

### Cloning of enhancers into the promoterless pg-lacZ.attB vector

Enhancer 2 and the composite enhancer were amplified from the p-eya enhancer 2.lacZ.attB and p-eya composite enhancer.lacZ.attB plasmids respectively (primer sequences are listed in S2 Table). The 3’ primer adds 40bp of genomic sequence downstream of enhancer 2 and the transcriptional start site to ensure the entire endogenous promoter region was included. These 40bp were omitted from the above plasmids since a hsp70 minimal promoter is included within the plasmid. Gateway recombination sequences were added to the ends of each construct and the fragments were cloned into the pg-lacZ.attB plasmid that lacks a hsp70 promoter (Konrad Basler, University of Zurich, Switzerland) using standard Gateway Recombination Cloning.

### Generation of transgenic fly strains

All lacZ reporter and cDNA fusion constructs were stably integrated into the *pBAC(y+-attP-3B)VK00033* third chromosome landing site using PhiC31-mediated integration. Proper site-specific integration was confirmed by PCR with attP/attB primers and the correct sequence of the construct was confirmed. The composite enhancer-lacZ construct was also inserted into a second landing site on the third chromosome for comparison: *PBac(y*^*+*^*-attP-9A)VK00019*.

### Molecular analysis of the *eya*^*1*^ deletion

The genomic region surrounding the *eya*^*1*^ deletion [31] was amplified from genomic DNA of the *eya*^*1*^ stock (BL-3631). Genomic DNA from the same region was amplified from *w*^*1118*^. The following primers were used to amplify the area surrounding the deletion: 5`primer: 5’-TTC CCG CTG GTG ACT TAC TG-3’ and 3`primer: 5’-GTT GTG AGG GAG CTG TCT GG-3’ The 5’ primer sits 2683 bp upstream of the *eya* RB transcriptional start site and the 3’ primer sits 702bp into the first intron. Q5 high-fidelity DNA polymerase (New England Biolabs) was used for the amplification. The PCR product was purified using GeneJet PCR Purification Kit (Thermo-Fisher #K0701). The amplified region from *w*^*1118*^ is approximately 4kb while it is just over 2kb in the *eya*^*1*^ mutant stock. Twelve sequencing primers were used to sequence the amplified genomic region in both directions. Primer sequences are listed within S3 Table. The *eya*^*1*^ deletion is 1826bp in size: it begins 581bp upstream of the 5’ start of the *eya*^*2*^ deletion and extends 344bp into the 5’ UTR of the eyaRB transcript. There are an additional 11bp that do not correspond to the published genomic region. BL = Bloomington Drosophila Stock Center

### qPCR

qPCR was performed as previously described [49]. For each experiment three biological replicates were analyzed once. For each biological replicate, approximately 50 eye-antennal imaginal discs from wandering 3^rd^ instar larvae were dissected in PBS and immediately placed into a microcentrifuge tube containing 200ul of RLT buffer with β-mercaptoethanol (Qiagen #79216). The tissue was disrupted with a pestel for 1 minute. After disruption an additional 150ul of RLT buffer with β-mercaptoethanol was added to the tube and the sample homogenized using a QIAshredder column (Qiagen #79654). After homogenization total RNA was isolated using the Qiagen RNeasy Mini Kit (Qiagen #74101). 100-200ng of total RNA was reverse transcribed to cDNA using the SuperScript III First Strand Synthesis System with oligo(dT) primers (Invitrogen). qPCR was performed on a Roche LightCycler 480 using SYBR Green I Master Mix (Roche). For each experiment, target genes were analyzed on biological triplicate samples and normalized to *rp49*. 3–4 serial dilutions of pooled cDNA were used to determine primer amplification efficiencies for each target gene. In *eya*^*1*^ and *eya*^*2*^ rescue experiments, primers specific to the endogenous *eya* RA and *eya* RB transcripts were used. Roche LightCycler 480 Software (Version 1.5) was used to calculate cycle threshold values and melting curves for each reaction. Relative expression and standard error was calculated using Relative Expression Software Tool (REST) [50]. Error bars generated by REST analysis reflect standard error determined by a confidence interval centered on the median, allowing representation of asymmetric tendencies in the data. Primers were designed using A plasmid Editor (ApE) or Fly Primer Bank [51]. Primer sequences are listed in S4 Table.

### So binding site sequence analysis

Examination of the *eya* locus (both strands) for predicted So binding sties was performed using the following reported sequences: GTAANYNGANAYC [52], GTAANYNGANAYG [52], GGTATCA [53], GATATCA [53], TGATAC [54], TGATAC [32], CGATAC [32], ATTGATATCAAT [55], and TTGATATCAA [55].

## Supporting Information

S1 Fig Expression of So-VP16 (but not So) rescues the *eya*^*2*^ mutant. (A-C) SEM images of adult *Drosophila* compound eyes and heads from *eya*^*2*^*; ey-GAL4*, *UAS-So-VP16* animals. These panels show the range of rescue phenotypes produced by over-expression of the So-VP16 chimeric protein. Yellow arrow in panel A shows a stalk eye. (D) SEM image of adult *Drosophila* compound eye and head from *eya*^*2*^*; ey-GAL4*, *UAS-so* animals. Over-expression of So does not rescue the *eya*^*2*^ mutant. (E) Light microscope image of a developing eye-antennal disc from *eya*^*2*^*; ey-GAL4*, *UAS-so* animals. Over-expression of So does not restore Eya expression to the eye disc of *eya*^*2*^ mutants. Anterior is to the right in all adult head and imaginal disc images. At least 100 adult heads and 30 imaginal discs were examined for each genotype. Scale bar, 100μm.
(TIF)

S2 Fig The expression pattern of the composite enhancer is not altered by changes in its genomic location. (A-D) Light microscope images of developing eye-antennal discs demonstrating that placement of the composite enhancer-lacZ in a second genomic position (PBac(y^+^-attP-9A)VK00019) does not alter the expression of the construct. Red = F-actin, green = lacZ, blue = Elav. Anterior is to the right. At least 30 imaginal discs were examined. Scale bar, 100μm.
(TIF)

S3 Fig Enhancer 2 also contains the core promoter of *eya*. (A-F) Light microscope images of wild type eye-antennal discs containing enhancer-lacZ constructs. White arrowheads mark the position of the morphogenetic furrow. (A-B) *Enhancer 2—lacZ* reporter in a vector lacking a promoter shows expression mostly in photoreceptors. Ectopic expression in the antenna and ahead of furrow is lost. (C-D) The *composite enhancer lacZ* reporter in the vector lacking a promoter shows identical expression to that of a vector containing a minimal hsp70 promoter. Therefore, enhancer 2 contains the core promoter of *eya*. (E-F) The *enhancer 1+E—lacZ* reporter construct fully recapitulates Eya expression. Anterior is to the right. 30 imaginal discs were examined for each genotype. Scale bar, 100μm.
(TIF)

S4 Fig The composite enhancer is not responsive to So-VP16. (A-D) Light microscope images of developing eye-antennal discs from *dpp-GAL4*, *UAS-So-VP16* animals. The rose colored arrows in panels B-D point to cells that fail to activate the composite enhancer even in the presence of So-VP16. (E-H) Light microscope images of developing eye-antennal discs from *dpp-GAL4*, *UAS-ey* animals. The yellow colored arrows in panels F-H mark the activation of the composite enhancer by forced expression of Ey. Red = F-actin, green = lacZ, blue = Elav (photoreceptors). Anterior is to the right. At least 30 imaginal discs were examined for each genotype. Scale bar, 100μm.
(TIF)

S5 Fig Transcription of *eya* is dramatically reduced in *so*^*1*^ mutants. qRT-PCR quantification of *so* and both *eya* RA and RB transcript levels in wild type and *so*^*1*^ eye-antennal discs. Raw data from single runs of three biological replicates were used to generate the graph. The Y-axis is the relative expression levels of each transcript. Error bars indicate standard error.
(TIF)

S6 Fig *eya* expression weakens and is eliminated from progressively older *so*^*1*^ mutant eye-antennal discs. A graph quantifying the number of discs that have Eya protein within the eye field at different developmental stages. 28 discs were examined at 72hrs, 52 discs at 96hrs, 63 discs at 120hrs, 89 discs at 144hrs and 40 discs at 168hrs. AEL = after egg laying.
(TIF)

S7 Fig Conservation analysis of *eya* composite, PSE and 4 enhancers. (A-C) Evoprinter conservation analysis of *eya* enhancers. Black letters represent bases in the *D*.*melanogaster* reference sequence that are conserved in the *D*. *sechellia*, *D*. *simulans*, *D*. *yakuba*, *D*. *erecta*, *D*. *ananassae*, *D*. *persimilis*, *D*. *pseudoobscura*, *D*. *virilis*, *D*. *willistoni*, and *D*. *grimshawi* orthologous genomic regions. Blue underlining indicates single-copy repeats and red underlining identifies multi-copy repeats. (A) Conservation analysis of *eya* composite enhancer. Purple outline is enhancer 1, rose outline is enhancer 2, orange outline is *so* binding site described in [32]. (B) Conservation analysis of *eya* PSE enhancer. Orange outline is *so* binding site described in [32]. (C) Conservation analysis of *eya* enhancer 4. Orange outline is *so* binding site described in [32].
(TIF)

S1 Table Sequence of *eya* genomic fragments that were fused to lacZ and used to search for new retinal enhancers. Sequences labeled in red failed to drive expression of the reporter with the retina while sequences listed in green represent regions containing *eya* retinal cis-regulatory elements.
(DOCX)

S2 Table A list of primer sequences that were used to clone genomic fragments into vectors containing either a lacZ transcriptional reporter and/or the *eya* RB cDNA isoform. Sequences thar are listed in red were unable to drive expression of the lacZ reporter in the retina. Sequences listed in green define *eya* retinal enhancers.
(DOCX)

S3 Table A list of sequencing primers that were used to determine the breakpoints of the *eya*^*1*^ deletion. (DOCX)

S4 Table A list of primer sequences that were used to detect *so*, *eya* RB and *eya* RA transcripts using qRT-PCR. (DOCX)

S5 Table A feature list of the eya locus. Included in this list are the positions of So binding sites, So ChIP peaks, position of retinal enhancers, as well as the position of introns and exons.
(DOCX)
