# Supplementary figures and images for: Retinal Expression of the Drosophila eyes absent Gene Is Controlled by Several Cooperatively Acting Cis-regulatory Elements
Source: PLoS Genet. 2016 Dec 8;12(12):e1006462. doi: 10.1371/journal.pgen.1006462 (PMC5145141; doi:10.1371/journal.pgen.1006462)

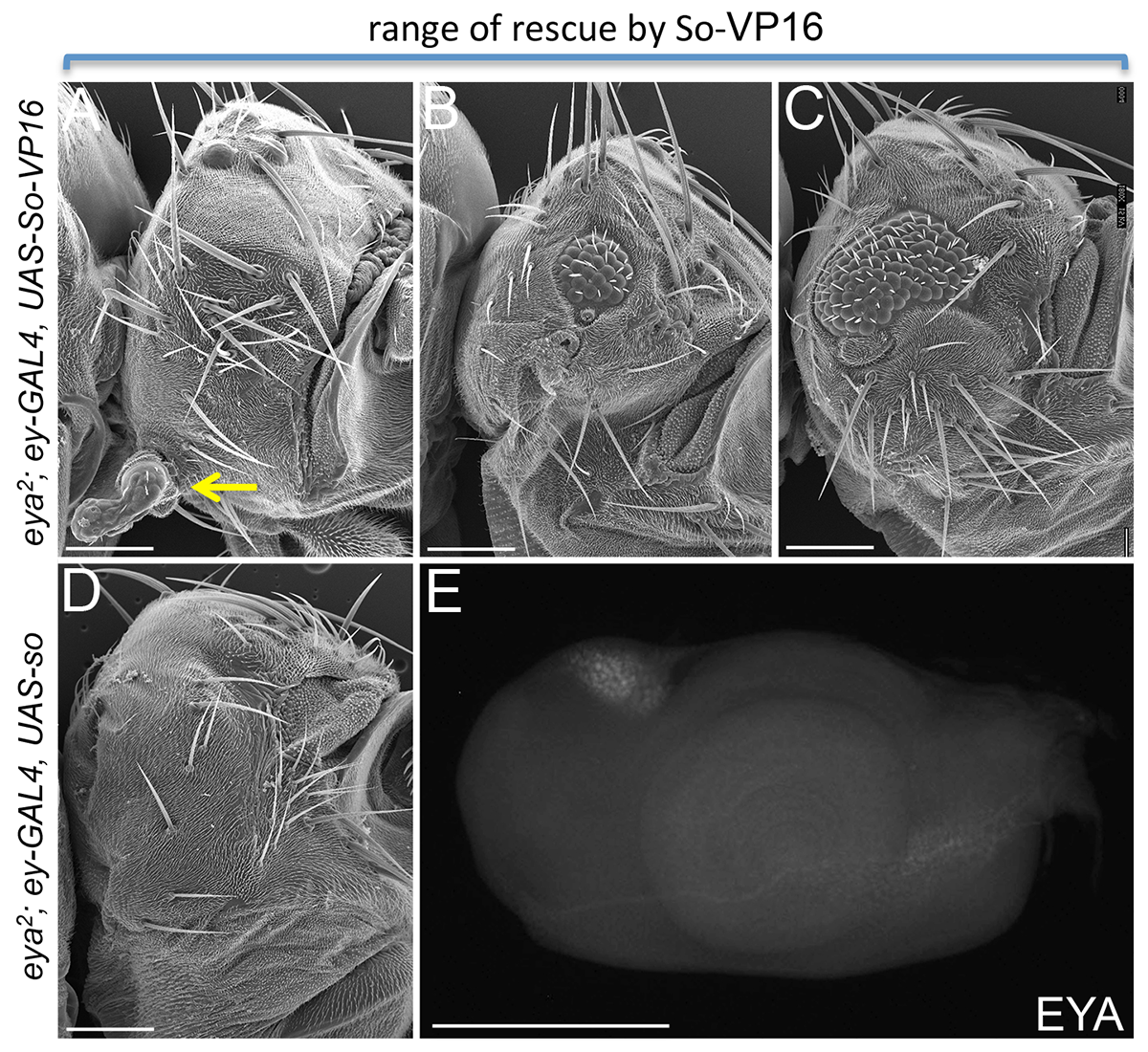

Supplement: S1 Fig — (A-C) SEM images of adult Drosophila compound eyes and heads from eya2; ey-GAL4, UAS-So-VP16 animals. These panels show the range of rescue phenotypes produced by over-expression of the So-VP16 chimeric protein. Yellow arrow in panel A shows a stalk eye. (D) SEM image of adult Drosophila compound eye and head from eya2; ey-GAL4, UAS-so animals. Over-expression of So does not rescue the eya2 mutant. (E) Light microscope image of a developing eye-antennal disc from eya2; ey-GAL4, UAS-so animals. Over-expression of So does not restore Eya expression to the eye disc of eya2 mutants. Anterior is to the right in all adult head and imaginal disc images. At least 100 adult heads and 30 imaginal discs were examined for each genotype. Scale bar, 100μm. (TIF) [file pgen.1006462.s001.tif]

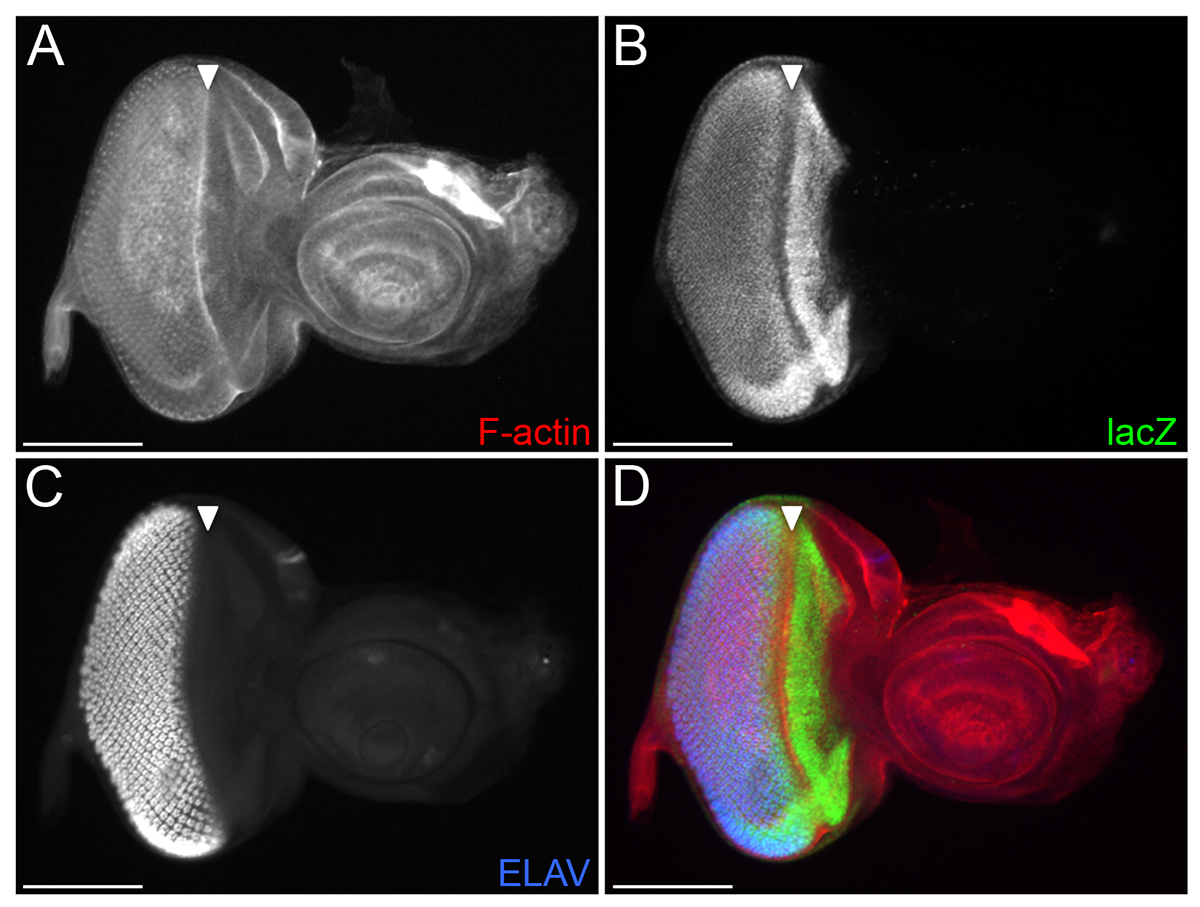

Supplement: S2 Fig — (A-D) Light microscope images of developing eye-antennal discs demonstrating that placement of the composite enhancer-lacZ in a second genomic position (PBac(y+-attP-9A)VK00019) does not alter the expression of the construct. Red = F-actin, green = lacZ, blue = Elav. Anterior is to the right. At least 30 imaginal discs were examined. Scale bar, 100μm. (TIF) [file pgen.1006462.s002.tif]

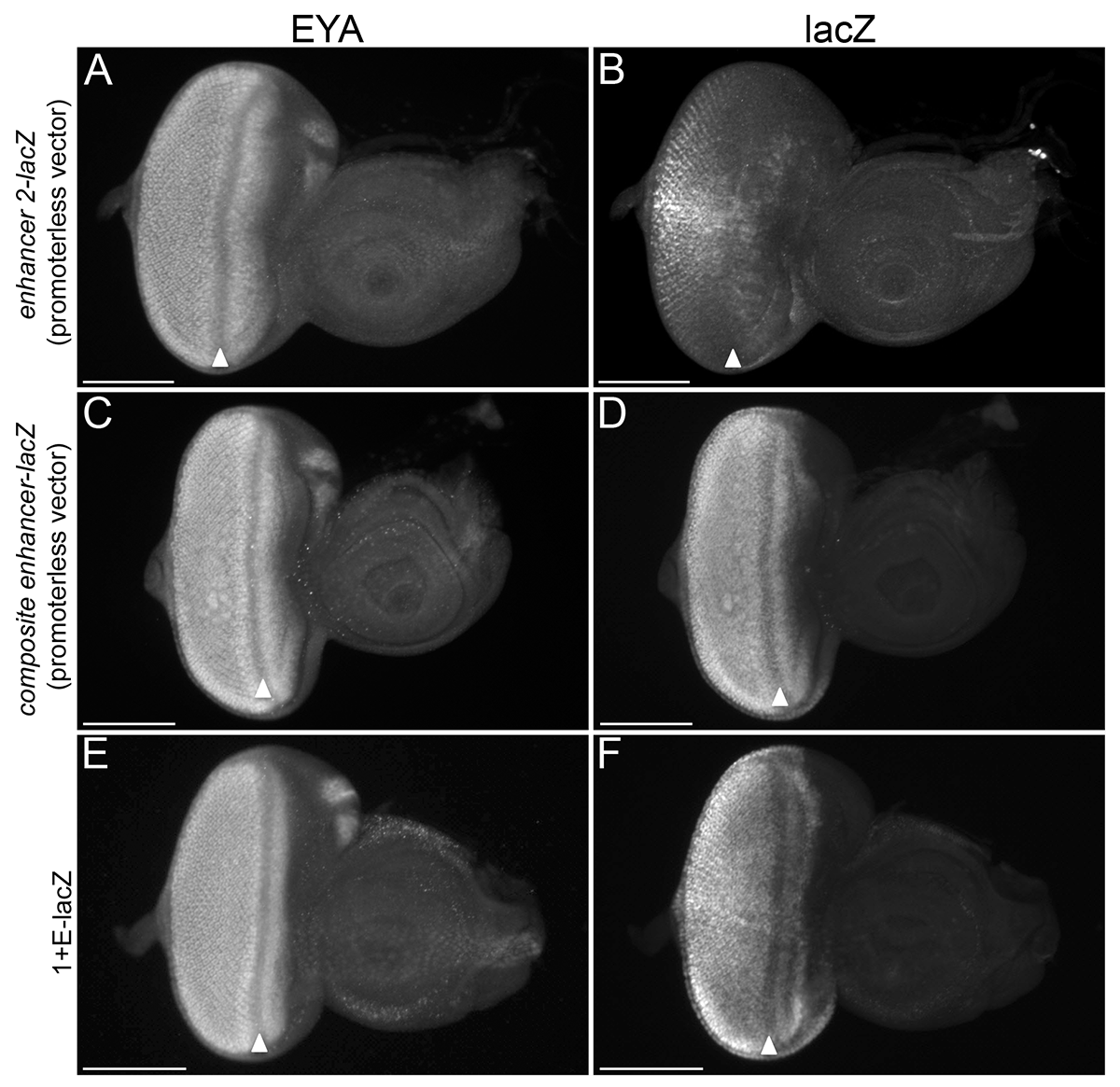

Supplement: S3 Fig — (A-F) Light microscope images of wild type eye-antennal discs containing enhancer-lacZ constructs. White arrowheads mark the position of the morphogenetic furrow. (A-B) Enhancer 2—lacZ reporter in a vector lacking a promoter shows expression mostly in photoreceptors. Ectopic expression in the antenna and ahead of furrow is lost. (C-D) The composite enhancer lacZ reporter in the vector lacking a promoter shows identical expression to that of a vector containing a minimal hsp70 promoter. Therefore, enhancer 2 contains the core promoter of eya. (E-F) The enhancer 1+E—lacZ reporter construct fully recapitulates Eya expression. Anterior is to the right. 30 imaginal discs were examined for each genotype. Scale bar, 100μm. (TIF) [file pgen.1006462.s003.tif]

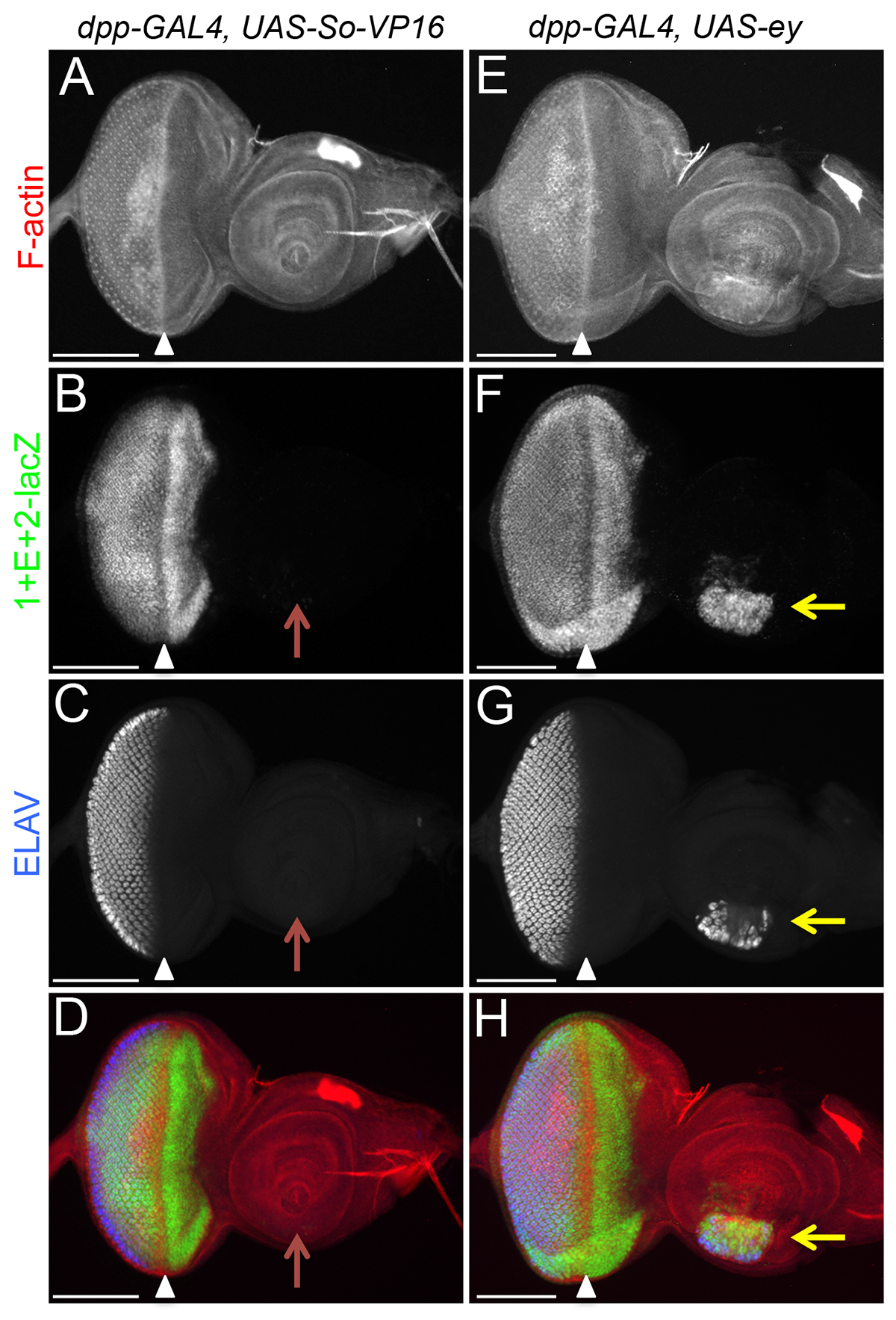

Supplement: S4 Fig — (A-D) Light microscope images of developing eye-antennal discs from dpp-GAL4, UAS-So-VP16 animals. The rose colored arrows in panels B-D point to cells that fail to activate the composite enhancer even in the presence of So-VP16. (E-H) Light microscope images of developing eye-antennal discs from dpp-GAL4, UAS-ey animals. The yellow colored arrows in panels F-H mark the activation of the composite enhancer by forced expression of Ey. Red = F-actin, green = lacZ, blue = Elav (photoreceptors). Anterior is to the right. At least 30 imaginal discs were examined for each genotype. Scale bar, 100μm. (TIF) [file pgen.1006462.s004.tif]

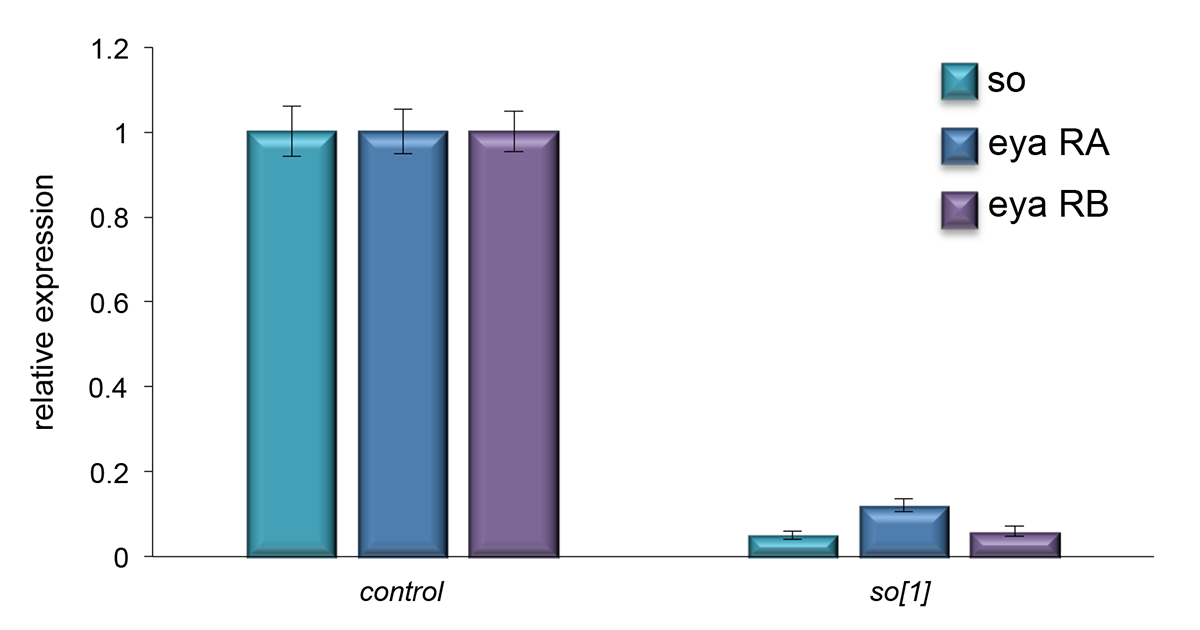

Supplement: S5 Fig — qRT-PCR quantification of so and both eya RA and RB transcript levels in wild type and so1 eye-antennal discs. Raw data from single runs of three biological replicates were used to generate the graph. The Y-axis is the relative expression levels of each transcript. Error bars indicate standard error. (TIF) [file pgen.1006462.s005.tif]

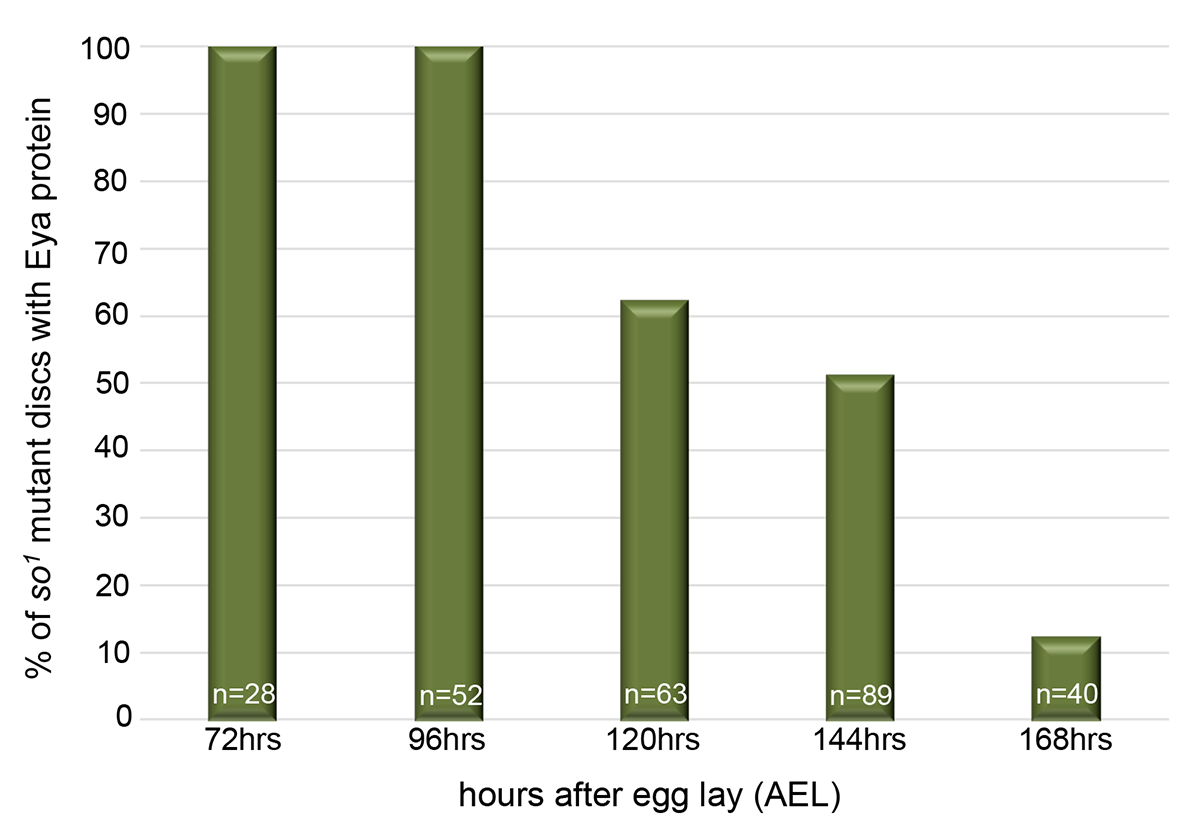

Supplement: S6 Fig — A graph quantifying the number of discs that have Eya protein within the eye field at different developmental stages. 28 discs were examined at 72hrs, 52 discs at 96hrs, 63 discs at 120hrs, 89 discs at 144hrs and 40 discs at 168hrs. AEL = after egg laying. (TIF) [file pgen.1006462.s006.tif]

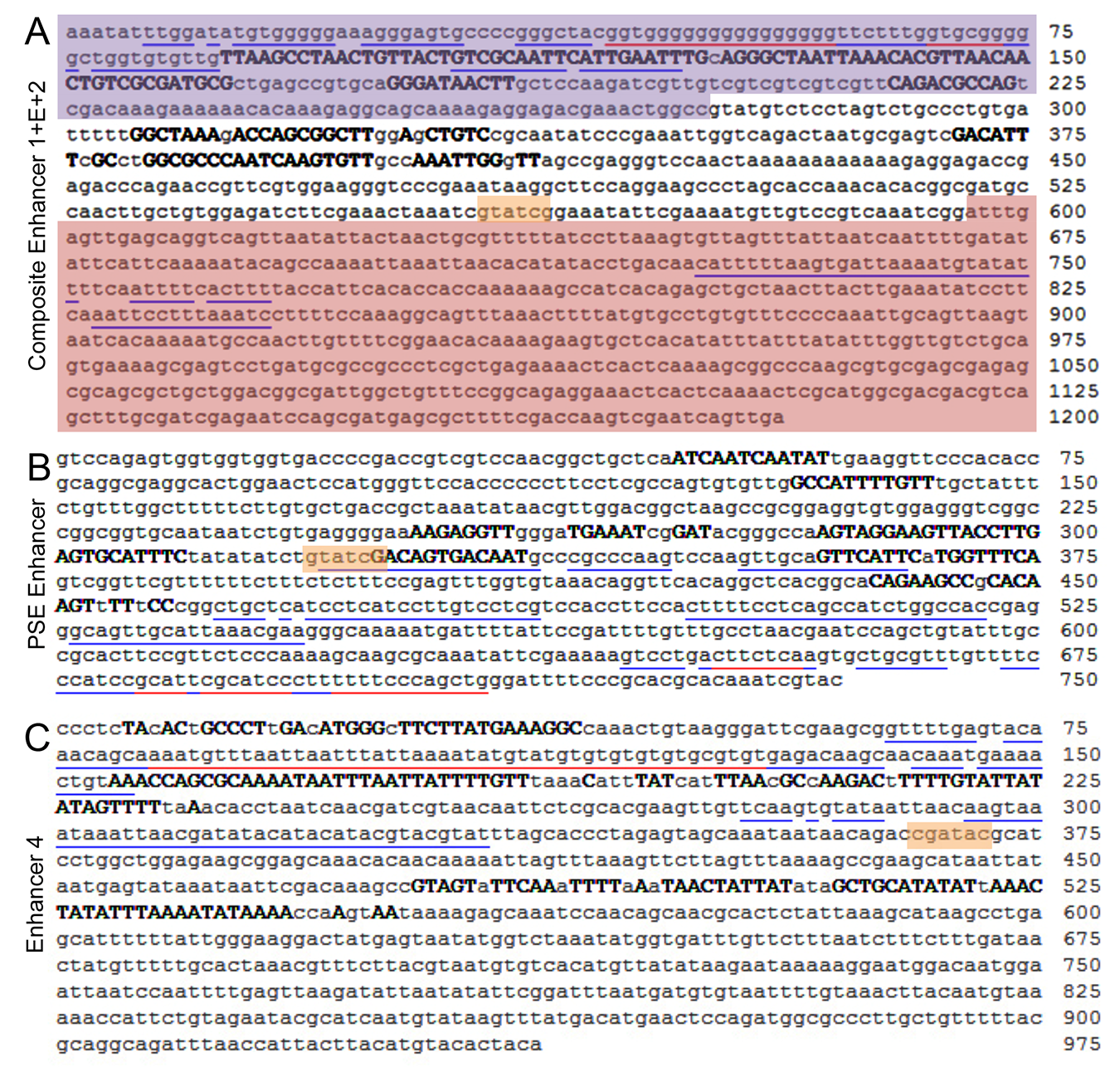

Supplement: S7 Fig — (A-C) Evoprinter conservation analysis of eya enhancers. Black letters represent bases in the D.melanogaster reference sequence that are conserved in the D. sechellia, D. simulans, D. yakuba, D. erecta, D. ananassae, D. persimilis, D. pseudoobscura, D. virilis, D. willistoni, and D. grimshawi orthologous genomic regions. Blue underlining indicates single-copy repeats and red underlining identifies multi-copy repeats. (A) Conservation analysis of eya composite enhancer. Purple outline is enhancer 1, rose outline is enhancer 2, orange outline is so binding site described in [32]. (B) Conservation analysis of eya PSE enhancer. Orange outline is so binding site described in [32]. (C) Conservation analysis of eya enhancer 4. Orange outline is so binding site described in [32]. (TIF) [file pgen.1006462.s007.tif]
