# Supplementary material for: Retinal Expression of the Drosophila eyes absent Gene Is Controlled by Several Cooperatively Acting Cis-regulatory Elements
Source: PLoS Genet. 2016 Dec 8;12(12):e1006462. doi: 10.1371/journal.pgen.1006462 (PMC5145141; doi:10.1371/journal.pgen.1006462)
Supplement: S1 Table — Sequences labeled in red failed to drive expression of the reporter with the retina while sequences listed in green represent regions containing eya retinal cis-regulatory elements. (DOCX) [file pgen.1006462.s008.docx]

**Fragment Sequences**

-8714 to -6333 (2379bp)

GTAAACATGTTGGCGACTCGAATTCAAACATGGCCGGCAAGCCAGCGAAAGAACGCTCCTCCGTTTTTCGTGCAGGGCTCCCGAGTTGAATTACACCGCAGGCGATTTTTGGAGTGCGGGAAACACAAGACCTGTGGGAAGTAAAGCTATATAGCTGCACTTATGCGCGGCTCAGCCATCGTTTAGTACGAAAATTGGTTACTTAACACAATTAATTATTTGTATTAATATTTCCCCGTACGCCAAGGGGTGCCTCCGCGCCACGCAAATCTCCCAAGGGAAATCCTGAGTAGCTGTTCTGTTTTTAGGTGTCAACGATTTGCTTCCGCTTTTCCGGAGGGGGCGACTCCTGCTATCTCCCTGCTAATTGGGATGAGAACTAGGGTGTGGGAGTATCTAGTGTGTGAGAAACCACTAGTTAATGCTTGTCACAACAAATAGTGTCATAAATCTGGACTCCCAAGGGGGATGCAATTCCAGGGAGTGGAGTCCTGTAAAAAATACTTCTCCACAACTGGCGGTTGTAAATTTTTTAGGTTTTCTTTTGGAGAGAAAATGTTTCCCTGGAGTGCGTGGATCCTTGGGACATGTTTGGGACTGGGACTTTTACCTGAAGACCAGTTGTTGACAACAAGATAAGTTTTAAATTAAGTTTTAACTTGCTCATATTTTATTCATTCTCATTTCAATAAATTTCCATTCCAAATGGACATCTTTTAGTTAGTTACAGAAATTCGGAATATTATCCAAAATATTACACTATATTATGGATTATTTACAAAATGTTCCTGTTTTGAAGTCATTTCACCCCGAATCCCCACTTCGAACCGTCCAGAGTGGTGGTGGTGACCCCGACCGTCGTCCAACGGCTGCTCAATCAATCAATATTGAAGGTTCCCACACCGCAGGCGAGGCACTGGAACTCCATGGGTTCCACCCCCCTTCCTCGCCAGTGTGTTGGCCATTTTGTTTGCTATTTCTGTTTGGCTTTTTCTTGTGCTGACCGCTAAATATAACGTTGGACGGCTAGGCCGCGGAGGTGTGGAGGGTCGACCGGCGGTGCAATAATCTGTGAGGGGAAAAGAGGTTGGGATGAAATCGGATACGGGCCAAGTAGGAAGTTACCTTGAGTGCATTTCTATATATCTGTATCGACAGTGACAATGCCCGCCCAAGTCCAAGTTGCAGTTCATTCATGGTTTCAGTCGGTTCGTTTTTTCGTTCTCTTTCCGAGTTTGGTGTAAACAGGTTCACAGGCTCACGGCACAGAAGCCGCACAAGTTTTTCCCGGCTGCTCATCCTCATCCTCGTCCTCGTCCACCTTCCACTTTTCCTCAGCCATCTGGCCACCGAGGGCAGTTGCATTAAACGAAGGGCAAAAATGATTTTATTCCGATTTTGTTTGCCGAACGAATCCAGCTGTATTTGCCGCACTTCCGTTCTCCCAAAAGCAAGCGCAAATATTCGAAAAAGTCCTGACTTCTCAAGTGCTGCGTTTGTTTTCCCATCCGCATTCGCATCCCTTTTTTCCCAGCTGGGATTTTCCCGCACACAAATCGTACTTTATATATTAGTCACTTTTCCGAACTGTCCACCATTTCTCACCTGTATGAAGCTCGCCAGTGGGGTGGGGGGGGTGCTGAAATTCCGGGAAAACCCTCATGGAGCACGCCGCGATCGTGATGCGGGTCGTGGCGACATCGTTTTAATTAAATTACAGCCACCAAAACGTGCGACACTATAATCATTTTTATTCATTGTCAAAAATGGGCAATCATAAAAAACAACACGTCTGCATCGCAGCTGGCGAAGGTGGAGTTCTAAGTTAATCCGTGGTGGGGGTTAATCCCACGGAGTGTGGGGCAAGCTGTCCGGACACAGCCACCAACATAAACAGCCATTCGCTCGCACAAGGGGAATGTAACAATCAATTAACAGGCCAGATAGGTTCAGATCGGAGATTCCAGTTAACCCTTGGGGAGCATCACTTTTTGGCTTCATTTCAGGTCGTAATTTTAACGAGCTATAGGTACTGGGATTATGCAGTTTTAAGTAAAATAACTTATCATAAATTTGTATGTCATGTCATCATTAAAGCTTTAAATGATTGTCATGATCTTAGGATTTATTTTTGTCTGCATAGCCTCTTGAGTGAATGAGTGGTGTGATGGGAACCCTCTTGCCCATGAAAGGGTTAAAGCAGCCGTTGACCCAAGATTGTCATGGCACGCTTCTTCGTCCGCTGGACTCTCTGTCTTTGTCTTTGTCTCCGTGGATGTCGCTCCACTCGTGTTGTTGTTGTTTCTGTCTGCTGGCGGGGACATGTGGTTGTTGTTGCCATCGCTGTGGGGCCCAATGTCAATGTCTATATGGCCATC

-6332 to -3831 (2501bp)

ATCGGAATAGAAAGCGTGTCTTTTATGTTGTGGCATTGTTATTAATCTATCATTAGGTCGCTGTTGTACAACAGGCTCTAATGATGCGTGTAAATGCTAAACAAATCACACATTCTGCCCAAAAAAAAAACTATATAAAAATAAAATACACCAAATCCAAATCGAATATCTCTGCGCAGCGAAACGAGGGAAAAATGCTCGAAGCGCGTGTACAATTTAAAATGAAATAAAATCAGACTGCGCAGCTTTCGAAATTTTATGAGCTTGTTGTGCTTATTAGTCGCCCCACGAGAAATGGCGACAGGCCACAACATCACCAACAACATCACCAACAACACCACCGAAACAACAACACCAATGAGAACAACCAGAACGGTAACAACAACAACAACAACAACAATCACAACACGCATTGCCACGGCTAATTTTTCACGCGCACTCCGAATGCAGCTTCACCTCCAGCTCCATACCTGCTCCACCTCCATACCAGCTCCAGCTCCAACTCCATCTCCATTCCGACAGCGACAGCTCCTGACCAGGCTATCACTTGCCGCAGTCCCAGCAAGGGTATCATTGCTATCCTGTAGCCTTTGTGCTAGTACTGGTCTCGATTTTGATTTAGCGTTAGTTGTGCAGTATTCAATGATGCAAGTGAAGTTTTCCCAATGGAAAACTAATTCAACGGGGATTTAATGTATAGCAAAGGCATTGAGTGAAATGGAAAATCGAAATATATGGGTGCATACTTTTGGACAGCTTCATAATAGTATCAGTTTACGATGTTCGAACTGCCTGACGCATAATTACAATCATTTCTTTGTTTTTCACTTATTATATTGATAGCATTAGAATAAACTTCGATAATTTAATTATTGATATGATATATGTACATAATATGTAGTTCAACCAGCATTCGATGATCTTCAATTGGCTGCTTGAGGTTCCTTGTGCTTTATTTTTATACTTTTTGGCACGCCAGACACGCACGAGAAATTCCAAGAGATAAGGATCGTGTACGACAAGGTGTATTATGGACCCCCAATCCAGGGACCCCAACCCCGGCAGCCAAATGCCCAGTGTCCCCCAGTCGGAGTTGTGCCGTCGAGTGCCATACTGTTGACTCTGTTAAATTGCCAATATTTATGGTCTTGCCCGCACGGGTTGACTTGTTAAATGGGTGTTCCTTCCAGCAGCCTTTCGTTGGATGCCATCACCAGTTCCGTGGCCGGAGGATAGGGACTCCTGCCCGACAGTTGGTAGCTGTAAAACGTTTAAGGACAAAACGTGTTCGGCCCGATAAGAGGCAGCTGGCCAAGACGATGGGCTACGGCCATCAGCATCAGTTTGACGTATGCCCGTCAATGGTTTTAATGGCCCAGCTAGCCATTTTGAAAGTGCCAAGATTCAGTTTCTATGTGCAGCTAAGCAGAAATGCGCAATGTCGTCGCCGCTGAAGCTATAAAAATGTCAAAACATGCAACAAATAATGGCAGAATTATAAAGAAATCTAGCGGTAGCGAAAATAAATAAATAAAAAAAAGCGCGTCATTCAGTTCGTTCAGTGCAGTAAGCCAGTTGTTTGCGGCTTATTCCCTACAAATTAATTCGGTAGCTTGATTTCTGGAAATCTTATAATAACGAGTGCATACTGTACTTATTTATTACTTAGTTAAGTGAGAAGCATGCTACCAAATACGCCATTTTAATAAGAAATTTCAAACTCAATAAATATTAATATATAAACTTAATAGGATTTCAGAGCATTTATACTTCTTGCAGACCGAATTTATTCCCCATCACCGCCCTTATCTCACTGGATGTGTAATTGACAAAAAACGAAAAAATGAGTTTCCTCTGGCTTGTTTCCAGGTTGATACGGCGCTGATCTTGCGGTTTTGCTGGTTTTATATCGCATTTATGCCATTTTCCATGGTAGTTTTTTTTTTTAGTTTATTTCGGCGAGGAGCCGCACGCGAATCTGCCATGATGCTGACATTTCGAAATCACCCGAATCCCTTCTGAAACCGTAGATGTGTGAGAGAGGAAAGCCCAGTCTTATCTCGATCCGAATGATCGTCACCTTAAATGCTATAAGTCACACCTAGCCATTTGTCACATTTTCTAGCCGCCCACGGGGCCAAACCAATGGGCATGGGGCATCGGACATCGGGGATTGGGATCGGAGATTGAGACTTGTTTCATAAATCAAATCGGTGCACGTTACATTTGAGGCGCATAAAAATGCAAATTGTATGCATAAAGTATAATTTTTATAACTTAATTAACTGAGCTTTGCGGCGAAGTGGGCTTGGGGCACCTGCTGTGTTTGTCGCTTTTTCCTCATTAGGCTGGCGCCAAGTTTGAGATTGATTTTGGTCGCATTTACATGACAAAGCTGCTGCGGATGCTGTAGGAGTTGTCTTGGGTCGATTTGGTTGTGATAAGCTCATCAGGGCACCACCACTGGTAGCTGCAGCTCCTCGGGAATCAGCCAGGCCAAG

-3830 to -897 (2934bp) Enhancer 1 Large Fragment

ACTACACCTCGTACCAAATTCTCGGAAGAGCTGCAGATAAGCCGAGTGCCCCAGACGAACATTAAATAATCTGCCTCTAATTTGGGTGTCGTTTACACGTGCCAAGATCTCTTGACTCCACTCCACTTCACTTCACTGCCGACTCCCTGACACTGATGAGGCCCATCAAGATGATGATGGGTCGAGTGTTGGGTCCCCGGCAGGGTCATTAAACACCTGGCATTATCGCTTCATCTCGGAATGTGCCTGATATGCGATTCGAGGGGGTGGCGTTGTGTCGAACTGGCGGAATGTCAAAGTTTACATATTTGATTGGATTTTATGGGAATTTCCAAGGTATGTTTGTGTTTGACGATCCTCTGAAAAAGCAATGCTCCCTAAAATAGTATAGCCAAGGATTACACCTATCATAATACCCGTAATTAACCACTACGTAAGCCATCCTTAGCCTTTTATGCCTTCAGACAGAGTTTATTTTGTTTATTTATATTGTGTAATTTGTTATTTCTTCTACCTCTCCTTTGTGGGCCCTGTTTCTCTGCTGGCTGACACTTGACCCTTTTAGAAAATAACCAGTTGTGTGTGTGTGTGTGCGACGATGGGGTGAATGTTGGTGACTGTCTCGAAATCGAAATCTGGCGCCGTTTACATTGCCCGCGGCTAAACAAAAAAGAAACATCAGCCAAATCCACAATCGAGAATGTCTATACAAAAGTCAATAAACTTCAGTGCGATAGGTATGTGGACAACAGCCACAATCACAAGGGCAGCAAAATCACAAACAATGGTCACGACAACGGGGGGCGTTCGTCTGGAAGGGATCAGCCAAGGGGTTTCCCATGGAGGTGGTTACAAGCAACAGGTGCAATGGGGCAGCACACTACTTATTTCTAGCCACAAGTTGAATTAAATCACTTATCTAAATATGATCTTATAGTGAATCGCTTTGGTTTAGTCATTAAACTGAACTGGGTATCTCCTCCTGCTGTTTCCTAAGAGACAAAAGCAATGTGTTCCATATCAGAGTACTTTAAACTTTGCCTTAGCTTTTCCACTGAACAGCTCACTCCACTCCACCCGATGGGTGAAGCCTTGAAAACCCACCACCTTCCAGTGCTGACTCATCCGGGCAGCTGGCTGTGGGCTCTTCCCGCTGGTGACTTACTGTCAACTGGCGCTTGGGCCGAACTCGTTGGTCATAAATTATCCGAAAGGAGGACCCTTTGCCTGCGCATCCCAATTGGGGACCAGCTCCTACTTCTTCTCCAGCTCCTCCTCCAGCTCACTGTCGCCAGCTCGGATGCCAAGTGATTTTTTTTTCTTAGCGCGCCCAGCGGGCGCTCATTGTACGGGCAGTTAAACAAGACGCTTTTCAGCTCGTAAAACAAGCTGGCTGGCTGTTTGTTTTTGTATAATAAAGTTATTGATTTGTGTTTAAGTGCCGTGGGTGACCCGCTTGGAAACCCAACGGCGATTTGGGGTACGAGAAGAAAGCCTTTCCCCCAGCAGATTAATGCGTTGCATTCGGGGCCAACAATCGCTGGCATTTATCAGCTTATTTGCTTTATTAAACGCTAAGCTGAGCCGTTTAAGGCCCAAAGCTGACGCCTGTCAATTGCGACATGAGCAAAGTCACTCCCCTCCAGAAATCGCTGCCCACCACTGCTCGATGCTCGACTCCTGATCTTTGGGCTGTGGACAGCGGACTGTGGGCGGCGCACTTAAGTAGCTTAAACAGACGCGGGGTGGTCCGCGACATAACTTTGTCTAATGACTCGGAAATCCGTCACTAATAATAAGAGCCATGGCCATAAAGAGGCAGCGAGCATTTGAGACCCTCAGTCGGTCAGCGACTGCCGATGCATTTCTGCACTTAACTGGAATCGGTCAACTTCCAGCTTCAGATGGGAGCTTCCATCTTGGAGCTACAGATGGTAGAGGCCGAAGCCTCTGACACTGAGCACGTGTGTGTGCTTCTGGCATAATCCTATTACCCAGCCGACGTTGCTCGAGTTTTATGAATGAAAATATTCCTACGACCGCAGCAGGCAAACAAATGGTTGTTGAAAAATAAGAGTAAATCCAATTGAAGGCAATCGATGGAGTAGTTGGGCAGGTTGATAGGCAAATGCCATGGATTGGGTTGGAAAATCAATAATAGACTACTTTATAACTTCATCGATTGCAACTCAAAGTGAGGTTTAAGTGAGTTACGTGAGTGATTTAAAAAAAAAAATAATTATATAAGTTATACTATTTAAGGGAGCTAAGTATTTAAGTCCCTGTTCTCAGCATTCAGCATTTCTTAGTTTATATATGTTTTTGCATTATACTAATGATTTCCTTTTGCTGTAACTTGCGTATCGTGCCGATCCCATCTTATCTGACTCATTAAAGGAAGCTATGCCTCCGCCCCGCCAAACCAGGAAACCAATTTAAAGCCACACTGTAGTTTATGGCTTAAAGTCGGATGGAATTAGGAAAACGCCCCCTTCTCCATTCGCCCCATTGTAGCACCATTCCATCCCTTCAATCCCAATTCCACACCTACAAGAGTCAACATTGAGTGCTTTTCCGCGCGCATGCGTTCGATCCTCGTCTGCCGACGGGGGAGGATTCCATGTCCTCGGAGCACCAGGACAATTCAAAATGGCTGCACAAATATTTGGATATGTGGGGGAAAGGGAGTGCCCCGGGCTACGGTGGGGGGGGGGGGGGGTTCTTTGGTGCGGGGGCTGGTGTGTTGTTAAGCCTAACTGTTACTGTCGCAATTCATTGAATTTGCAGGGCTAATTAAACACGTTAACAACTGTCGCGATGCGCTGAGCCGTGCAGGGATAACTTGCTCCAAGATCGTTGTCGTCGTCGTCGTCCAGACGCCAGTCGACAAAGAAAAAACACAAAGAGGCAGCAAAAGAGGAGACGAAACTGGCC

-1171 to -897 (275bp) Enhancer 1 Minimal Fragment

AAATATTTGGATATGTGGGGGAAAGGGAGTGCCCCGGGCTACGGTGGGGGGGGGGGGGGGTTCTTTGGTGCGGGGGCTGGTGTGTTGTTAAGCCTAACTGTTACTGTCGCAATTCATTGAATTTGCAGGGCTAATTAAACACGTTAACAACTGTCGCGATGCGCTGAGCCGTGCAGGGATAACTTGCTCCAAGATCGTTGTCGTCGTCGTCGTCCAGACGCCAGTCGACAAAGAAAAAACACAAAGAGGCAGCAAAAGAGGAGACGAAACTGGCC

-896 to -577 (320bp) Extant Enhancer

GTATGTCTCCTAGTCTGCCCTGTGATTTTTGGCTAAAGACCAGCGGCTTGGAGCTGTCCGCAATATCCCGAAATTGGTCAGACTAATGCGAGTCGACATTTCGCCTGGCGCCCAATCAAGTGTTGCCAAATTGGGTTAGCCGAGGGTCCAACTAAAAAAAAAAAAGAGGAGACCGAGACCCAGAACCGTTCGTGGAAGGGTCCCGAAATAAGGCTTCCAGGAAGCCCTAGCACCAAACACACGGCGATGCCAACTTGCTGTGGAGATCTTCGAAACTAAATCGTATCGGAAATATTCGAAAATGTTGTCCGTCAAATCGG

-576 to +10 (586bp) Enhancer 2 Large Fragment

ATTTGAGTTGAGCAGGTCAGTTAATATTACTAACTGCGATTTTATCCTTAAAGTGTTAGTTTATTAATCAATTTTGATATATTCATTCAAAAATACAGCCAAAATTAAATTAACTCATATACCTGACAACATGTTTAAGTGATTAAAATGTATATTTTCAATTTTCACTTTTACCATTCACACCACCAAAAAAGCCATCACAGAGCTGCTAACTTACTTGAAATATCCTTCAAATTCCTTTAAATCCTTTTCCAAAGGCAGTTTAAACTTTTATGTGCCTGTGTTTCCCCAAATTGCAGTTAAGTAATCACAAAAATGCCAACTTGTTTTCGGAACACAAAAGAAGTGCTCACATATTTATTTATATTTGGTTGTCTGCAGTGAAAAGCGAGTCCTGATGCGCCGCCCTCGCTGAGAAAACTCACTCAAAAGCGGCCCAAGCGTGCGAGCGAGAGCGCAGCGCTGCTGGACGGCGATTGGCTGTTTCCGGCAGAGGAAACTCACTCAAAACTCGCATGGCGACGACGTCAGCTTTGCGATCGAGAATCCAGCGATGAGCGCTTTTCGACCAAGTCGAATCAGTTGA

-282 to +10 (292bp) Enhancer 2 Minimal Fragment

TGCAGTTAAGTAATCACAAAAATGCCAACTTGTTTTCGGAACACAAAAGAAGTGCTCACATATTTATTTATATTTGGTTGTCTGCAGTGAAAAGCGAGTCCTGATGCGCCGCCCTCGCTGAGAAAACTCACTCAAAAGCGGCCCAAGCGTGCGAGCGAGAGCGCAGCGCTGCTGGACGGCGATTGGCTGTTTCCGGCAGAGGAAACTCACTCAAAACTCGCATGGCGACGACGTCAGCTTTGCGATCGAGAATCCAGCGATGAGCGCTTTTCGACCAAGTCGAATCAGTTGA

-3830 to -897 + -576 to +10 (3520bp) Enhancer 1+2

ACTACACCTCGTACCAAATTCTCGGAAGAGCTGCAGATAAGCCGAGTGCCCCAGACGAACATTAAATAATCTGCCTCTAATTTGGGTGTCGTTTACACGTGCCAAGATCTCTTGACTCCACTCCACTTCACTTCACTGCCGACTCCCTGACACTGATGAGGCCCATCAAGATGATGATGGGTCGAGTGTTGGGTCCCCGGCAGGGTCATTAAACACCTGGCATTATCGCTTCATCTCGGAATGTGCCTGATATGCGATTCGAGGGGGTGGCGTTGTGTCGAACTGGCGGAATGTCAAAGTTTACATATTTGATTGGATTTTATGGGAATTTCCAAGGTATGTTTGTGTTTGACGATCCTCTGAAAAAGCAATGCTCCCTAAAATAGTATAGCCAAGGATTACACCTATCATAATACCCGTAATTAACCACTACGTAAGCCATCCTTAGCCTTTTATGCCTTCAGACAGAGTTTATTTTGTTTATTTATATTGTGTAATTTGTTATTTCTTCTACCTCTCCTTTGTGGGCCCTGTTTCTCTGCTGGCTGACACTTGACCCTTTTAGAAAATAACCAGTTGTGTGTGTGTGTGTGCGACGATGGGGTGAATGTTGGTGACTGTCTCGAAATCGAAATCTGGCGCCGTTTACATTGCCCGCGGCTAAACAAAAAAGAAACATCAGCCAAATCCACAATCGAGAATGTCTATACAAAAGTCAATAAACTTCAGTGCGATAGGTATGTGGACAACAGCCACAATCACAAGGGCAGCAAAATCACAAACAATGGTCACGACAACGGGGGGCGTTCGTCTGGAAGGGATCAGCCAAGGGGTTTCCCATGGAGGTGGTTACAAGCAACAGGTGCAATGGGGCAGCACACTACTTATTTCTAGCCACAAGTTGAATTAAATCACTTATCTAAATATGATCTTATAGTGAATCGCTTTGGTTTAGTCATTAAACTGAACTGGGTATCTCCTCCTGCTGTTTCCTAAGAGACAAAAGCAATGTGTTCCATATCAGAGTACTTTAAACTTTGCCTTAGCTTTTCCACTGAACAGCTCACTCCACTCCACCCGATGGGTGAAGCCTTGAAAACCCACCACCTTCCAGTGCTGACTCATCCGGGCAGCTGGCTGTGGGCTCTTCCCGCTGGTGACTTACTGTCAACTGGCGCTTGGGCCGAACTCGTTGGTCATAAATTATCCGAAAGGAGGACCCTTTGCCTGCGCATCCCAATTGGGGACCAGCTCCTACTTCTTCTCCAGCTCCTCCTCCAGCTCACTGTCGCCAGCTCGGATGCCAAGTGATTTTTTTTTCTTAGCGCGCCCAGCGGGCGCTCATTGTACGGGCAGTTAAACAAGACGCTTTTCAGCTCGTAAAACAAGCTGGCTGGCTGTTTGTTTTTGTATAATAAAGTTATTGATTTGTGTTTAAGTGCCGTGGGTGACCCGCTTGGAAACCCAACGGCGATTTGGGGTACGAGAAGAAAGCCTTTCCCCCAGCAGATTAATGCGTTGCATTCGGGGCCAACAATCGCTGGCATTTATCAGCTTATTTGCTTTATTAAACGCTAAGCTGAGCCGTTTAAGGCCCAAAGCTGACGCCTGTCAATTGCGACATGAGCAAAGTCACTCCCCTCCAGAAATCGCTGCCCACCACTGCTCGATGCTCGACTCCTGATCTTTGGGCTGTGGACAGCGGACTGTGGGCGGCGCACTTAAGTAGCTTAAACAGACGCGGGGTGGTCCGCGACATAACTTTGTCTAATGACTCGGAAATCCGTCACTAATAATAAGAGCCATGGCCATAAAGAGGCAGCGAGCATTTGAGACCCTCAGTCGGTCAGCGACTGCCGATGCATTTCTGCACTTAACTGGAATCGGTCAACTTCCAGCTTCAGATGGGAGCTTCCATCTTGGAGCTACAGATGGTAGAGGCCGAAGCCTCTGACACTGAGCACGTGTGTGTGCTTCTGGCATAATCCTATTACCCAGCCGACGTTGCTCGAGTTTTATGAATGAAAATATTCCTACGACCGCAGCAGGCAAACAAATGGTTGTTGAAAAATAAGAGTAAATCCAATTGAAGGCAATCGATGGAGTAGTTGGGCAGGTTGATAGGCAAATGCCATGGATTGGGTTGGAAAATCAATAATAGACTACTTTATAACTTCATCGATTGCAACTCAAAGTGAGGTTTAAGTGAGTTACGTGAGTGATTTAAAAAAAAAAATAATTATATAAGTTATACTATTTAAGGGAGCTAAGTATTTAAGTCCCTGTTCTCAGCATTCAGCATTTCTTAGTTTATATATGTTTTTGCATTATACTAATGATTTCCTTTTGCTGTAACTTGCGTATCGTGCCGATCCCATCTTATCTGACTCATTAAAGGAAGCTATGCCTCCGCCCCGCCAAACCAGGAAACCAATTTAAAGCCACACTGTAGTTTATGGCTTAAAGTCGGATGGAATTAGGAAAACGCCCCCTTCTCCATTCGCCCCATTGTAGCACCATTCCATCCCTTCAATCCCAATTCCACACCTACAAGAGTCAACATTGAGTGCTTTTCCGCGCGCATGCGTTCGATCCTCGTCTGCCGACGGGGGAGGATTCCATGTCCTCGGAGCACCAGGACAATTCAAAATGGCTGCACAAATATTTGGATATGTGGGGGAAAGGGAGTGCCCCGGGCTACGGTGGGGGGGGGGGGGGGTTCTTTGGTGCGGGGGCTGGTGTGTTGTTAAGCCTAACTGTTACTGTCGCAATTCATTGAATTTGCAGGGCTAATTAAACACGTTAACAACTGTCGCGATGCGCTGAGCCGTGCAGGGATAACTTGCTCCAAGATCGTTGTCGTCGTCGTCGTCCAGACGCCAGTCGACAAAGAAAAAACACAAAGAGGCAGCAAAAGAGGAGACGAAACTGGCCATTTGAGTTGAGCAGGTCAGTTAATATTACTAACTGCGATTTTATCCTTAAAGTGTTAGTTTATTAATCAATTTTGATATATTCATTCAAAAATACAGCCAAAATTAAATTAACTCATATACCTGACAACATGTTTAAGTGATTAAAATGTATATTTTCAATTTTCACTTTTACCATTCACACCACCAAAAAAGCCATCACAGAGCTGCTAACTTACTTGAAATATCCTTCAAATTCCTTTAAATCCTTTTCCAAAGGCAGTTTAAACTTTTATGTGCCTGTGTTTCCCCAAATTGCAGTTAAGTAATCACAAAAATGCCAACTTGTTTTCGGAACACAAAAGAAGTGCTCACATATTTATTTATATTTGGTTGTCTGCAGTGAAAAGCGAGTCCTGATGCGCCGCCCTCGCTGAGAAAACTCACTCAAAAGCGGCCCAAGCGTGCGAGCGAGAGCGCAGCGCTGCTGGACGGCGATTGGCTGTTTCCGGCAGAGGAAACTCACTCAAAACTCGCATGGCGACGACGTCAGCTTTGCGATCGAGAATCCAGCGATGAGCGCTTTTCGACCAAGTCGAATCAGTTGA

Enhancer 1+5bp+2 (878bp)

AAGCTTAAATATTTGGATATGTGGGGGAAAGGGAGTGCCCCGGGCTACGGTGGGGGGGGGGGGGGGTTCTTTGGTGCGGGGGCTGGTGTGTTGTTAAGCCTAACTGTTACTGTCGCAATTCATTGAATTTGCAGGGCTAATTAAACACGTTAACAACTGTCGCGATGCGCTGAGCCGTGCAGGGATAACTTGCTCCAAGATCGTTGTCGTCGTCGTCGTCCAGACGCCAGTCGACAAAGAAAAAACACAAAGAGGCAGCAAAAGAGGAGACGAAACTGGCCCTAGGATTTGAGTTGAGCAGGTCAGTTAATATTACTAACTGCGATTTTATCCTTAAAGTGTTAGTTTATTAATCAATTTTGATATATTCATTCAAAAATACAGCCAAAATTAAATTAACTCATATACCTGACAACATGTTTAAGTGATTAAAATGTATATTTTCAATTTTCACTTTTACCATTCACACCACCAAAAAAGCCATCACAGAGCTGCTAACTTACTTGAAATATCCTTCAAATTCCTTTAAATCCTTTTCCAAAGGCAGTTTAAACTTTTATGTGCCTGTGTTTCCCCAAATTGCAGTTAAGTAATCACAAAAATGCCAACTTGTTTTCGGAACACAAAAGAAGTGCTCACATATTTATTTATATTTGGTTGTCTGCAGTGAAAAGCGAGTCCTGATGCGCCGCCCTCGCTGAGAAAACTCACTCAAAAGCGGCCCAAGCGTGCGAGCGAGAGCGCAGCGCTGCTGGACGGCGATTGGCTGTTTCCGGCAGAGGAAACTCACTCAAAACTCGCATGGCGACGACGTCAGCTTTGCGATCGAGAATCCAGCGATGAGCGCTTTTCGACCAAGTCGAATCAGTTGAGGTACC

-576 to +50 (626bp) Enhancer 2 Minimal Fragment in Promoterless Vector

ATTTGAGTTGAGCAGGTCAGTTAATATTACTAACTGCGATTTTATCCTTAAAGTGTTAGTTTATTAATCAATTTTGATATATTCATTCAAAAATACAGCCAAAATTAAATTAACTCATATACCTGACAACATGTTTAAGTGATTAAAATGTATATTTTCAATTTTCACTTTTACCATTCACACCACCAAAAAAGCCATCACAGAGCTGCTAACTTACTTGAAATATCCTTCAAATTCCTTTAAATCCTTTTCCAAAGGCAGTTTAAACTTTTATGTGCCTGTGTTTCCCCAAATTGCAGTTAAGTAATCACAAAAATGCCAACTTGTTTTCGGAACACAAAAGAAGTGCTCACATATTTATTTATATTTGGTTGTCTGCAGTGAAAAGCGAGTCCTGATGCGCCGCCCTCGCTGAGAAAACTCACTCAAAAGCGGCCCAAGCGTGCGAGCGAGAGCGCAGCGCTGCTGGACGGCGATTGGCTGTTTCCGGCAGAGGAAACTCACTCAAAACTCGCATGGCGACGACGTCAGCTTTGCGATCGAGAATCCAGCGATGAGCGCTTTTCGACCAAGTCGAATCAGTTGACACGCAGCTTTATGTGACATTCGTGGCGAAAGCGGACCAC

-1171 to +10 (1181bp) Composite Enhancer

AAATATTTGGATATGTGGGGGAAAGGGAGTGCCCCGGGCTACGGTGGGGGGGGGGGGGGGTTCTTTGGTGCGGGGGCTGGTGTGTTGTTAAGCCTAACTGTTACTGTCGCAATTCATTGAATTTGCAGGGCTAATTAAACACGTTAACAACTGTCGCGATGCGCTGAGCCGTGCAGGGATAACTTGCTCCAAGATCGTTGTCGTCGTCGTCGTTCAGACGCCAGTCGACAAAGAAAAAACACAAAGAGGCAGCAAAAGAGGAGACGAAACTGGCCGTATGTCTCCTAGTCTGCCCTGTGATTTTTGGCTAAAGACCAGCGGCTTGGAGCTGTCCGCAATATCCCGAAATTGGTCAGACTAATGCGAGTCGACATTTCGCCTGGCGCCCAATCAAGTGTTGCCAAATTGGGTTAGCCGAGGGTCCAACTAAAAAAAAAAAAGAGGAGACCGAGACCCAGAACCGTTCGTGGAAGGGTCCCGAAATAAGGCTTCCAGGAAGCCCTAGCACCAAACACACGGCGATGCCAACTTGCTGTGGAGATCTTCGAAACTAAATCGTATCGGAAATATTCGAAAATGTTGTCCGTCAAATCGGATTTGAGTTGAGCAGGTCAGTTAATATTACTAACTGCGTTTTTATCCTTAAAGTGTTAGTTTATTAATCAATTTTGATATATTCATTCAAAAATACAGCCAAAATTAAATTAACACATATACCTGACAACATTTTTAAGTGATTAAAATGTATATTTTCAATTTTCACTTTTACCATTCACACCACCAAAAAAGCCATCACAGAGCTGCTAACTTACTTGAAATATCCTTCAAATTCCTTTAAATCCTTTTCCAAAGGCAGTTTAAACTTTTATGTGCCTGTGTTTCCCCAAATTGCAGTTAAGTAATCACAAAAATGCCAACTTGTTTTCGGAACACAAAAGAAGTGCTCACATATTTATTTATATTTGGTTGTCTGCAGTGAAAAGCGAGTCCTGATGCGCCGCCCTCGCTGAGAAAACTCACTCAAAAGCGGCCCAAGCGTGCGAGCGAGAGCGCAGCGCTGCTGGACGGCGATTGGCTGTTTCCGGCAGAGGAAACTCACTCAAAACTCGCATGGCGACGACGTCAGCTTTGCGATCGAGAATCCAGCGATGAGCGCTTTTCGACCAAGTCGAATCAGTTGA

-1171 to +50 (1221bp) Composite Enhancer in Promoterless Vector

AAATATTTGGATATGTGGGGGAAAGGGAGTGCCCCGGGCTACGGTGGGGGGGGGGGGGGGTTCTTTGGTGCGGGGGCTGGTGTGTTGTTAAGCCTAACTGTTACTGTCGCAATTCATTGAATTTGCAGGGCTAATTAAACACGTTAACAACTGTCGCGATGCGCTGAGCCGTGCAGGGATAACTTGCTCCAAGATCGTTGTCGTCGTCGTCGTTCAGACGCCAGTCGACAAAGAAAAAACACAAAGAGGCAGCAAAAGAGGAGACGAAACTGGCCGTATGTCTCCTAGTCTGCCCTGTGATTTTTGGCTAAAGACCAGCGGCTTGGAGCTGTCCGCAATATCCCGAAATTGGTCAGACTAATGCGAGTCGACATTTCGCCTGGCGCCCAATCAAGTGTTGCCAAATTGGGTTAGCCGAGGGTCCAACTAAAAAAAAAAAAGAGGAGACCGAGACCCAGAACCGTTCGTGGAAGGGTCCCGAAATAAGGCTTCCAGGAAGCCCTAGCACCAAACACACGGCGATGCCAACTTGCTGTGGAGATCTTCGAAACTAAATCGTATCGGAAATATTCGAAAATGTTGTCCGTCAAATCGGATTTGAGTTGAGCAGGTCAGTTAATATTACTAACTGCGTTTTTATCCTTAAAGTGTTAGTTTATTAATCAATTTTGATATATTCATTCAAAAATACAGCCAAAATTAAATTAACACATATACCTGACAACATTTTTAAGTGATTAAAATGTATATTTTCAATTTTCACTTTTACCATTCACACCACCAAAAAAGCCATCACAGAGCTGCTAACTTACTTGAAATATCCTTCAAATTCCTTTAAATCCTTTTCCAAAGGCAGTTTAAACTTTTATGTGCCTGTGTTTCCCCAAATTGCAGTTAAGTAATCACAAAAATGCCAACTTGTTTTCGGAACACAAAAGAAGTGCTCACATATTTATTTATATTTGGTTGTCTGCAGTGAAAAGCGAGTCCTGATGCGCCGCCCTCGCTGAGAAAACTCACTCAAAAGCGGCCCAAGCGTGCGAGCGAGAGCGCAGCGCTGCTGGACGGCGATTGGCTGTTTCCGGCAGAGGAAACTCACTCAAAACTCGCATGGCGACGACGTCAGCTTTGCGATCGAGAATCCAGCGATGAGCGCTTTTCGACCAAGTCGAATCAGTTGACACGCAGCTTTATGTGACATTCGTGGCGAAAGCGGACCAC

+529 to +4272 (3744bp) Intron 1-1

GTAAGTTGAAAGATCTCAATTAGCTAACCGAATATCTGTGGAAGTTTTAGTATGTTGGGTTTTGAATGGATGTATATCTCATGTATTTACGGCAGCTACAAATTCCTGGACCACACGCATATTGCATAACTCGCACAAAGTATATTTCACCTCAAATTAAAACCCGAAGAAAGAAGCAAAATCCTAGTCAATTCCTAGAGCTATTCAAACCTGACTCCTGGTGCTCTCAATCATATTTTCCACATCAAGTGCGCATAATTGCTATCCTTTAATGCGTTCCTCTAGCTCCGAAATGGCCAAAATGGTAGCAGTTGATCGTGAGAGATAAGCTCACTGTACAGTGAACTGTAAGAAATCAAAGATGCTACTACGCATTTGCCCAGTGCTGATCCTTTAAATGCAGGACACAATGCAGACAGTAGTCAAAAGGGAATTTTCAGCTTAAGTGCGCACAAAGGGCCACAGTCAGATATCCAGATATCCTGTCGATCGAGAAGGAATCATTTGACCAGAAATAAAACCGCAAAAAGGATCAGTTTGAGAAGGCACTAGGTCAAGGGCTGTAACTCAATGGAAGGTAGAGATCTTTAGATCGGAATTTCTGCTGAATCCCGCTTTGATTGACAGTTGTACGCTCATTTCCTATCCTTCTCTGCTTCTCTGTTAAGTGAGTTCCATTCCACATAATCGTGCCCTCGTTGCCAGACAGCTCCCTCACAACTCCGGCGTAACTTGAGCAAACTTTTCGGGCAAAGCAATTTGCGATGTGTTTGTCTCGTTGCCAAGGCAAACAGAACACGGCTTTTTCCTGCCAGCGGCCATCCAAAATGGCTACCGTCGATGGCGGCCGGAAGGAAGAAGCAGAAGAAGGCAGAAAGCCAAAAACGTCAGAGATACAAAAGCGTCAACTGCCGCCATTTTTTTTCCGGGTATCTATTTGACAGCATCGGAGACAACACGAAAATCTCATAAATGGGAATGCAAAAAAGGACGAGCCATTAGCAAATAATTTCAGCGAAAAACATTTTATTCGGCATTGTTTGGCGAGCTGGAGGGAAAAAGACGGCTACACACGGTGAGTGAGTGAGAGAGAGAGAGAGAGAGGGAGAGGACAGGCAAATGCCGTGAGGATAATTTCATTGAAATTTGAGTAGTCAGAAAGGAGCAGCAGAGAATGTACAAAAGGACAACGCCGTGCAGATGCCTTGCGCTTTTCGTTTTGCCATTAAAACAACAAACAATGCAGGCAGGAGCGGGACAGGATCAGGGGTTTCAACTGGGGGTCCTTCAGCACCTCATCCCCCTCATCACCGTTTCGGGATTTTGCGCTACCCGCAGGACTTCTCGCTCTGCGGGTTCCCAACGATAGCTAGCATTTCAATTCGTTTCTCGTTTGTGGCCGGGCTGCTCCATTTCAGCCACGCCCTGGGAATGTCCCAAAAATTAAAATTAAATGCAAGGGGGTGGCCTATTCGCTTGAAGCTCATCACATTATTTCCCCAGGCTCAAATGCCTGGCGACATTTTTACAAAATAATCTGTGAATCTGTGAGTCCAGGCATTACATCTCTCATCTCCCAGCTCTTAATCCGCATTGTCCTTCGGCTTAAATGGCAAATGTTTGTCTGGGGCGTGGCACAGGAAGTGCAACCATCTCTGTCTGGAAGGGATATACATACACCTACATACCTGTTCCCGTTCGAAGTCGAGGAATTTATTTGGGTTTCTGCCACTTCAACAAATGAACTTGTTTGTTTGTTTACTCAAAAGTTTCGCCAGCGCTGAGTTGGGCGCGATTTCAGTAGCCATGGTCGAGACAAAGTCAATAAACAAACTTGAAGCTCGAATTGGAAACTCTTCTTTGGATACTGGCACGAGTTCAATTCGATGATGATCTGCTTGATCATTTCGATTTATTGTACGGGGATTTATGGTGATATAGAATACTGTAGAAGAATAAGTTTCCTTTTCCAAATCAACTTAGCTATATATAGCTAAGCTATATTATATTATTATTATATTATATTATATTAGCTATATCACTTCCTGTAGTTTTTGTCAAAATTAATTTAAAAACTTGCTCATGCTTATCGCTCGCCTTTCGCCTGACATAATTCCATTGACATCAGCCGTGAGTCCTTGCTGTGACGTCATATCCGAAGTCTAAACCGCAGAATGTATGGCATAAATTTGCATGCATCATTCTTGAATTACAGCACTGGAAAAAGGGCGAGAAATGGAGAGGGTAGGAGGAGGAGGAGGCGGTTTATCCCTTAGGCTCCTGCTCGAAGGACCTGCTTTTTGTTCCAGTTTCACCAGAGGCTCGCATAATCCGCTCAACTGGCGACGCTGCTTAGTCCTTTTATCCTGTTTTATGTCGCCTGTCAGCAGATGAAGACGTGGAACGGAAAATTAGAGCAACCTGCCTGATGGATGGATAAATGTCCCTTCCTTTCCGCTGGATTCCGATCCTAATGCCCTCTAATGATTACAAATATTTGTACCTTGCTGTGGCCATTTTAAGAGGGATTTGCTTTGTCTGCTCTCTCCAGCCTTGTGACTCATTGTTGTCTCCTGGTCTAAATTTGAATGGTGGCAGATACAGAAATAGCCCAGTCAAATGCGAAACGTCTGTACGCAATCAATTGTCACTTTCGACAGCATATTTTGAGCCAACGTGCGTCTATAGATAGCCATCACTACACTAAATATAGTGCCCAACTCTTTCCTGGCCACCAATTTGGTGAATAATTTGGAATAGATCACCAAAACATTGACAGTGGGGCAACATCTAATGGTTGTCAATTAGAGCTAAACATCATTGTTGAATAAGTAAAATGAATTACAAGGGGATTTGCCAATAAAAACGGTTTAGCTTAACTTTAAACTATGACAAACTATTTGTCTCAAATCCCCCTTTTCAAATTCACAGCTTACTTTTGAGAAGCTTCTTGAAAGCGTCTTTCAGCCCGTTTGTGGCTAAATCGAGAAGTATTATTCGTAGAACCACCATGTGATGGAGCAATACACCTCAGTAGCGACACGGACACGCATCTGTCACATCAAAAGCCGCGGGCATCTTACAACGCTCCGGTAATTGGTGGGGCCGGATCCGGGTCTGAGTCTTTTTCCGTGGCTGCCTTCGCTTACAGGAATTCTTGGTAAAATCGAAGGGAAAAATAGATGCGAACGGGAGCCAAACAAATGAGCGAAAGTGCGACGAAATGAACTTGTCATAATTATTTGACAGTCAGGGTAAGTGGGATGCTGACTCTGCCCAGCGGCACATTAACCAAAAATTGATGTAAAACATTTTGCCGATGGGAAAGGGGTGCCATCGCCATTGGCATCGGCATCGGATTCGGTTCACTTTGATTCCACGTTTCGGTGTCACTGCCAGCGGGTTGCGGGGTCAAGTGGTCGCAGCCTGTGAAAACAAAACTTGCGCCTCATTTCCGGTTCAATCAAGTGGCAGACAGCTTGGAGCATGATAAGACTCCTGCCGGCGACTGTGACTCTCTGCGCCCGGTTTACTTAGGTGTGGCTGTTGTCAGTCAAAGGCGCAGACAGCCAGCTGCCCTCCTTGTAACCCCATCCCATTCCAAGCGATCCCATCCCACCCAAAGCCCCTACCAATTAAGCGGTTTTTTCCGAGACAAATTTGTGCGACCTTTGACTTTACGGAGCGAAATGAGAGGAACGGGGATAGCATTACTCGTTAGCAGTCGTGACAGTTTTTCTTAGA

+3957 to +7672 (3698bp) Intron 1-2

GTGAAAACAAAACTTGCGCCTCATTTCCGGTTCAATCAAGTGGCAGACAGCTTGGAGCATGATAAGACTCCTGCCGGCGACTGTGACTCTCCGCGCCCGGTTTACTTAGGTGTGGCTGTTGTCAGTCAAAGGCGCAGACAGCCAGCTGCCCTCCTTGTAACCCCATCCCATTCCAAGCGATCCCATCCCACCCAAAGCCCCTACCAATTAAGCGGTTTTTTCCGAGACAAATTTGTGCGACCTTTGACTTTACGGAGCGAAATGAGAGGAACGGGGATAGCATTACTCGTTAGCAGTCGTGACAGTTTTTCTTAGATTTTTTCATAATTTTACCACGAATTTTTTCCTCGCCTGGTCTCGTCCCCAGCAATTTTCCAGGCAGTCTCACACAAAAAAAAAAAAATATGAAAAAAAAATATCAGATTTCCTCGTTGCACATTTTTTCCATCCATTCTTCGGGGCTTGGGAAAGTGCAGCAGCAGTAGCAGCCTCCGAAATCCGGACAATTGGCAGCCAAAATGCCAACAAACAAATCATTCACATTTCGTCCATTGATTGGCGAGGCGGTAAAGCGAAATTCGATATTCGGGATCGTCTGGATGGTCCGGAGGGAGGGAGGAGAGAGGTGGTAGATGAAAGTGCTGGCAGGGATGCAGCGGCAAGGGTCCAGGGGCTTAGATCCCGGCACCATCCTCCGATTTCCATAGGGTTTCCGCACCGATTCCGGTTCGATTCCCAGTGACGAGCGCACCTGTTCCTCCACCGTCGCGAAAGTTGTCCCATCAACAGTTGGTCGCACAATTCAGCTTTACGCTCTTCCAACCAAACGGGTAATACTATACGTATTTCCCTCAATTCGGTCCGGCCTTGCCGCTCTATTTTCGACGTTATTAAGTTTAACGGAAATAATCAACTATTTGCCATTTTTTATACCCATTTATAATAGTTAGAATTTCCACAATTGTCGAACGGCTGCCGCGCAAAAGTTACAATAAATGTGGCCAAGGTCTGAGTGATGAAAATGATACGCGGCAACTTTAGGGCTGCCACATATGGTGTAAGTGTAACTGGTAGTTCAGCAGGGTATTCGGTTGCCGTTTACCATTTTACCAGATACCAGTTGCCCAAGTCCAAGTGTTGCACAGTTGGCACAGTTTGCGGTGCGCCATCAGCCGCAGTGTGGAATCTCTGCCTTAAACTCGGTAGATTCATCGCAGAATTAACTTAAAGATTATTTTATTAAGGGATGAAATAGAAAGGCTAACAATACTGAGAAAAAAAAGAGTTATTCATTTATTTTCCCAAAAGTACCCTGTAATCCGAAAGACATCGTTAGCGCTCGCCCAATCTCAACTCAACTTTATAATTTACAATACTGTCTTTCCTTCAACTTAATTATTACAAGAATGAAAGGGTTGAAGCGCAACTCAAAGGGTTAACAAGCAACTTCTTCAGCAGATAAACTCAAAGGGTTAGCAAGGCGAACTTCTTTAAGGGTTGTTCTTCGCCTAGCAATCAAAAGTTTTTAATTAACAAACAATGCAACCGATTTTCCGAAAAAGTAGTGTAAATAATTTGCAATGATTTTTTAACCCAAAAGGAAGTTTTCATTGAACCCGATCCGTGACATCTTGAGAATAGGTCAACATTGTTGTTTAATTAGAAGACAATAGCTGTCAATAGCTTATTCCGGACAGTCAGGATTCCGGCTCACCATGAAGGGAGTTGGGCCGGAGTCAAAGGTTGTTGGCTAATTGCAAGGCGGGGTTCTTGTTCTTGCTTTTTTTTTCGTTTGGCGAGGGGTTGGCGAATGTACTGGCTGGTACCAACGGCAGGACAGCCAGCTGTGGAAAAATCTTATCCACACTCGATCTTAATGACTACTTAAACAGCGACAAAGCCAACGCCCAACCCCGCTCAACCCCCGGGTTGCAACCCCCTGTTCCGGGATCCAGATCGGCTCAAGTGCGGCACTTGAGGCAGCTGTTTGGCACTCGGGATGACTTTAATTTAAGCCCTGGGGTGCCATATATATGGAAACCTCAACATTAATAAGTGTTAATAATTGTGCGTAAGCGTGTGTGTGTGTGTTTTTTTTTCCAACTGCGTTGACACGCCCCCAACCGCCCGAAAGCAAGTCTATAATTTCACATCACTTTCGCCACTGTTTTTGTGGGGGAGTGATGGGCGGCTGGTTGGGAGGACAGAACCGAGCCGGCGAACTGAGACCGAGTTAGAAAGTCCGTCCAGAGGAGCGTAACATGGATAGGGCATGGGTATTGGATTACCAGCGTCCCATGGCATTCGCATCCACCTCCGTGTCGGGCGGCGTCGTCCTCCTCCTCCTGCTGACTCCGGAATCCGGATGGTTATGGCAACGGACGGATCAGCAACCGAAACAGCATCCCTGGGGGTCTCCAATGATTTGTTGTTGCCCGCGGACAAGTTTACAAATTCAATTTGTCAGCGATGCCTCAATGTTGTTGTTTTCCGGACGGGGCGAGTGGTTGCAAGCACCATTTCATAGGCCTGGCAACGGTTAACCCAAGAAACAACGCAGCCAAATTGGCGGAAAGTGCGAAGGGCGTCGACTACAGCATACCCAGTAAGCTGGATTCTGTGATCCCTAGGGCATCATACAAAAGACGAACTTATCTAGTACATAAATAAAAGATTTAAGAACGCATTTGATTTAATTTGAGAGTTTCAGAAGATTATCATATACCCTGGAGCTTACTAACAATACTTTTTTAGTTTATAATTTATAATTCAATTCAATATATCAACAGGAAGAAATCTGTCACTACTTATCTGCCTTGGGTACCATTGTAAAATGCTAGCGATTTTGTGCTGGCATTCCAAAGCCACCCCTAGTCGAGCACACCCTCTTACCCGATACCCTGTGTTACCCACCCGGTAAAAGCATATGTTTCCATTGCATACTTGAGGGCGCATTTGATTAGAGCCCACCGAGGCGTGAGTATTTAATTTTGTTGTTCGTTGTTGGGTCGCTGCGCGTTTTGACACGCGTTAGTCGAGAGCACGTAATTAAATTAATTAGAGAGCCGTTCCCACAGGTAAACCTCCACATCCACCCACTCACCCATCCAACACAATTCGCTTTGGTCCAATCCAATCCATTCTGCCACCATTCTGCTGTTCTGTAGTTCTGCCGTTCTGCGGCAGTTTTGATGCTCATTCGCAGTGACAGGCGACACACTTCGCTCGGTGGGTGGCCCAGTCGGCGGCAGGTTGATGGGTGGTTGGCTGGGTGGCGTTGTGCGCCGGTTGCCAGGAGAACACACACATAGAAGCTGCTCGGCTTTGGGCTTCGGCTTTACCTTTTAATGAAATTCCTAACCAATGTTTGCTTCGCCTCGGTCACTATGGCGATTCACATCCTTTCTCCACCTCCTCCTGACCCCCATCAGATGTGCATACTTATGTACATATACGTATCAGTCGGAGATCGGCACGTGCCTGGAACTGAAACACAGGTTCCGCATAATTTTTCCCAAGAATGAAAGCATAAACGAACCGTTTCTCGCCGACCCTGGTGGCCATAATAATCGGCTCAATCATAATGAACTGGATGCCTCATTCTGGTGCAAGTGCAATTCCCGACTCAGTTTATTTTTGTATTCATTGTTTTTTTGTTTTGCTTTTTATCGGGTGTATCAAGACCCAGCCAAACGCCC

+7330 to +11076 (3747bp) Enhancer 3 Large Fragment

TTTGCTTCGCCTCGGTCACTATGGCGATTCACATCCTACTCCGTTCCATTCTCCACCTCCTCCTGACCCCCATCAGATGTGCATACTTATGTACATATACGTATCAGTCGGAGATCGGCACGTGCCTGGAACTGAAACACAGGTTCCGCATAATTTTTCCCAAGAATGAAAGCATAAACGAACCGTTTCTCGCCGACCCTGGTGGCCATAATAATCGGCTCAATCATAATGAACTGGATGCCTCATTCTGGTGCAAGTGCAATTCCCGACTCGGTTTATTTTTGTATTCATTGTTTTTTTGTTTTGCTTTTTATCGGGTGTATCAAGACCCAGCCAAACGCCCACATACTCGTAGACCACTGACCAAGTCTTTGTTTAGCTTTGCCATCCGATCGACGGCTCCCTCTGGCCTTGTTGATTTATAAAATCCAAGTTTATGTTCATATTTTTTATAGAGCCAGCACAGTAGTTCGTTTTAATGATCTTCGACCCATAAAATGGCCTGTCAAAATTATATATTCGGCTAATGCGCCTTGTGCTCAGTTGTATAGGAAACACGATTTTTGTGTGATTTACGATCGATTTGAGGCGAGTCGATTCGATTGATATGGGATCCCTGCTGCAACAGCTGATCGTCTGGAGTTACCCTATAAATATTTGTACAAGTGGGAAATGTGTTTTCCATAGGAAATGCAATACCTTCAGATTGGCTGTTGACTTTTCCATTTATTTCGCAAGTTTCTGTGGAAATAAACGAGTGACAAGCGGACCAAGGAAAGTTCAGTGCGCTTTTGTCTATGAAGCGTTTTAATTACCAAGCCGTTTCATTCAAGATTTTTAGGAATTGAAATCCCATGTGCAGTTCCCCTTCGGAAGACCAGCTACAAAACGAAAAAACCCCAATCCAGAAATCTGCATACCAAACCTCTTCTGGGCATTACTTCATGCAAATATCGCTGCAATTTTTAGCTCCATGGCCTGCGCTTTTTATCGTTTTGTTCTCTATTTTATAATATATGCGGTCAGATCTCCATGGCTCAGCAACATTTTTTTCGAGGGGCAGAACGATGAATGCCTCAGACGCTTGGCATGTGCTGACAAATGTGAAACTTCCCTAATCAGCATGGCATGGAAGAACTGACCCAATTCGTGGGCTCGCCAATCGAATGACGCATTCCTCGACGACTTTTGAACCGGAGAACCTGAGCTGCGTCGCGAAGGGGGTGGAGGGGCAGGCCACACTGGGCATCCAACCAAATGACAACGGACAAGCAATTCAATGTCCCAATGCTGGCGTAGCCAAATCCAAGTCCTTATCTAGATGTCCGGCGAACAGTCGCCCAGATCAAGTTGTAAGAATGCGAACATGGCAACAAATGTGCTCGACTCAGGTGTTAAAACTTTTGCATTAAAATGGCGCCCAACGCGGAACGGACACAGGCCTCTCGATCCCAAACCATGTGGCCCCCACTTCCTTTGGCTGTGTAAAATTAATACTACACTCGGGGAGGTCCAGCCCGATCAAAACTGAACGTAAATCTTAAACACTTTCGAGTTCTACCCTGCCCCCGTGCGATTCTCTGATTCTCCGATTCACCGACTCCCTGATTCTCCATTGTGCAGCAGGCTTTTGCAGTCGCACGATCAGCACGTTCAGTTCCGAGGGGATCTGCTGCTTCAAGGGGGAAACTGGGATGCTGGATGGTGGATGGTGGATGGTCGACGATCGGCAACCGGTCCACGACAAATGTGCCAGTCTCAGCAACAAGCCAGGCCGCAGAAATATCTGCAAATGCAGCGAATTGTGCGCAGATTTGTTTACAGAAGACTCTCCGAGTGGCAAACTGAGGGAAGACGGATTATCGTGGAGGGAGGAGCACCATGATCATCGTAGATATAATTGACGACATGTTGTCTCACGATCGTGCTAAAGCGATTAAAGCTAATTTAATGCCCGACTCGGAGATTCACTGCACCCAAAATGTATCCACCAAGAAGTGCACTCCCTCACTTTGAGGTCATGCTCGATTTTCTCTGTGGAAAGCCTTAACAAAATGCCATAATCAAATTTCTAGCTTCACTTTTGTTATTTCTTGTGGCTGAATTTGTTGTGGGATCGAATGGGCTCATTGTCGGAATTCCATTGCAATTCTATTGCATGTGTGCATGTCGCCTATTTACTTAAATGCACTTGTTTCACTTGCTCACTTGTAATTACATCCAGGCTTTTAAGTCAGTCTCTGTTGGGCCAAAAATCAAGTGTTGAATCAACATGAATTGTTTAGCACACGATTCAATTGTTTCGTCTCGACGGAGACGTATAAATAAATAAATACGAGACGGCCATGAAATATATATTTATTAGAATAAAAAATGTTAACCGCTATCGGTTTATGACAACTGTTTGTTTAATTGTAAAATATATATTCAGGAGAGGTTTAAAAGATATTTTACAAGTTGCCTACCTTAAAGCTTAACTCTTTTACTAGAATTTCATTTGGATGAAGTTTCAACACACTTTAAAATTTATGTGCTCAGTAATTTGCCAGAGCTGCCCCAATCAACATCAATTGGCTGCCATACTGTCTGAGCATTCTAATTCATTTAATCGAGCTCATTAGTGGCTCTAAACCATCATGTTTATGCTACCCAATTACCATCGGAAGTGATTCACTTCTGCATGGACGGAGAGATAGCCCTGTGATTTTGATTAGATGCCAATTTGTTTATGGCACTCACTAATCCGTCCACAGATCGGATATCCCATATACCATATACCATTTGCCATATTCCATATACCATGTCCCGTATCGGTTTGATTATGCCCAGCACACTTCGGCTCGTCTTACATGAGCATTTCGATTTTTGGGAGCTCCTTCTCTAATGCGTTACGGATCCATCGAACCCAATAAGTTTTAGGCGTTTTAAATTACCCGATATTCGCGTTGCGCGTTTTACGATTGCCATTAAATTTGCTGGCGGTTACCATTGAACAGGCCGAGAAACTCCAACTGATTAGCTTATTAATTTGTTTTATGTGCTCGAATGGTGGCGGAGTTGTTTCGAATTAATTTATTAATTTATGCGCATAGGAAAACCAACTTTTCAGTTGGCGTTCGCCCAGCGGCAAAATCTCATTCACAATGGGCATTGTTCGCCCGCATCCGAGAGATACTTACCAAAAAAAAAAACTAACATAATACAGAGAAAGAAACAACAAACTTTGAGGAAAAGTGGTTTTCGACCCACCCCTGTGCGATATTCTTGGCCCTTCGATTTGGGGAATGGGAATGGGCGGAGTCTAGGCGCATTCTCTGGCCGGCGGAGGGAGCGAGAGAGGCTGCTCCGAGTGCGCACCGCTCACACGAACTCGTTTCGAGTGCCGAGATACATTTAGCCGGTACGAATACGAATCCAAATAATCCGAGCCAGGGATTTCCACTCAAGCAGCGCCGCTGCCGCTTCGCTGCCGGCGTCGCATTGAGCCACAAAAGTCGGCTGTTTCGGTGGTTCAGTCGATTCGAGCCACTCAAAGCGAGCGCTACGGGAACGGTCGATCCGCCCGAAGTCGCAGATAAAAAACCTACCAGATACATTTCGTTCGTTCTGAAACGCTATAACTAAATATATATTCGATTTCAAAACATCGACCATACATTAACTACCTGAAACGGTCGAGTTCACTACCCCGCCACGCGTGTGTGTTTTTGTGTGTGTTGCAAGTGAAAGTAATCGCAAGTCCACAGA

+10576 to +11076 (500bp) Enhancer 3 Minimal Fragment

CACCCCTGTGCGATATACTTGGCCCTTCGATTTGGGGAATGGGAATGGGCGGAGTCTAGGCGCATTCTCTGGCCGGCGGAGGGAGCGAGAGAGGCTGCTCCGAGTGCGCACCGCTCACACGAACTCGTTTCGAGTGCCGAGATACATTTAGCCGGTACGAATACGAATCCAAATAATCCGAGCCAGGGATTTCCACTCAAGCAGCGCCGCTGCCGCTTCGCTGCCGGCGTCGCATTGAGCCACAAAAGTCGGCTGTTTCGGTGGTTCAGTCGATTCGAGCTACTCAAAGCGAGCGCTACGGGAACGGTCGATCCGCCCGAAGTCGCAGATAAAAAACCTACCAGATACATTTCGTTCGTTCTGAAACGCTATAACTAAATATATATTCGATTTCAAAACATCGACTATACATTAACTACCTGAAACGGTCGAGTTCACTACCCCGCCACGCGTGTGTGTTTTTGTGTGTGTTGCAAGTGAAAGTAATCGCAAGTCCACAGG

+11149 to 14024 (2872bp) Intron 1-4

GTAATCACATAGTCGTAGTGCTCTCCTCGCGAAGCTTTAAAAGTGATGTGGTTGAGACGGCTGTGCCGTCGGTTATTACTATTACTAGTATTGCTAGTGTTATGACTATGATGACGGCGGTAGCTTCCTTGGTGGTTGCGCCTTGTGGGGCTTCACCTAGTCGGGGAGTAGTATCCAAAAAGTGAAACTCCATGGCGAGATAACGCTTCGACTCTCATTTATCAAGCCTGGATGGAGTCTAATATATGTACGAACAACAGTTTAGGATGCTTTAAGCTGATCTATTGGCCGCCGTGATATCATAGCACACTTCATATATAAATCTAGTCCAGCTTGACAACTAAGTCGTGTTCAGATGCTATTCGATTTCATAAATCTAAGCGAATAAATCGTGCAAATACACTCGAAGAAATACAATTTCATTGGGTTGCTTGAAAATGTATGGAACGTTACTCATTCACAGAGGATTATCAATTTTATAGACCCATCCTGCGAAGAAAAGCGCTTTAATGACTGCTCATTGTTGGGAAATCGTTAATGGGGATAAAGATTATGGGCACCTGACCAAAATTTTATTACAACGCATTAATCGGCTTAACTTTTGGCTGTGGGGTAATGAAGCAAACAAGTTGAGTCCCAAAAGGTCATTAAAAACGCTTAATAAAGTGCCGAAATAGTGACAAACGTTTTTCCAATTCGCTGCTGATGCATTTCTCCCATCAAATTACCAAAGCCTTTTCATTCACTGCACGCTTGCGATGCAAAGTCTGGCCGAGATTAGCGTAAAGTTCGTTTCCCGACGATTTAATGGAACGGCATTCTCCAGGCTTTTATGGGTCCCTAAACTACGGCTAAGACAAAGTCGGATCGAAGGAAACGTGACGAACGTGGAGTGGCCGGATGGCCGGATGGTTAAGGCGAAATATTAATAGAGCATAAATTACAGCTCTAGTGGGTCTCTGGGTGCCGGCTTTATGGGAATTTAAAGAGCCTGGCCCACAAAAGCCAGAGCTGGGCTACGGCATTCGTCCAGCGGCATTAATCACATTTTAATTAAGTTTAAGCCGTAATCGTAAAAATTAACACGCCATAATTCGACTGCGACAATACGCGCTGCATGTAATTTATGACAAAGAGAATTCACATCGGTGTGCGTGTGTGCGAGTGTGTGTGGGGGGGGGGGGGGCGGTGGAGGAGTCGAAATATTGATTATGGCCACTGCGATTGACACTTGGCGATTGTTGATTATACTACCACGATCGTGGCCCAAAACGTAATTAACGCGCCAAAGCGTTAAGGTGCATGGGATAGGTCCCATGCCATAGCAGCCCCTTCCTTCGATTCCACTTCGTCCACCAGAAGATACAAATTTTCCTCTTGTTTATTTTGATTTTTATCGCGGGGACTCGAGGGGGACTCAAGGGGTTTGTAGGGGTCTTCGGGGTCTTAGGGCCGGGTTAATAAAAATTTGTCGCCGTCTTCTTGTCTTGTCTTTTGTTTGCCTGCCTTTTTGTGACCGAGTGTGTGTGTTTTAATCTTCCTCTAAAGCAGCAGAAGAGACAAAGAGATTACGATCACAGGAATTTCGTTAAAGATGCCCACAAACAGGTCTTTATACCACATATCTCCCACTCTTTTCCGCAGCTGGAACCAACCAAGCCGGCCCAACCAAAACCCCCCCAGATACTGATTCGAGTGTTGTCTGTGTTAATGTTTACGTTGTCAGCTTGTGCCATAAATTAGTTTAGTAAAAGGTTGCATTTGACTCTTCCCTTTCCCCCCCCCCCCCTTTTTTTTGTTGGCCCCTCACCAGCTCACTTTTGCATACAACTTCGGACTCGAATTCGGTTGCTCGTGCAGCACGCAATCCTGCAGAATTCGCGACCCGAGATCGCAACTCCAATGCGCAGCTCATCGAAACGCTACCGTTCGGCAATCTGTCCGCTTGTCCGCCGCTGCGCATGCGCGTGTTCCCTTGGTCCGTCGACAAATCTGAGCAAACTGCGTCCTGCTGCCCCCCACCAGATCACCGGATGATGGACAGTGCGTGTGTGTGGTCATCTCGCCGGACGGCAGAAGCTGTCAACCGAAGCCGCTCAGCTTTCGCTCCCTTCCCGCCCTTCTTTTTTTGTTTTTTTTTTTTGTTTTGAGGGACTTCTTTAGGGGCGGAGAGGCGCACAAATTCCAGCACCTGGGCTTGCCGCGCCCCTTAGTCCGCCTTCGATTTATCTGGCTTCCGTTCGATTTGCATACATGTCTTCTTAAGGAGCGTTTTTTTTTTGACCATTTGTCAAAATCATTCTGGCAAAAGCTGAATTGTTAAAGCTTTATTTTTATAAAACCTAGTAAGTGGATGTAAAGGACGTTCTATTTTATGCCATATCTAGAGACACAATCTTTCCTCGAATGTCTGTATACTTAAATGTCATCTTTGTTCCATCTAGCCATTTTGCATCCTTATCGAAATCCGCTGCCACCTGCTGCTTTTCCGGGCACTACCACCTCCCCCCCCCCTTTCCGACTTCCCCTCCAAATGGCGCCGCACATCCGAGTAACAGTTGAGTTAAACTTTTTCCCACGCACAAGCTCGGGCAGCGAAAATCGCTTGGCGCAGCAGCAAATGGCGATTGCGAATTATGTAAAATTTGCTGTTTTCTTTCGCCACTCGCACTGGATAACCCAAATAGAGTTTCAGGCGGTGGAAGTGCCGAGCTCAGCAGGGCGATTCCACGCCAGACGCTGAGGTGTGAGTGCGTCCGCCTTGTTTATAAACAAATTCTTTCCAGGGCCGGCGAAAATCCAAAAAAAATCACAAAAATTAACATTCAGTATAAGTATATATTTTAATGTAATTTTCTTCGCTTTTTTTCAG

+14072 to +16647 (2576bp) Intron 2

GTAGGTTCAAATAAAATAAAATATATATAAATATACTTATTCATAAACATGTAATTTAGGTACTTTTATGAAATAGATTTCTTAAAAGTGTAATATGTTCTAACTAAAACACATATAGGGGAAATAATAGTTTGCATATATTTATAGGTTGAAACCTAAATTATGAGTCTTTTACCAACTTGCTTCCCAGGATCCTGAAGAAAAAGGTTTCCCTCTCTGTAAGCGTAGACAATTTGTAGCAGAACGGCGAGAACATCCTAACACTTGACAAGAGGCTCACCATTTTCAATTGGTTTCGATTGAAAAGTGGCCATGGTCGGATCGTCGTAAGGGAAGGGAAGTGCACATTTTGCATGTGGTCCACACAACCTGCCACATGCCCCGGCAGAAATTCAAGGAATTCGGTGCACGCTTCCGCTGCGACACTGAAGCGAACCCAAGACCGAAATCCAAAATCCAAAAACCGAAATCCGAAAAACAAAAAGAACCGAAAAGTGTCAGGGCACTTAACACTTGCCAAGGGGCATGGTGGACATCGTAGGGACACGAGGCGGTGCAGGGCCAACTACTTAAAAGAGGCTTAAAGGCGGCAACAGTTGCGGCTTAGTGCTAAAGACCTCTCAACCTCCCTCTTCGATAATTTCTATTATAATATTCGTAAATCTCCCATTGGTCAGCCCATATTGTGAGGAGAGCAGATCTCAAAGCCAAAGTTTGGGTTGGGTTTCCTCCCTCCATTTGGCGCACGCGTCGTTCTGCGCTGGGAAAATGATTTTCCCCCTTTCACCAGCTGCTCCCCTTTTACCATTTTCCCTCTTGTTGTGGCTGCCAGCAAGATAAATGTGGCCCCTTTCCAGCCGAGCGAAAGAATCTCAAGGTGTCAGATTCCTTTGGAAATTGGGCACTTAATTTACCTTCCTATTAAAATTATGAAAAATTGTTAAATATTTTCCAAGCCTTATGGGGGGATGGGGGGAAATCGTAGGCGGAAAAAGTGGAAATATGGTATACGGTATAGTCGAAGGAAGAAAGTGAGGCAGATAAATCATTTGAGTAGAGAGTAATTTAAAATAATTATCCTCCATAGGGAAATAACGCGGTGTTTAAATGATTTTATCTTGCTACTTTAAGATAGAAAATATGGGATTTAATTTGCCAGCTGATTGTAAAAAACTCATCTTAATTGCATCGAAAAGGATTGAATTTCTCCCCCAATTTGTCTCCATTTTTCCCCCAAAATATCGAAAAGCCTTTTCTTTGTTTTCATTTTCCCGTAGCTCTGATTTATTTGACTTTCGACTGCTGCTCAGTTTTATTTGATTTTTAGTGAGCCCTCGTAGATTTTCCAATTTGTCCACTTTAATAATGTCCTACTCGAAAAGCAATAACATCAGAGTAACAGAACAGAAAAAAAAACATCAAAACACAAATCGTTAAATATATGTATATAAAAGAAATATTTGTCGCTCAACATTTGCTGCACACATGGCCATGGCAAGGGAATGGGGGGGGGGATGGGTCAGGACTTGTCCGGGACACTCCGCACATGCCACCAAAAGGCGGACCCAGCGATCCGCTCAAGTGCCCCGCACCGATTCCAAGCGATTCCTCTTCGAGATCCCAGTAAACAACCCGAACAAATCCAGCAACAGGAAGAGGGAAAGAGAACGGCCAGATCGTAGAAAAATATTTATAACACTTGACAAGATTTGGAAGACCTCTTCGCTCGGGTCTTCGACGGGGAAGCACTTGACTTGGTCCAAGTGGCCCGAAGGGGATCGGGGAATGGGCAACCGAGAGCAGAACGACTACGTGCCGTTAATTTAATGGTTGTTTAAAGATTATTGGAAACTATTTTATGTGTTTCCTTAACGAGCAAAAGGTTCTGTGTTTCGGTTCTCCTCCACACAAAGAAAAAATCCTCATGTGTGTTTTGTGTACTATTTTATACTCAATCCTCAGTCGGCCTATTAATAATTACAAATCGTCTTGAAGTCGCTTGAAATTCATCAGTTTCCAATATTGAAGATATAATTACATATTTCTAGCTTTATTTTAGAGTATTAAAAGGGAATCATAATTTAATAATTTGTAATTTAATAACTAGTTCGCTTCGGGCTTTAGCTATTTATAAGCACAATTAAGCTGTAAGTACACGTTTTATTAAACAAATGAGCCTAAAATAAATACTTTGATTTAGCCGCCACCTTTTTGATGGCATATATAAATTAAATTAACCGGTTTTAATATAAATACCTCGATTGATATAATGCAGTCCCAGTGACTCGGTTATTACATTAGTCATGTTCGCCGCCTAAGGATATATTTCATTTGCTAAGAACTCGTTTTTAAATATTCATAAGCATAAATTGTAGAAATTATATAGTCAAGTTCGATTTAAATATCAAATGTGACATTACAATTTAAGTATTTCAGCTTTTGTTATTTGTGAATGAATTGTTTACATTTAAAACGGCTTAAAGGTCTGTGTTTCATTGTATATTCCCACACTTGACACCACCACCCCATTAATTGGCTCACCCACTTATGACGCCTCTCATCTTATCTTCTTGCAG

+16648 to +18402 (1749bp) Exon 3

CAATCTGTCACAGCAGCAGCAGCAACAGCAACCCCAGCAGCAACAGACGCATCAGCAGCAACAACAGCAGCAGCAGCAATCCCATCAGCAATCCCATTCCAGCACCGTGTTGGCCAGCAATGGACCCAGTAGCGCCGGTGCCGGCATGGGTGTCGGTGTGGGCGGAGGCGGTGGCAGTGGAGGAGGAGTAGGAGGCGGAGTTGGCCAGTGCAGTCCGCTGGGACTGCCGCCGCAGAGCCAGCCGCTCCAGCCGACAATAGGATCGCTGGCCTCGCTGAGCGGTCACTACTCGAACGGTAATGCCAATCCGAATGTGAACTCGAGCAGCTGCAGTCTGGCCACAGCATCCAGTTTTGCGCAGTCCGCCGGCAGCAGTTTCTCCACATATCAACAGGCTGGTGGCACCAGCGGTGGAGTTTCTGGCGAGGATGGCGTGGTGGGCGGAGCAACTGTGATGTCGCACTGGACGCACGATGGCACTGGCTCGAGTGCAGCGGTCAAGTCGGAGTCCCGCAGCCCGGGCCAAGTGCACGCATCGCTGGACAACGGCTCGGTGGCCGGATCCAATTTGTACGGCTGCAGCTCGGCCAGCAATCCGCTGGACGGAGGAGCAGTGGCGGTCAACTCTTCGGCAGTGGCAGCGGCAGCAGCAGCGGTCTACGACGGCAAACATGACTACTACTACTACAACAGCATGCAGCAGTACACGCCGCCGCCCTTCTACTCCGGATACGGAACTCCTTATGCGGCGGCAACGGCGGCACGGCAGGCCAAGATGGAACCCGGAGCGGCAGCTGCGGCGGCTGCCTACTTGACGCCCAGCTATGCCGCCAGCGGCAACAACAACTCGCAGCTGTACAGCAGTCCGTACGCCGGCTACAACAACTTCGGGCAGCAGGACTACGGCGGCTACTACAACGAGCAGTACGGCAACTATTACAGTCCGGCCAACTACTCACCGTATGCGGTCAGCTCGCCCAGCTCGAGTGCGAGTCATGGACATGGCTTCCATGTGGCGGCCTCCTCGAATCTCTCCGAGAGTCCCACGGACACCCACTCGACGACGCCGGTGCACCAGACCACCCACTCGCCGCACTCCCCGCTCCCGATCTCGCCGAGCACTGGCTCCGGCATTGGCCCGCTGGGCAATGTGTCCGCGGCAGCTGCGGCCGCTGCTCTCAACTCGAGCGGAGGCAGCAGTGTGGGTACCGCCGGCTCTGGGGGCGTGGCAACGAGCAAGACCACGCCCACGGGTAAGACGGGTCGGGCGCGTGGTAGACGCCATCAGCAGCCCAGCCCCACCAGAAGCACTGCCTCGGACACCGGGAACAGTGAGGCGGTGAAGCCACCGGAGCGGGTGTTCGTCTGGGATCTGGACGAGACGCTCATCATCTTCCACACGCTGCTGTCGGGCAGCTATGCCAACCGATACACCAAAGACCACAGCTCCCTGATGACCATCGCCTTCCGCATGGAGGAGATGGTCTTCAACATGGCCGACACGCATTTCTTCTTCAACGAGATCGAGGAGTGCGACCAGGTGCACATCGACGATGTCAGCTCGGACGACAATGGCCAGGACCTGAGCGCCTACAACTTCGCCACGGATGGCTTCCACACGAACACTCCACCAGGCGCCCCGCCCAATCTCTGCCTGCCCACCGGTGTGAGGGGCGGCGTCGATTGGATGCGCAAGCTGGCCTTCCGCTACCGCAAGATCAAGGACATCTACAATAGCTATCGTGGAAA

+18403 to +18785 (383bp) Intron 3 + Intron 4 + Intron 4

CTGCGGGAAATGGAAGGGAACAAAACAAAAACTGGCTATATCTCGGATCCATCCACCATCGCCATCCTCAACTCACCGATTTTGTGCGCACTGTAAATGTTCTCGATGTTGAAGATGCCGCCCAATCCGAACAGCAGGACCTTGGCCAGCGCCGGGGCCAGTTGCGTGGAGGTTACCAGCACGTTGACGCAGTTCTCCCGCTGGGAGATCATGCTCAGGCACTTGAGCGCCAGCGTGGCCCAGTTGTCGGTCGCCACCTCGATTTCCGAGCGTATCTGCAGCCAGGCCTCGCGTTTTCCGGGTCCCAGAAGGGTGCCAACACTGGAAGAACATTCGATCAGTTAGTAACCTATGAATCCGAAAGCGCATCCCCCAATACTCAC

+18933 to +19869 (937bp) Enhancer 4

CCCTCTACACTGCCCTTGACATGGGCTTCTTATGAAAGGCCAAACTGTAAGGGATTCGAAGCGGTTTTGAGTACAAACAGCAAAATGTTTAATTAATTTATTAAAATATGTATGTGTGTGTGTGCGTGTGAGACAAGCAACAAATGAAAACTGTAAACCAGCGCAAAATAATTTAATTATTTTGTTTAAACATTTATCATTTAACGCCAAGACTTTTTGTATTATATAGTTTTTAAACACCTAATCAACGATCGTAACAATTCTCGCACGAAGTTGTTCAAGTGTATAATTAACAAGTAAATAAATTAACGATATACATACATACGTACGTATTTAGCACCCTAGAGTAGCAAATAATAACAGACCGATACGCATCCTGGCTGGAGAAGCGGAGCAAACACAACAAAAATTAGTTTAAAGTTCTTAGTTTAAAAGCCGAAGCATAATTATAATGAGTATAAATAATTCGACAAAGCCGTAGTATTCAAATTTTAAATAACTATTATATAGCTGCATATATTAAACTATATTTAAAATATAAAACCAAGTAATAAAAGAGCAAATCCAACAGCAACGCACTCTATTAAAGCATAAGCCTGAGCATTTTTTATTGGGAAGGACTATGAGTAATATGGTCTAAATATGGTGATTTGTTCTTTAATCTTTCTTTGATAACTATGTTTTTGCACTAAACGTTTCTTACGTAATGTGTCACATGTTATATAAGAATAAAAAGGAATGGACAATGGAATTAATCCAATTTTGAGTTAAGATATTAATATATTCGGATTTAATGATGTGTAATTTTGTAAACTTACAATGTAAAAACCATTCTGTAGAATACGCATCAATGTATAAGTTTATGACATGAACTCCAGATGGCGCCCTTGCTGTTTTTACGCAGGCAGATTTAACCATTACTTACATGTACACTACA

319bp Spacer

GAATTCTGAAAGATCTCAATTAGCTAACCGAATATCTGTGGAAGTTTTAGTATGTTGGGTTTTGAATGGATGTATATCTCATGTATTTACGGCAGCTACAAATTCCTGGACCACACGCATATTGCATAACTCGCACAAAGTATATTTCACCTCAAATTAAAACCCGAAGAAAGAAGCAAAATCCTAGTCAATTCCTAGAGCTATTCAAACCTGACTCCTGGTGCTCTCAATCATATTTTCCACATCAAGTGCGCATAATTGCTATCCTTTAATGCGTTCCTCTAGCTCCGAAATGGCCAAAATGGTAGCAGTTGTCTAGA
