# Supplementary material for: Retinal Expression of the Drosophila eyes absent Gene Is Controlled by Several Cooperatively Acting Cis-regulatory Elements
Source: PLoS Genet. 2016 Dec 8;12(12):e1006462. doi: 10.1371/journal.pgen.1006462 (PMC5145141; doi:10.1371/journal.pgen.1006462)
Supplement: S2 Table — Sequences thar are listed in red were unable to drive expression of the lacZ reporter in the retina. Sequences listed in green define eya retinal enhancers. (DOCX) [file pgen.1006462.s009.docx]

Location: -8714 to -6333, Size: 2379bp, Cloning Type: RED StuI-XbaI, Vector: placZ.attB

5` primer: 5`ATA ATA AGG CCT TGT AAA CAT GTT GGC GAC TCG AAT TC-3`

3` primer: 5`ATA ATA TCT AGA GAT GGCCAT ATA GAC ATT GAC ATT GGG-3`

Location: -6332 to -3831, Size: 2501bp, Cloning Type: RED NotI-KpnI, Vector: placZ.attB

5` primer: 5`-ATA ATA GCG GCC GCA TCG GAA TAG AAA GCG TGT CTT TTA TG-3`

3` primer: 5`-ATA ATA GGT ACC CTT GGC CTG GCT GAT TCC CGA G-3`

Location: -3830 to -897, Size: 2934bp Enhancer 1 Large Fragment, Cloning Type: RED HindIII-KpnI Vector: placZ.attB

5` primer: 5`-ATA ATA AAG CTT ACT ACA CCT CGT ACC AAA TTC TCG G-3`

3` primer: 5`-ATA ATA GGT ACC GGC CAG TTT CGT CTC CTC TTT TGC-3`

Location: -1171 to -897, Size: 275bp, Enhancer 1 Minimal Fragment, Cloning Type: Gateway, Vectors: pglacZ.attB and pg-eyaRB cDNA.attB

5` primer: 5`-AAA TAT TTG GAT ATG TGG GGG AAA GGG-3`

3` primer: 5`-CAG TTT CGT CTC CTC TTT TGC-3`

Location: -896 to -577, Size: 319bp, Enhancer E, Cloning Type RED HindIII-KpnI, Vectors: placZ.attB and p-eyaRB cDNA.attB

5` primer: 5`-ATA ATA AAG CTT CCG TAT GTC TCC TAG TCT GCC CTG TG-3`

3` primer: 5`-ATA ATA GGT ACC TTA ACT GAC CTG CTC AAC TCA AAT CCG-3`

Location: -576 to +10, Size: 586bp, Enhancer 2 Large Fragment, Cloning Type RED HindIII-KpnI, Vector: placZ.attB

5` primer: 5`-ATA ATA AAG CTT ATT TGA GTT GAG CAG GTC AGT TAA TAT TAC-3`

3` primer: 5`-ATA ATA GGT ACC TCA ACT GAT TCG ACT TGG TCG-3`

Location: -576 to +50, Size: 626 Enhancer 2 in Promoterless Vector, Cloning Type: RED HindIII-KpnI and Gateway, Vectors: pglacZ.attB w/o hsp70 promoter

5` primer: 5`-ATA ATA AAG CTT ATT TGA GTT GAG CAG GTC AGT TAA TAT TAC- 3`

5` primer: 5`-ATT TGA GTT GAG CAG GTC AGT TAA TAT-3`

3` primer: 5`-ATA ATA GGT ACC GTG GTC CGC TTT CGC CAC GAA TGT CAC ATA AAG CTG CGT GTC AAC TGA TTC GAC TTG GTC G-3`

3` primer: 5`-GTG GTC CGC TTT CGC CAC GAA TG-3`

Location: -1171 to +10, Size: 1181bp, Composite Enhancer, Cloning Type: Gateway, Vector: pglacZ.attB and pg-eyaRB cDNA.attB

5` primer: 5`-AAA TAT TTG GAT ATG TGG GGG AAA GGG-3`

3` primer: 5`-TCA ACT GAT TCG ACT TGG TCG AAA AGC-3`

Location: -1171 to +50, Size: 1221bp, Composite Enhancer in Promoterless Vector, Cloning Type: RED HindIII-KpnI and Gateway, Vector: pglacZ.attB w/o hsp70 promoter

5` primer: 5`-ATA ATA AAG CTT AAA TAT TTG GAT ATG TGG GGG AAA GGG-3`

5` primer: 5`-AAA TAT TTG GAT ATG TGG GGG AAA GGG-3`

3` primer: 5`-ATA ATA GGT ACC GTG GTC CGC TTT CGC CAC GAA TGT CAC ATA AAG CTG CGT GTC AAC TGA TTC GAC TTG GTC G-3`

3` primer: 5`-GTG GTC CGC TTT CGC CAC GAA TG-3`

Location: +529 to +4272, Size: 3744bp, Intron 1-1, Cloning Type: RED StuI-XbaI, Vector: placZ.attB

5` primer: 5`-ATA ATA AGG CCT GTA AGT TGA AAG ATC TCA ATT AGC TAA CCG-3`

3` primer: 5`-ATA ATA TCT AGA TCT AAG AAA AAC TGT CAC GAC TGC TAA CG-3`

Location: +3957 to +7672, Size: 3698bp, Intron 1-2, Cloning Type: RED EcoRI-XbaI, Vector: placZ.attB

5` primer: 5`-ATA ATA GAA TTC GTG AAA ACA AAA CTT GCG CCT CAT TTC C-3`

3` primer: 5`-ATA ATA TCT AGA GGG CGT TTG GCT GGG TCT TGA TAC AC-3`

Location: +7330 to +11076, Size: 3747bp, Enhancer 3 Large Fragment, Cloning Type: RED NotI-KpnI, Vector: placZ.attB

5` primer: 5`-ATA ATA GCG GCC GCT TTG CTT CGC CTC GGT CAC TAT GGC-3`

3` primer: 5`-ATA ATA GGT ACC TGT GGA CTT GCG ATT ACT TTC ACT TGC-3`

Location: +10576 to +11076, Size: 500bp, Enhancer 3 Minimal Fragment, Cloning Type: Gateway, Vectors: pglacZ.attB and pg-eyaRB cDNA.attB

5` primer: 5`-CAC CCC TGT GCG ATA TAC TTG GC-3`

3` primer: 5`-CCT GTG GAC TTG CGA TTA CTT TCA C-3`

Location: +11149 to +14024, size: 2872bp, Intron 1-4, Cloning Type: RED StuI, Vector: placZ.attB

5` primer: 5`-ATA ATA AGG CCT GTA ATC ACA TAG TCG TAG TGC TCT CC-3`

3` primer: 5`-ATA ATA AGG CCT CGT GTG GTC TGT CTT GGG ACG TTT AAC-3`

Location: +14072 to +16647, Size: 2576bp, Intron 2, Cloning Type: RED HindIII-XbaI, Vector: placZ.attB

5` primer: 5`-ATA ATA AAG CTT CCA CAC GGA TAC ACA TGA ACG CAA CC-3`

3` primer: 5`-ATA ATA TCT AGA CTG CAA GAA GAT AAG ATG AGA GGC GTC-3`

Location: +16648 to +18402, Size: 1749bp, Exon 3, Cloning Type: Gateway, Vector: pglacZ.attB

5` primer: 5`-CAA TCT GTC ACA GCA GCA GCA GC-3`

3` primer: 5`-TTT CCA CGA TAG CTA TTG TAG ATG TCC-3`

Location: +18403 to +18785, Size: 383bp, Intron 3 + Exon 4 + Intron 4, Cloning Type: RED HindIII-XbaI, Vector: placZ.attB

5` primer: 5`-ATA ATA AAG CTT GTG AGT ATT GGG GGA TGC GCT TTC G-3`

3` primer: 5`-ATA ATA TCT AGA GAT CCG CTC ATA GCA GGT TTC ATG GC-3`

Location: +18933 to +19869, Size: 937bp, Enhancer 4, Cloning Type: Gateway, Vectors: pglacZ.attB and pg-eyaRB cDNA.attB

5` primer: 5`-CCC TCT ACA CTG CCC TTG ACA TGG-3`

3` primer: 5`-TGT AGT GTA CAT GTA AGT AAT GGT TAA-3`

Location: +535 to +840, Spacer, Size: 319bp, Cloning Type: Gateway, Vectors: pglacZ.attB and pg-eyaRB cDNA.attB

5` primer: 5`-GAA TTC TGA AAG ATC TCA ATT AGC TAA-3`

3` primer: 5`-TCT AGA CAA CTG CTA CCA TTT TGG CC-3`

eya RB+3’UTR cDNA, Cloning Type: RED EcoRI-NdeI

5` primer: 5`-ATA ATA GAA TTC ATG TTG TAT AAT GTG CCG TGC TAT C-3`

3` primer: 5`-ATA ATA CAT ATG AGG CTT ATG CTT TAA TAG AGT GCG TTG C-3`

Gateway Cassette and hsp70 promoter from pg-RFP.attB, Cloning Type: RED EcoRI

5` primer: 5`-ATA ATA GAA TTC CGT ATG GCA ATG AAA GAC GGT G-3`

3` primer: 5`-ATA ATA GAA TTC GGG TGT GAG TTC TTC TTC TTT CTC GGG-3`
