# Supplementary material for: Retinal Expression of the Drosophila eyes absent Gene Is Controlled by Several Cooperatively Acting Cis-regulatory Elements
Source: PLoS Genet. 2016 Dec 8;12(12):e1006462. doi: 10.1371/journal.pgen.1006462 (PMC5145141; doi:10.1371/journal.pgen.1006462)
Supplement: S3 Table — (DOCX) [file pgen.1006462.s010.docx]

| **Genomic location** | **Primer sequence** |
| --- | --- |
| -2627 to -2610 | 5`-TTATCCGAAAGGAGGACC-3` |
| -2208 to -2187 | 5`-ATGAGCAAAGTCACTCCCCTCC-3` |
| -1730 to -1711 | 5`-TGGAGTAGTTGGGCAGGTTG-3` |
| -1270 to -1253 | 5`-CATTGAGTGCTTTTCCGC-3` |
| -734 to -711 | 5`-AAAGAGGAGACCGAGACCCAGAAC-3` |
| -193 to -176 | 5`-AAAAGCGAGTCCTGATGC-3` |
| 385 to 406 | 5`-CCATTTGACATTTCCACTGTGC-3` |
| 891 to 909 | 5`-TGCTACTACGCATTTGCCC-3` |
| -2536 to -2518 | 5`-AATCACTTGGCATCCGAGC-3` |
| -1950 to -1932 | 5`-GCTGGAAGTTGACCGATTC-3` |
| 728 to 748 | 5`-CAGGAGTCAGGTTTGAATAGC-3` |
| 1083 to 1102 | 5`-CCATTGAGTTACAGCCCTTG-3` |
