# Supplementary material for: Retinal Expression of the Drosophila eyes absent Gene Is Controlled by Several Cooperatively Acting Cis-regulatory Elements
Source: PLoS Genet. 2016 Dec 8;12(12):e1006462. doi: 10.1371/journal.pgen.1006462 (PMC5145141; doi:10.1371/journal.pgen.1006462)
Supplement: S4 Table — (DOCX) [file pgen.1006462.s011.docx]

| **Name** | **Sequence** | **Reference** |
| --- | --- | --- |
|  |  |  |
| so F | 5`-GCCTGTGTTTGCGAGGTTCT-3` | Fly PrimerBank* |
| so R | 5`-TGCAGCTTATCACATTGTGGC-3` |  |
|  |  |  |
| eyaI F | 5`-AATGCCATACAACTACGCTGC -3` | ApE |
| eyaI R | 5`-GTATCCGTGTGGTCTGTCTTG-3` |  |
|  |  |  |
| eyaII F | 5`-GGAGCAGCCACAACACTTG-3` | ApE |
| eyaII R | 5`-CGTGTGGTCTGTCTTGGGA-3` |  |
|  |  |  |
| eyaI F endo | 5`-CGCAAGTCCACAGAATGGTCAC-3` | ApE |

* [G3 (Bethesda). 2013 Sep 4;3(9):1607-16. doi: 10.1534/g3.113.007021.](https://www.ncbi.nlm.nih.gov/pubmed/23893746)
