# Supplementary material for: Retinal Expression of the Drosophila eyes absent Gene Is Controlled by Several Cooperatively Acting Cis-regulatory Elements
Source: PLoS Genet. 2016 Dec 8;12(12):e1006462. doi: 10.1371/journal.pgen.1006462 (PMC5145141; doi:10.1371/journal.pgen.1006462)
Supplement: S5 Table — Included in this list are the positions of So binding sites, So ChIP peaks, position of retinal enhancers, as well as the position of introns and exons. (DOCX) [file pgen.1006462.s012.docx]

Eya locus -8714 to 19869: Osm-6 to Insulator

So Chip-seq peaks in purple

| **Coordinates** | **Position on Schematic** | **Additional Notes** |
| --- | --- | --- |
| -8714 to -6333 | Most upstream fragment (no expression) |  |
| **-8714 to -7088** | **So Chip-seq Peak #1** | **Contained within and beyond most upstream fragment also covers PSE enhancer** |
| **-8089 to -8080** | **So binding site (Yan B)** | **Contained within most upstream fragment** |
| **-8088 to -8082** | **So binding site (Berger B)** | **Contained within most upstream fragment** |
| **-7884 to -7150** | **PSE Enhancer** | **Contained within most upstream fragment** |
| **-7565 to -7560** | **So binding site (Hazbun B)** | **Contained within PSE Enhancer** |
| -6332 to -3831 | 2^nd^ upstream fragment (no expression) |  |
| **-5765 to -5759** | **So binding site (Berger A)** | **Contained within 2^nd^ upstream fragment** |
| **-5764 to -5759** | **So binding site (Noyes, Hazbun A)** | **Contained within 2^nd^ upstream fragment** |
| **-5565 to -5560** | **So binding site (Noyes, Hazbun A)** | **Contained within 2^nd^ upstream fragment** |
| **-4460 to -4455** | **So binding site (Noyes, Hazbun A)** | **Contained within 2^nd^ upstream fragment** |
| **-3830 to -897** | **Enhancer #1 large fragment** |  |
| **-1470 to -1465** | **So binding site (Hazbun B)** | **Located outside of minimal enhancer #1 fragment** |
| **-1171 to -897** | **Enhancer #1 minimal fragment** |  |
| **-896 to -577** | **Extant Enhancer** |  |
| **-614 to -609** | **So binding site (Hazbun B)** | **Contained within Extant Enhancer** |
| **-576 to 10** | **Enhancer #2 large fragment** |  |
| **-448 to 396** | **So Chip-seq Peak #2** | **Contained within enhancer #2 and 5’ UTR of RB** |
| **-282 to 10** | **Enhancer #2 minimal fragment** |  |
| 1 to 474 | 5’ UTR eya RB | NOT A CONSTRUCT |
| 475 to 528 | Exon 1 | 53 bp in size NOT A CONSTRUCT |
| 529 to 14024 | Intron 1 | Divided into 4 pieces due to size |
| 529 to 4272 | Intron 1-1 fragment (no expression) |  |
| **3548 to 5986** | **So Chip-seq Peak #3** | **Spans Intron 1-1 and 1-2 fragments and So site in 1-2** |
| 3957 to 7672 | Intron 1-2 fragment (no expression) | Overlaps slightly with Intron 1-1 fragment |
| **4979 to 4984** | **So binding site (Noyes, Hazbun A)** | **Contained within Intron 1-2 fragment** |
| **6857 to 6862** | **So binding site (Hazbun B)** | **Contained within Intron 1-2 fragment** |
| **7330 to 11076** | **Enhancer #3 large fragment** | Overlaps slightly with Intron 1-2 fragment |
| **7430 to 7435** | **So binding site (Noyes, Hazbun A)** | **Outside of minimal Enhancer #3 fragment** |
| **7649 to 7654** | **So binding site (Noyes, Hazbun A)** | **Outside of minimal Enhancer #3 fragment** |
| **9875 to 11376** | **So Chip-seq Peak #4** | **Outside and within minimal Enhancer #3 fragment** |
| **10137 to 10142** | **So binding site (Hazbun B)** | **Outside of miminal Enhancer #3 fragment but within Chip-seq peak #3** |
| **10576 to 11076** | **Enhancer #3 minimal fragment** |  |
| 11077 to 11148 | Exon 1A | 71 bp in size NOT A CONSTRUCT |
| 11149 to 14024 | Intron 1-4 fragment (no expression) |  |
| **11444 to 11450** | **So binding site (Berger B)** | **Contained within Intron 1-4 fragment** |
| 14025 to 14071 | Exon 2 | 46 bp NOT A CONSTRUCT |
| 14072 to 16647 | Intron 2 (no expression) |  |
| 16648 to 18402 | Exon 3 (no expression) | 1754 bp in size THIS IS A CONSTRUCT |
| **18080 to 18085** | **So binding site (Hazbun B)** | **Contained within Exon 3 fragment** |
| 18403 to 18464 | Intron 3 (no expression) | Combined with Exon 4 and Intron 4 due to small size |
| 18465 to 18709 | Exon 4 (no expression) | 244 bp in size part of a construct |
| 18710 to 18785 | Intron 4 (no expression) | Combined with Intron 3 and Exon 4 due to small size |
| 18786 to 18967 | Exon 5 | 181 bp in size NOT A CONSTRUCT |
| **18933 to 19869** | **Enhancer #4** | **Overlaps part of Exon 5** |
| 18968 to 19530 | 3’ UTR | Contained within Enhancer #4 |
| **19298 to 19303** | **So binding site (Hazbun B)** | **Contained within Enhancer #4 in 3’ UTR** |
| 19531 to 19869 | End of 3’UTR to genomic insulator | Contained within Enhancer #4 |
